# Effectiveness of Varenicline and Cytisine for Alcohol Use Reduction Among People With HIV and Substance Use: A Randomized Clinical Trial

**DOI:** 10.1001/jamanetworkopen.2022.25129

**Published:** 2022-08-05

**Authors:** Hilary A. Tindle, Matthew S. Freiberg, Debbie M. Cheng, Natalia Gnatienko, Elena Blokhina, Tatiana Yaroslavtseva, Sally Bendiks, Gregory Patts, Judith Hahn, Kaku So-Armah, Michael D. Stein, Kendall Bryant, Dmitry Lioznov, Evgeny Krupitsky, Jeffrey H. Samet

**Affiliations:** 1 Vanderbilt Center for Tobacco, Addiction and Lifestyle, Vanderbilt University Medical Center, Division of Internal Medicine and Public Health, Nashville, Tennessee; 2 Vanderbilt Center for Clinical Cardiovascular Trials Evaluation, Cardiovascular Division, Vanderbilt University Medical Center, Nashville, Tennessee; 3 Department of Biostatistics, Boston University School of Public Health, Boston, Massachusetts; 4 Section of General Internal Medicine, Clinical Addiction Research and Education Unit, Department of Medicine, Boston Medical Center, Boston, Massachusetts; 5 First Pavlov State Medical University of St Petersburg, St Petersburg, Russian Federation; 6 Biostatistics and Epidemiology Data Analytics Center, Boston University School of Public Health, Boston, Massachusetts; 7 Department of Medicine, University of California, San Francisco; 8 Section of General Internal Medicine, Department of Medicine, Boston University School of Medicine, Boston, Massachusetts; 9 Clinical Addiction Research and Education Unit, Boston Medical Center, Boston, Massachusetts; 10 Department of Health Law Policy and Management, Boston University School of Public Health, Boston, Massachusetts; 11 HIV/AIDS Research, National Institute on Alcohol Abuse and Alcoholism, National Institute of Health, Bethesda, Maryland; 12 Department of Addictions, V.M. Bekhterev National Medical Research Center for Psychiatry and Neurology, St Petersburg, Russian Federation; 13 Department of Community Health Sciences, Boston University School of Public Health, Boston, Massachusetts

## Abstract

**Importance:**

Cigarette smoking and risky alcohol consumption co-occur and are undertreated. Nicotine receptor partial agonists and nicotine replacement therapy (NRT) treat smoking but are unproven for alcohol, and clinical trials rarely include individuals with HIV, substance use, and mental health conditions.

**Objective:**

To compare the effects on drinking and smoking of nicotinic acetylcholine receptor partial agonists varenicline and cytisine with those of NRT.

**Design, Setting, and Participants:**

This 4-group randomized, double-blinded, placebo-controlled clinical trial was conducted from July 2017 to December 2020 in St Petersburg, Russia. Included participants were 400 individuals with HIV who engaged in risky drinking (≥5 prior-month heavy-drinking days [HDDs]) and daily smoking; they were followed up for 12 months after enrollment. Data were analyzed from May 2021 through June 2022.

**Interventions:**

Participants received alcohol and tobacco counseling, 1 active medication, and 1 placebo in 1 of 4 groups: active varenicline and placebo NRT (group 1), placebo varenicline and active NRT (group 2), active cytisine and placebo NRT (group 3), or placebo cytisine and active NRT (group 4).

**Main Outcomes and Measures:**

The primary outcome was number of prior-month HDDs at 3 months. Secondary outcomes included biochemically validated abstinence from alcohol at 3 months and smoking at 6 months.

**Results:**

Among 400 participants (263 [65.8%] men; mean [SD] age, 39 [6] years), 97 individuals (24.3%) used opioids and 156 individuals (39.1%) had depressive symptoms. These individuals had a mean (SD) CD4 count of 391 (257) cells/mm^3^, smoked a mean (SD) of 21 [8] cigarettes/d, and reported a mean (SD) of 9.3 (5.8) HDDs in the prior 30 days. At 3 months, the mean (SD) number of HDDs was decreased vs baseline across all groups (group 1: 2.0 [3.8] HDDs vs. 9.5 [6.1] HDDs; group 2: 2.1 [4.3] HDDs vs 9.3 [5.7] HDDs; group 3: 1.5 [3.3] HDDs vs 8.9 [5.0] HDDs; group 4: 2.4 [5.2] HDDs vs 9.6 [6.3] HDDs). There were no significant differences at 3 months between groups in mean (SD) HDDs, including group 1 vs 2 (incident rate ratio [IRR], 0.94; 95% CI, 0.49-1.79), 3 vs 4 (IRR, 0.60; 95% CI, 0.30-1.18), and 1 vs 3 (IRR, 1.29; 95% CI, 0.65-2.55). There were no significant differences at 6 months between groups in smoking abstinence, including group 1 vs 2 (15 of 100 individuals [15.0%] vs 17 of 99 individuals [17.2%]; odds ratio [OR],0.89; 95% CI, 0.38-2.08), 3 vs 4 (19 of 100 individuals [19.0%] vs 19 of 101 individuals [18.8%]; OR, 1.00; 95% CI, 0.46-2.17), and 1 vs 3 (OR, 0.79; 95% CI, 0.35-1.78). Post hoc analyses suggested lower mean (SD) HDDs (eg, at 3 months: 0.7 [1.8] HDDs vs 2.3 [4.6] HDDs) and higher alcohol abstinence (eg, at 3 months: 30 of 85 individuals [35.3%] vs 54 of 315 individuals [17.1%]) among those who quit vs continued smoking.

**Conclusions and Relevance:**

This study found that among individuals with HIV who engaged in risky drinking and smoking, varenicline and cytisine were not more efficacious than NRT to treat risky drinking and smoking but that behavior change rates were high in all groups.

**Trial Registration:**

ClinicalTrials.gov Identifier: NCT02797587

## Key Points

**Question:**

Are varenicline and cytisine more efficacious than nicotine to treat alcohol and smoking among individuals who smoke daily, have HIV, and engage in risky drinking?

**Findings:**

This randomized clinical trial of 400 individuals with HIV who engaged in risky drinking and daily smoking found that alcohol consumption at 3 months was decreased in all groups (active varenicline and placebo nicotine replacement therapy [NRT], placebo varenicline and active NRT, active cytisine and placebo NRT, and placebo cytisine and active NRT) but heavy drinking days did not differ significantly by group. Smoking abstinence at 6 months did not differ by group, but abstinence rates were high.

**Meaning:**

This study found that varenicline and cytisine were not more efficacious than nicotine to treat risky drinking and smoking but that rates of behavior change for alcohol consumption and smoking were high in all treatment groups, suggesting a role for these medications in individuals who report risky drinking and smoking.

## Teaser

This randomized clinical trial compares the effectiveness of varenicline and cytisine with nicotine replacement therapy for reducing alcohol consumption among individuals with HIV who smoke daily and engage in risky drinking.

## Introduction

The co-occurrence of cigarette smoking and unhealthy alcohol use^1^ is common^2^ and detrimental to health.^3^ Among people with HIV infection, these behaviors are associated with increased risk of non-AIDS diseases.^4^ Nicotine replacement therapy (NRT) and varenicline, a partial agonist of the α4β2 nicotinic acetylcholine receptor, are effective for smoking cessation, including among individuals with HIV.^5,6,7^ Studies among smokers who drink alcohol demonstrate that varenicline reduces alcohol craving and consumption compared with placebo.^8,9^ Cytisine, a plant-based partial agonist of the α4β2 nicotinic acetylcholine receptor, is effective for smoking cessation^10,11,12^ and similar in efficacy to varenicline,^13,14^ but its effects on alcohol consumption in humans are unknown.^15^ Converging data support a role of nicotinic acetylcholine receptors in the consumption of alcohol, suggesting that they may serve as pharmacotherapeutic targets to treat risky drinking in addition to their established role in treating smoking.^16,17,18,19^

Whether nicotinic receptor partial agonists are uniquely positioned to target alcohol consumption independent of their effects on smoking cessation is unclear. Prior studies examining varenicline for risky drinking^9^ have compared varenicline only with placebo and not a medication control, such as NRT, which has no demonstrated efficacy for alcohol. Furthermore, these studies typically did not include participants with multimorbidity, polysubstance use, HIV, biochemical validation of alcohol consumption (for exception, see de Bejczy et al^20^), or long-term follow-up (for exception, see Bold et al^21^). To our knowledge, there is no study examining the efficacy of cytisine for alcohol consumption or examining cytisine for smoking cessation among individuals with HIV. Additionally, to our knowledge, there is no comparative effectiveness study examining varenicline, cytisine, and NRT for smoking cessation.

Studying Partial Agonists for Ethanol and Tobacco Elimination in Russians with HIV (St PETER HIV) is a 4-group randomized, double-blinded, placebo-controlled, clinical trial among 400 individuals with HIV who were daily smokers with risky alcohol use. This study was designed to compare the efficacy of varenicline and cytisine with NRT for alcohol consumption (primary outcome) and smoking cessation (secondary outcome). We hypothesized that varenicline and cytisine would be more efficacious in reducing alcohol consumption and smoking compared with NRT.

## Methods

The protocol for St PETER HIV has been previously described^22^ (see trial protocol in Supplement 1). This randomized clinical study was approved by institutional review boards at Boston University Medical Campus, Vanderbilt University Medical Center, and First St Petersburg Pavlov State Medical University. Participant safety data were reviewed by a data safety and monitoring board every 6 months. All study participants provided written informed consent. This study was registered with ClinicalTrials.gov through the National Institutes of Health (NCT02797587). This report follows the Consolidated Standards of Reporting Trials (CONSORT) reporting guideline.

### Participants

We recruited 400 participants from HIV clinical care sites in St Petersburg, Russia (see study protocol in Supplement 1). Follow-up was from July 2017 through December 2020. Enrollment and assessment activities were conducted by trained research medical personnel (T.Y.) at First Pavlov State Medical University. Eligibility criteria included the following: being ages 18 to 70 years, being HIV positive, engaging in 5 or more heavy drinking days (HDDs; defined as US National Institute on Alcohol Abuse and Alcoholism at-risk drinking levels [ie, risky drinking])^23^ in the prior 30 days, smoking a mean number of 5 or more cigarettes/d, and having a willingness to reduce alcohol consumption, smoking, or both. Participants were excluded if they were not fluent in Russian, were pregnant or planning to become pregnant or breastfeeding, had taken smoking cessation medications in the prior 30 days, or had cognitive impairment resulting in an inability to provide informed consent, unstable psychiatric illness, a history of seizures, or a known allergy to study medications.

### Randomization and Masking

**Figure 1.**
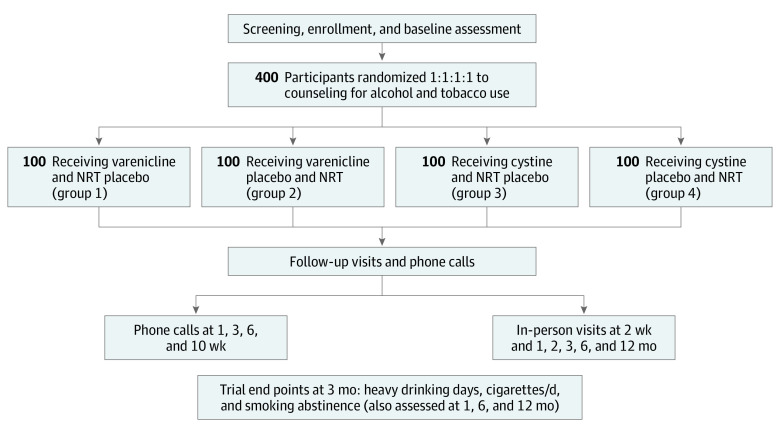
Trial Design NRT indicates nicotine replacement therapy.

Participants were randomly assigned in a 1:1:1:1 ratio to 1 of 4 study groups: active varenicline and placebo NRT (group 1), placebo varenicline and active NRT (group 2), active cytisine and placebo NRT (group 3), or placebo cytisine and active NRT (group 4) (Figure 1). Randomization was stratified by 3 factors: alcohol use (≥3 vs <3 HDDs in the prior week),^23^ mean daily cigarette use (≤1 vs >1 pack/d), and current antiretroviral therapy (ART) use (yes or no). Study participants and staff were blinded to randomization assignment of participants.

### Counseling, Target Quit Date, and Study Medication Regimens

At baseline, participants received brief guideline-based counseling on alcohol^23^ and smoking^24^ (eFigure 1 in Supplement 2). Participants were advised to begin reducing their number of cigarettes smoked/d and quit smoking on the target quit date 1 week after enrollment. All participants received study pills (active or placebo) and a study mouth spray (active NRT or placebo) (eFigure 1 in Supplement 2). Placebo and active study medications were indistinguishable by appearance and taste. In varenicline groups (eFigure 1 in Supplement 2), participants randomized to active varenicline and NRT placebo (group 1) received 12 weeks of active varenicline and 8 weeks of NRT placebo mouth spray. Participants randomized to varenicline placebo and active NRT (group 2) received 12 weeks of placebo varenicline and 8 weeks of active NRT mouth spray. Varenicline dosing followed recommended levels.^25^ NRT mouth spray contained 1 mg of nicotine per spray. Participants were instructed to use 8 sprays/d as a minimum for the first 4 weeks and to use the spray as needed during weeks 5 through 8 up to the maximum recommended daily sprays to control cravings. In cytisine groups (eFigure 1 in Supplement 2), participants randomized to active cytisine and NRT placebo (group 3) received a 25-day regimen of active cytisine and 8 weeks of NRT placebo mouth spray. Cytisine dosing followed the traditional 25-day downward titration schedule of 1.5 mg tablets: 6 tablets/d (days 1-3), 5 tablets/d (days 4-12), 4 tablets/d (days 13-16), 3 tablets/d (days 17-20), and 1 to 2 tablets/d (days 21-25).^11^ Participants randomized to cytisine placebo and active NRT (group 4) received a 25-day regimen of placebo cytisine and 8 weeks of active NRT mouth spray.

### Adherence

The primary measure used to assess medication adherence was a visual analog scale at each study visit. Participants were asked to indicate the number that best reflected how much study medication they had taken in the prior week. Participants were considered adherent to study pills if they self-reported taking at least 80% of their assigned study medication at 2 of 3 study visits prior to the 1-month visit for cytisine and at 4 of 7 study visits prior to the 3-month visit for varenicline. Participants were considered adherent to study spray if (1) during weeks 1 through 4 of treatment, they self-reported using at least 8 sprays/d at least 80% of the days in the last week at 2 of 3 study visits and (2) during weeks 5 through 8 of treatment, they used the spray at least 80% of times when they had an urge to smoke on at least 1 of 2 study visits prior to the 2-month visit.

### Participant Assessment

Participants were interviewed at baseline and 1-, 3-, 6-, and 12-month study visits with shorter phone assessments involving medication check-ins at 1, 2, 3, 6, 8, and 10 weeks. At all visits, participants were assessed for possible adverse events. Participants were compensated for their time in amounts ranging from 200 rubles (approximately $15 US) for telephone visits, 2000 rubles (approximately $33 US) for longer in-person study visits, and 2500 rubles (approximately $40 US) for the final study visit at 12 months.

Questionnaire data collected during longer assessment visits included demographics and 30-day alcohol and cigarette timeline follow-back (TLFB),^26^ Alcohol Use Disorders Identification Test (AUDIT; score range, 0-12),^27^ Penn Alcohol Craving Questionnaire (PACS; score range, 0-30),^28^ readiness to quit smoking and drinking (Likert scales; score ranges, 1-10 and 0-10, respectively),^29,30^ Fagerström Test for Nicotine Dependence (score range, 0-10),^31^ smoking quit attempts, depressive symptoms (Center for Epidemiologic Studies-Depression; score range, 0-60),^32^ anxiety (General Anxiety Disorder-7; score range, 0-21),^33^ ART use and adherence^34^; and other substance use.^35,36^

Blood was collected from participants at baseline and 1, 3, 6, and 12 months for storage and testing. At baseline and 3 months, fresh blood was tested for CD4 cell count, hepatitis C antibody (baseline only), and HIV RNA. Stored samples were tested for phosphatidylethanol (PEth), a direct biomarker associated with alcohol, at baseline and 3 months. Urine was collected at study visits up to and including the 3-month visit to administer a pregnancy test for all women.

### Primary Outcome

The primary outcome was percentage of HDDs in the prior 30 days, assessed at 3 months using self-reported 30-day alcohol consumption obtained via the TLFB method.^37^ Transformations of the outcome were allowed by the study protocol if needed to meet modeling assumptions (Supplement 1). The distribution of percentage of HDDs was nonnormal despite transformations. We therefore transformed percentage of HDDs to number of HDDs in the prior 30 days at 3 months and modeled it as count data using negative binomial regression.

### Secondary Outcomes

We assessed 2 smoking-related secondary outcomes at 3, 6, and 12 months: number of cigarettes/d in the prior 7 days via TLFB and self-reported 7-day point prevalence abstinence from smoking validated via end-expired carbon monoxide levels of less than 10 ppm.^38,39^ Alcohol-related secondary outcomes included number of HDDs and self-reported 30-day alcohol abstinence at 6 and 12 months. Biochemically validated alcohol abstinence at 3 months (PEth < 8 ng/mL and self-report of no alcohol consumption in the prior 30 days) were post hoc exploratory outcomes.^40^

### Statistical Analysis

The St PETER HIV study was designed to have 80% power to detect a difference of 14.1 percentage points in percentage of HDDs for any of 3 pairwise comparisons of interest (varenicline vs NRT, cytisine vs NRT, and varenicline vs cytisine) using a 2-sided *t* test. An SD in percentage of HDDs of 27.2% was assumed based on our prior work.^41^ This trial was conducted and analyzed according to the intention-to-treat principle. The main analysis evaluating the impact of interventions on the primary study outcome used a negative binomial regression model controlling for stratification factors (alcohol consumption, mean daily number of cigarettes, and ART use at baseline). The models included indicator variables to represent study group. We adjusted the 3 pairwise comparisons for multiple comparisons using the Hochberg sequential test procedure. Negative binomial regression was used to model count outcomes. Binary outcomes (eg, abstinence from alcohol) were analyzed using logistic regression models. For all modeled outcomes, we accounted for missing data with multiple imputation by predictive mean matching.^42^ Variables included in the imputation model were education, cigarettes/d, time to first cigarette daily, years smoked, history of quit attempts, living with a smoker (yes or no), readiness to quit smoking and drinking, depressive symptoms, anxiety score, sex, age, opioid use status, viral load, CD4 cell count, mean number of drinks/d in the prior 30 days, alcohol use disorder category, baseline HDDs in the prior 30 days, and AUDIT score of 20 or more. A secondary per-protocol analysis was conducted that included only participants who were adherent to their assigned intervention (ie, pills and spray). Lastly, we conducted exploratory analyses that compared combined partial agonists vs NRT. SAS statistical software version 9.4 (SAS Institute) was used in analysis, and 2-tailed tests and an overall significance level of 0.05 were used for all tests. Authors conducted analysis of the data from May 2021 through June 2022.

## Results

**Figure 2.**
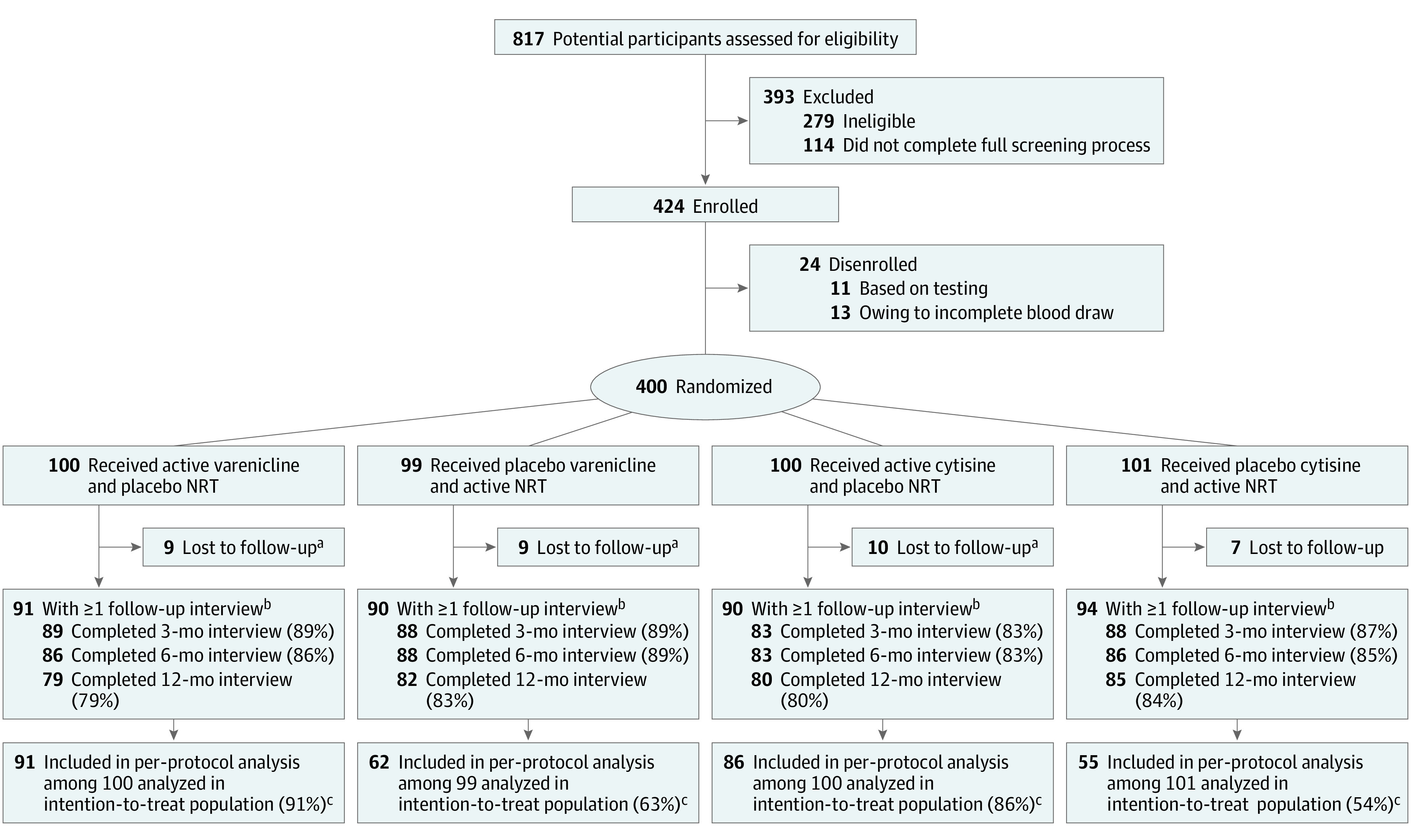
Flow of Participants Through Trial NRT indicates nicotine replacement therapy. ^a^Lost to follow-up includes participants who missed all follow-up visits (months 3, 6, and 12). ^b^There were 4 deaths in group 1, 3 deaths in group 2, and 2 deaths in group 3, and there was 1 death in group 4. Death counts include those who died before follow-up or after any follow-up visit. ^c^All participants randomized were included in analysis, per intention to treat. Multiple imputation was performed to impute outcomes for participants who missed follow-up interviews. Per-protocol analysis was limited to participants who were adherent to their assigned intervention. The per-protocol analysis used the imputed data set, thus making it possible to include adherent participants who were lost to follow-up after adherence had been determined in the first 10 weeks.

At baseline, 400 participants (mean [SD] age, 39 [6] years; 263 [65.8%)] men) smoked a mean (SD) of 21 (8) cigarettes/d, reported a mean (SD) of 26 (13) pack-years, and reported risky drinking on a mean (SD)] of 9.3 (5.8) days in the prior 30 days (Table 1). A total of 817 individuals were screened and 424 individuals were enrolled (Figure 2) between July 19, 2017, and December 25, 2019. Of those participants, 24 individuals were ineligible for randomization (eg, owing to negative rapid HIV tests). At baseline, 400 randomized participants were interested in reducing drinking and smoking (320 participants), drinking only (2 participants), or smoking only (78 participants), with mean (SD) readiness of 4.8 (2.5) for drinking and 6.6 (1.0) for smoking (Table 1). Most participants (337 individuals [84.5%]) had a history of hepatitis C virus infection, while 195 participants (48.8%) had severe alcohol use disorder, 156 participants (39.1%) had depressive symptoms, 54 individuals (13.6%) had at least moderate anxiety, and 97 participants (24.3%) reported prior 30-day opioid use. Most participants (291 individuals [72.8%]) were on ART, while 228 participants (57.0%) were virally suppressed (<300 copies/mL), and the mean [SD] CD4+ T cell count was 391 (257) cells/mm^3^. Overall, groups were balanced across baseline characteristics (Table 1).

**Table 1.** Baseline Characteristics

Characteristic	Participants, No. (%)^a^^,^^b^				
	Varenicline group		Cytisine group		Overall (N = 400)
	Group 1 (n = 100)	Group 2 (n = 99)	Group 3 (n = 100)	Group 4 (n = 101)	
Sex					
Women	34 (34.0)	38 (38.4)	31 (31.0)	34 (33.7)	137 (34.3)
Men	66 (66.0)	61 (61.6)	69 (69.0)	67 (66.3)	263 (65.8)
Age, mean (SD), y	39 (6)	39 (6)	38 (6)	39 (7)	39 (6)
≥Basic education^c^	98 (98.0)	97 (98.0)	94 (94.0)	101 (100)	390 (97.5)
Depressive symptoms^d^	34 (34.0)	39 (39.4)	38 (38.4)	45 (44.6)	156 (39.1)
≥Moderate anxiety^e^	12 (12.1)	17 (17.3)	10 (10.2)	15 (14.9)	54 (13.6)
HCV co-infection	85 (85.0)	82 (82.8)	86 (86.0)	84 (84.0)	337 (84.5)
CD4 cell count, mean (SD), cells/mm^3^	413 (324)	386 (222)	378 (235)	388 (234)	391 (257)
HIV viral load suppressed (<300 copies/mL)	58 (58.0)	55 (55.6)	58 (58.0)	57 (56.4)	228 (57.0)
Current antiretroviral therapy^f^	71 (71.0)	71 (71.7)	75 (75.0)	74 (73.3)	291 (72.8)
Severe alcohol use disorder^g^	47 (47.0)	52 (52.5)	48 (48.0)	48 (47.5)	195 (48.8)
HDDs in prior 30 d, mean (SD), No.	9.5 (6.1)	9.3 (5.7)	8.9 (5.0)	9.6 (6.3)	9.3 (5.8)
PEth, median (IQR), ng/mL	44 (4-130)	37 (0-135)	23 (0-104)	36 (0-187)	34 (0-136)
Penn Alcohol Craving Scale score, mean (SD)^h^	11 (7)	11 (6)	10 (6)	11 (6)	11 (6)
Cigarettes smoked in prior 7 d, mean (SD), No./d^f^	22 (8)	20 (8)	21 (7)	21 (9)	21 (8)
Pack-years, mean (SD)	27 (13)	25 (12)	25 (12)	26 (13)	26 (13)
Carbon monoxide, mean (SD), ppm	17 (9)	18 (10)	17 (9)	17 (9)	17 (9)
Cotinine, mean (SD), μg/L	217 (117)	205 (123)	205 (130)	205 (112)	208 (120)
Readiness-to-quit score, mean (SD)					
Drinking score, scale of 0-10^i^	4.8 (2.5)	4.8 (2.3)	4.8 (2.5)	4.7 (2.5)	4.8 (2.5)
Smoking score, scale of 1-10^j^	6.6 (0.8)	6.7 (0.9)	6.6 (1.0)	6.6 (1.0)	6.6 (1.0)
Current (prior 30 d) opioid use	29 (29.0)	23 (23.2)	25 (25.0)	20 (19.8)	97 (24.3)

Abbreviations: HCV, hepatitis C virus; HDD, heavy drinking day; PEth, phosphatidylethanol.

SI conversion factor: To convert cotinine to nanomoles per liter, multiply by 5.675.

^a^ Group 1 received active varenicline and placebo nicotine replacement therapy; group 2 received placebo varenicline and active nicotine replacement therapy; group 3 received active cytisine and placebo nicotine replacement therapy; group 4 received placebo cytisine and active nicotine replacement therapy.

^b^ Several variables have small amounts of missing data as follows: depressive symptoms (1 individual in group 3), anxiety symptoms (1 individual in group 1, 1 individual in group 2, and 2 individuals in group 3), HCV co-infection (1 individual in group 4), and CD4 cell count (1 individual in group 4).

^c^ Ninth grade or higher.

^d^ Based on Center for Epidemiologic Studies-Depression score of 16 or greater.

^e^ Based on General Anxiety Disorder-7 score of 10 or greater, corresponding to moderate or severe anxiety.

^f^ Stratification factor.

^g^ Presence of 6 or more symptoms on the *Diagnostic and Statistical Manual of Mental Disorders* (Fifth Edition).

^h^ Scores range from 0 to 30, with higher scores indicating greater craving. Scores of more than 20 indicate a positive alcohol craving symptom.

^i^ Scores range from 0 to 10, with higher scores indicating greater readiness to quit drinking.

^j^ Scores range from 1 to 10, with higher scores indicating greater readiness to quit smoking.

In follow up, 348 randomized participants (87.0%) were assessed at 3 months, 343 participants at 6 months (85.8%), and 326 participants (81.5%) at 12 months (Figure 2). During the study, 16 participants withdrew consent and 10 participants were incarcerated.

Unadjusted results are shown in Table 2 and adjusted results are shown in Table 3. At 3 months, the mean (SD) number of HDDs in the prior 30 days was 2.0 (3.8) HDDs for group 1, 2.1 (4.3) HDDs for group 2, 1.5 (3.3) HDDs for group 3, and 2.4 (5.2) HDDs for group 4 (Table 3). These were decreased compared with baseline values (group 1: 9.5 [6.1] HDDs; group 2: 9.3 [5.7] HDDs; group 3: 8.9 [5.0] HDDs; group 4: 9.6 [6.3] HDDs) (Table 1). Participants randomized to varenicline (2.0 [3.8] HDDs vs 2.1 [4.3] HDDs; IRR, 0.94; 95% CI, 0.49-1.79; *P* = .85) and cytisine (1.5 [3.3] HDDs vs 2.4 [5.2] HDDs; IRR, 0.60; 95% CI, 0.30-1.18; *P* = .42) did not have significantly different numbers of HDDs in the prior 30 days compared with those randomized to NRT (Table 3). There was no significant difference in the number of HDDs in the prior 30 days comparing participants randomized to varenicline with those randomized to cytisine (2.0 [3.8] HDDs vs 1.5 [3.3] HDDs; IRR, 1.29; 95% CI, 0.65-2.55; *P* = .85) (Table 3). Results were similar in exploratory analyses comparing participants receiving combined partial agonists (varenicline and cytisine) with those receiving NRT (IRR, 0.75; 95% CI, 0.47-1.21; *P* = .24).

**Table 2.** Unadjusted Results

Outcome	Varenicline group^a^		Cytisine group^a^	
	Group 1 (n = 100)	Group 2 (n = 99)	Group 3 (n = 100)	Group 4 (n = 101)
**Baseline**				
HDDs in prior 30 d, No.				
Mean (SD)	9.5 (6.1)	9.3 (5.7)	8.9 (5)	9.6 (6.3)
Median (IQR)	7 (5-11)	8 (6-10)	8 (6-10)	7 (6-10)
Cigarettes smoked in prior 7 d, No./d				
Mean (SD)	21.7 (8.2)	20.4 (8.2)	20.8 (7.3)	20.9 (8.6)
Median (IQR)	20 (17.9-27.1)	20 (15-22.9)	20 (17.5-22.1)	20 (15-25)
**3 mo**				
HDDs in prior 30 d, No.	89	88	83	88
Mean (SD)	1.9 (3.6)	2.0 (4.2)	1.4 (3.3)	2.3 (5.3)
Median (IQR)	0 (0-3)	0 (0-2)	0 (0-1)	0 (0-2)
Cigarettes smoked in prior 7 d, No./d				
Mean (SD)	4.8 (6.8)	6 (7.2)	6.3 (7.5)	6 (7.2)
Median (IQR)	1.9 (0-6)	3.2 (0.8-9.1)	2.6 (0-10)	4.4 (0-9.7)
Abstinence				
Smoking, No. (%)^b^	22 (24.7)	12 (13.6)	23 (27.7)	20 (23)
Alcohol^c^				
No.	89	88	80	83
No. (%)	22 (24.7)	18 (20.5)	18 (22.5)	17 (20.5)
**6 mo**				
HDDs in prior 30 d, No.	86	88	83	86
Mean (SD)	1.7 (3.8)	1.0 (2.2)	1.4 (3.4)	2.1 (4.9)
Median (IQR)	0 (0-2)	0 (0-1)	0 (0-1)	0 (0-1)
Cigarettes smoked in prior 7 d, No./d				
Mean (SD)	7.5 (6.5)	7.3 (7.9)	6.7 (7)	7.9 (8)
Median (IQR)	7.1 (0.4-10)	5 (0.4-10.4)	5 (0-10)	5 (0.1-14.6)
Smoking abstinence, No. (%)	12 (14.1)	14 (16.3)	17 (20.7)	17 (20.2)
**12 mo**				
HDDs in prior 30 d, No.	79	82	80	85
Mean (SD)	2.4 (5.7)	1.6 (3.9)	2.0 (5.3)	3.0 (6.7)
Median (IQR)	0 (0-2)	0 (0-1)	0 (0-1)	0 (0-1)
Cigarettes smoked in prior 7 d, No./d				
Mean (SD)	8.4 (6.7)	7.8 (8.1)	7.2 (7.5)	8.2 (8)
Median (IQR)	8.6 (2.1-11.4)	5.4 (0.6-15)	5 (0-11.1)	7 (0.4-13.7)
Smoking abstinence, No. (%)	14 (17.7)	15 (18.3)	20 (25.3)	17 (20.2)

Abbreviation: HDD, heavy drinking day.

^a^ Group 1 received active varenicline and placebo nicotine replacement therapy; group 2 received placebo varenicline and active nicotine replacement therapy; group 3 received active cytisine and placebo nicotine replacement therapy; group 4 received placebo cytisine and active nicotine replacement therapy.

^b^ Smoking abstinence was defined as self-reported 7-day point prevalence abstinence that was biochemically validated using a threshold of a carbon monoxide level of less than 10 ppm.

^c^ Alcohol abstinence was defined as self-report of no alcohol use in the prior 30 days and a phosphatidylethanol level of less than 8 ng/mL.

**Table 3.** Effects on Alcohol and Smoking

Outcome	Group 1 vs group 2^a^		Group 3 vs group 4^a^		Group 1 vs group 3^a^	
	Outcome (95% CI)	*P* value	Outcome (95% CI)	*P* value	Outcome (95% CI)	*P* value
**No. of HDDs in prior 30 d, IRR**						
3 mo (primary outcome)^b^	0.94 (0.49-1.79)	.85	0.60 (0.30-1.18)	.42	1.29 (0.65-2.55)	.85
6 mo^c^	1.27 (0.61-2.64)	.76	0.61 (0.28-1.33)	.65	1.12 (0.53-2.36)	.76
12 mo^d^	1.27 (0.55-2.95)	.78	0.76 (0.34-1.70)	.78	1.13 (0.49-2.60)	.78
**Abstinence, OR**						
Alcohol at 3 mo^e^	1.28 (0.62-2.63)	.75	1.13 (0.54-2.38)	.75	1.12 (0.54-2.33)	.75
Smoking^f^						
3 mo	2.10 (0.93-4.72)	.22	1.22 (0.60-2.46)	.89	0.95 (0.47-1.90)	.89
6 mo	0.89 (0.38-2.08)	>.99	1.00 (0.46-2.17)	>.99	0.79 (0.35-1.78)	>.99
12 mo	1.00 (0.45-2.24)	.99	1.17 (0.56-2.46)	.99	0.68 (0.32-1.45)	.97

Abbreviations: HDD, heavy drinking day; IRR, incidence rate ratio.

^a^ Group 1 received active varenicline and placebo nicotine replacement therapy; group 2 received placebo varenicline and active nicotine replacement therapy; group 3 received active cytisine and placebo nicotine replacement therapy; group 4 received placebo cytisine and active nicotine replacement therapy.

^b^ At 3 months, the mean (SD) number of HDDs for groups 1, 2, 3, and 4 was 2.0 (3.8) HDDs, 2.1 (4.3) HDDs, 1.5 (3.3) HDDs, and 2.4 (5.2) HDDs, respectively.

^c^ At 6 months, the mean (SD) number of HDDs for groups 1, 2, 3, and 4 was 1.8 (3.9) HDDs, 1.2 (2.7) HDDs, 1.6 (3.7) HDDs, and 2.3 (5.1) HDDs, respectively.

^d^ At 12 months, the mean (SD) number of HDDs for groups 1, 2, 3, and 4 was 3.3 (6.6) HDDs, 2.5 (5.4) HDDs, 2.9 (6.3) HDDs, and 3.8 (7.3) HDDs, respectively.

^e^ Alcohol abstinence was defined as self-report of no alcohol use in the prior 30 days and biochemically validated with a phosphatidylethanol level of less than 8 ng/mL.

^f^ Smoking abstinence was defined as self-reported 7-day point prevalence abstinence that was biochemically validated using a threshold of carbon monoxide level of less than 10 ppm.

For alcohol-related secondary end points, we found no significant differences in the number of HDDs for any pairwise comparisons of interest (ie, varenicline vs NRT, cytisine vs NRT, or varenicline vs cytisine) at 6 or 12 months (Table 2). Biochemically validated alcohol abstinence at 3 months was found for 84 participants (21.0%) in the overall study sample and was similar across 4 study groups (eTable 1 in Supplement 2); results were similar for self-reported alcohol abstinence at 6 and 12 months.

For smoking-related secondary end points, 6-month biochemically validated smoking abstinence ranged across the 4 groups, from 15 of 100 participants (15.0%) in group 1 to 19 of 100 participants (19.0%) in group 3 (eTable 1 in Supplement 2). At 6 months, we did not detect significant differences in biochemically validated 7-day point prevalence abstinence for any pairwise comparison of interest, including group 1 vs 2 (15.0% vs 17 of 99 individuals [17.2%]; odds ratio [OR],0.89; 95% CI, 0.38-2.08), 3 vs 4 (19.0% vs 19 of 101 individuals [18.8%]; OR, 1.00; 95% CI, 0.46-2.17), and 1 vs 3 (OR, 0.79; 95% CI, 0.35-1.78) (eTable 1 in Supplement 2; Table 3). Results were similar at 3 and 12 months (eTable 1 in Supplement 2; Table 3).

Self-reported adherence to study medication did not differ across treatment groups. Overall adherence was higher for pills (348 participants [87.0%) than for spray medications (238 participants [59.5%]) (*P* < .001) (eTable 2 in Supplement 2). In prespecified secondary per-protocol analyses including only participants adherent to study medications, there were no significant differences in percent HDDs, cigarettes smoked/d, smoking abstinence, or alcohol abstinence (eTable 3 in Supplement 2). Medications were generally well-tolerated; there were no serious adverse events related to study medication (eTable 4 in Supplement 2). Adverse events did not vary by study group (eTable 4 in Supplement 2).

In post hoc descriptive analyses, at each follow-up time, the mean (SD) number of HDDs was lower among participants who stopped smoking vs continued smoking at 3 months (0.7 [1.8] HDDs vs 2.3 [4.6] HDDs), 6 months (1.2 [3.7] HDDs vs 1.9 [4.0] HDDs), and 12 months (1.8 [4.7] HDDs vs 3.4 [6.8] HDDs) (eTable 5 in Supplement 2). Similarly, 30-day abstinence from alcohol was greater among participants who stopped smoking at 3 months (biochemically validated with PEth: 30 of 85 participants [35.3%] vs 54 of 315 participants [17.1%]), 6 months (self-reported: 38 of 70 participants [54.3%] vs 97 of 330 participants [29.4%]), and 12 months (self-reported: 37 of 79 participants [46.8%] vs 101 of 321 participants [31.5%]) (eTable 5 in Supplement 2).

## Discussion

In St PETER HIV, a randomized clinical trial in which all participants had HIV and engaged in risky drinking and daily smoking and received an active medication and a placebo, we did not detect differences among varenicline, cytisine, and NRT on alcohol consumption or smoking outcomes. All randomized groups experienced decreases in number of HDDs at 3 months (primary study end point) and secondary end points for alcohol and smoking at all time points. St PETER HIV is, to our knowledge, the largest study to examine nicotinic acetylcholine receptor partial agonist therapy to reduce alcohol consumption and is the first to examine cytisine for this outcome.

Our work confirms and extends prior literature on varenicline for alcohol use. Meta-analyses support the associations of this nicotinic receptor partial agonist with decreased alcohol craving^9^ and consumption^8^ but comprise trials that were relatively small, did not include biochemically validated alcohol consumption end points, and did not use an active medication control treatment group or focus on individuals with HIV. While our study did not find that nicotinic receptor partial agonist therapy was more efficacious than NRT in reducing alcohol consumption, it is notable that most participants in all treatment groups reduced number of HDDs at all time points and that 21.0% of participants achieved biochemically validated alcohol abstinence at 3 months.

Our results, in combination with prior evidence, suggest several possible mechanisms by which nicotinic receptor partial and full agonists may reduce alcohol consumption. First, effects may be mediated via concomitant smoking reduction or cessation, which favorably alters biological, behavioral, and social factors that perpetuate co-use of tobacco and alcohol.^43,44,45,46^ A Cochrane review^47^ of tobacco interventions for people in treatment or recovery from alcohol suggested that concomitantly addressing smoking was not associated with increased risk for relapse to alcohol. Evidence from Project Match suggested that among treatment-seeking individuals who drink, quitting smoking was associated with a greater degree of reduced alcohol consumption compared with those who continued smoking.^48,49^ In contrast, the International Tobacco Control Four Country Survey,^50^ which did not incorporate treatment of alcohol or smoking, did not find an association between smoking cessation and reduced alcohol consumption. In post-hoc analyses of our study, we observed that individuals who quit smoking reduced their number of HDDs and quit drinking alcohol to a greater degree than individuals who continued smoking. Further study is warranted to confirm this relationship and understand the potential role of smoking cessation in reducing alcohol consumption.

Second, direct nicotinic receptor–mediated decrease in alcohol consumption has been observed in preclinical and human laboratory studies. In rodents, this process requires stimulation of nicotinic acetylcholine receptors containing the α4 subunit.^16^ Notably, varenicline and cytisine are highly specific to α4β2-type nicotinic receptors, which are also agonized by NRT. Thus, all 3 study medications could reduce alcohol consumption through this pathway, independent of their effects on smoking behavior. Nicotinic receptor–mediated decrease in alcohol consumption may seem counterintuitive given the coprevalence of smoking and risky drinking. However, while medicinal nicotine and cigarette smoking both deliver nicotine, they differ in key respects. Cigarette smoke contains more than 7000 chemicals; in contrast, medicinal nicotine contains pharmaceutical-grade nicotine and does not feature the same learned cues that prompt co-use with alcohol. There is no large-scale randomized clinical trial of NRT to reduce alcohol consumption, to our knowledge. Third, an unknown environmental factor or participation in a randomized clinical trial theoretically could have been responsible for reducing alcohol consumption, independent of smoking abstinence observed with use of these medications.

While large trials have demonstrated the efficacy of varenicline, NRT, and cytisine individually for smoking cessation, no comparison has examined these 3 medications head to head, to our knowledge. Among populations of individuals with HIV, 2 trials^6,7^ examined varenicline for smoking cessation, none examined cytisine, and none were comparative effectiveness studies. These trials reported that varenicline was safe and efficacious for smoking cessation compared with placebo. In the general population of smokers, the EAGLES trial^51^ reported varenicline to be superior to NRT monotherapy for smoking cessation among participants with and without preexisting mental health conditions, while a smaller trial^52^ found no differences in efficacy among varenicline, NRT monotherapy, and combination NRT. Similar to a past report,^14^ we found that varenicline and cytisine had similar efficacy for smoking cessation. In contrast to a published study that found cytisine to be more effective than NRT for smoking cessation,^12^ we found that cytisine and NRT had similar efficacy for this outcome.

Our results are important to individuals with HIV, their clinicians, and the larger general population. Most individuals with HIV and others who smoke and engage in risky drinking do not receive treatment for these conditions.^53^ Despite myriad reasons for lack of treatment, our results suggest that clinicians should dispel the notions that individuals with HIV and potentially others with multimorbidity cannot or will not use these medications and that if used, these medications will not be successful for smoking cessation. Importantly, our results do not support the use of nicotinic acetylcholine receptor partial agonists solely to decrease alcohol consumption. However, our findings, combined with prior large, randomized clinical trials using placebo controls that demonstrated the efficacy of these medications for smoking cessation, provide strong evidence that these medications may be used safely and effectively for smoking cessation among individuals with HIV who have multimorbidity and a history of substance use or active substance use disorders. Furthermore, given that risky drinking is common among individuals with HIV who smoke, our results suggest that the need for clinicians to treat such patients should be reinforced. In addition to the benefits of smoking cessation, it is possible that this treatment could result in decreased alcohol consumption, such as in the collateral benefit that has been described from the use of antidepressants among people with depression who also have alcohol use disorder.^54^

### Limitations

This trial has several limitations that warrant discussion. First, it was conducted at a single site and included only White men and women in St Petersburg, Russia, living with HIV infection and who had a high prevalence of comorbidities, potentially limiting generalizability to a healthier population with only risky drinking. Second, for ethical reasons, this trial was designed with a proven smoking cessation medication (as opposed to placebo control) in all treatment groups. Thus, we cannot know definitively if these drugs reduced alcohol consumption in this population. To know definitively if varenicline, cytisine, or even NRT reduced alcohol consumption independent of reduction or cessation of cigarette smoking would require a larger, placebo-controlled trial of individuals who engage in risky alcohol drinking and do not smoke. Most placebo-controlled trials of varenicline for alcohol use have been conducted in individuals who smoke; in our study and in other published studies, the sample size of individuals who did not smoke was too small to address this specific question.^9^ Additionally, we anticipated larger effect sizes in the design phase of this trial; thus, the study may have been underpowered to detect differences of the observed magnitude.

## Conclusions

This randomized clinical trial did not detect differences among varenicline, cytisine, and NRT for alcohol consumption or smoking outcomes in St PETER HIV. Regardless of treatment group, participants experienced decreases in number of HDDs and biochemically validated alcohol and smoking abstinence. Our results do not support using nicotinic acetylcholine receptor partial agonists solely to reduce alcohol use among individuals with HIV. For smoking cessation, we did not detect differences between these medications and NRT; however, the observed rates of smoking cessation were consistent with those of previous trials. For individuals who currently drink and smoke, concomitant decreases in alcohol consumption may be an additional benefit. Our findings suggest that future studies should evaluate whether concomitant decrease in alcohol consumption is a specific benefit for individuals who quit smoking.

## Notes

Supplement 1.
Trial Protocol

Supplement 2 **eFigure 1.** Schedule for Study Medications **eTable 1.** Imputed Rates of Alcohol and Smoking Abstinence **eTable 2.** Self-Reported Adherence to Study Medications by Treatment Group and Medication Type **eTable 3.** Per-Protocol Analysis of Heavy Drinking Days and Smoking Outcomes Among Participants Who Were Adherent to Study Medication **eTable 4.** Adverse Events **eTable 5.** Post Hoc Analyses Examining No. of Heavy Drinking Days and Percent With Alcohol Abstinence by Smoking Status at 3, 6, and 12 mo

Supplement 3.
Data Sharing Statement
