# Supplementary material for: Effectiveness of Varenicline and Cytisine for Alcohol Use Reduction Among People With HIV and Substance Use: A Randomized Clinical Trial
Source: JAMA Netw Open. 2022 Aug 5;5(8):e2225129. doi: 10.1001/jamanetworkopen.2022.25129 (PMC9356316; doi:10.1001/jamanetworkopen.2022.25129)
Supplement: Supplement 1. — Trial Protocol [file jamanetwopen-e2225129-s001.pdf]

Studying Partial agonists for Ethanol and Tobacco Elimination in Russians with HIV (St PETER HIV)

Principal Investigators:

Jeffrey H. Samet, MD, MA, MPH – Boston Medical Center (contact)

Matthew S. Freiberg, MD, MSc - Vanderbilt University Medical Center

Hilary A. Tindle, MD, MPH – Vanderbilt University Medical Center

Study Statistician: Debbie M. Cheng, ScD – Boston University School of Public Health

NIAAA Award Number: U01AA020780

Clinicaltrials.gov Registration: NCT02797587

Boston University Medical Campus IRB Protocol Number: H-35288

Enrollment Dates: July 19, 2017 – December 25, 2019

Statistical Analysis Plan (SAP) Version Date: 03/27/2020

SAP Revision History

Date of revision	Section number changed	Description and reason for change
12/17/2020	5.1 Outcome Definitions	Adding PEth
12/17/2020	3.2 Adherence and Protocol Deviations	More detail added around definitions of adherence
12/17/2020	5.4 Additional Analyses	Alcohol cessation using PEth and self-report
1/29/2021	3.2 Adherence and Protocol Deviations	Additional detail around definitions of adherence
2/3/2021	5.4 Additional Analyses	Added smoking cessation definition for participants who self-report quitting smoking, but endorse cannabis use at the 3-month visit and failed their CO test

SECTION 1: INTRODUCTION

1.1 STUDY HYPOTHESES

We hypothesize that:

Hypothesis 1- Varenicline will have greater effects than NRT for reducing alcohol consumption, smoking, inflammation, CHD, and mortality risk;

Hypothesis 2- Cytisine will have greater effects than NRT for these outcomes; and

Hypothesis 3- Varenicline will have greater effects than cytisine for these outcomes.

SECTION 2: STUDY METHODS

2.1 STUDY DESIGN

St PETER is a Randomized Controlled Trial (RCT) among 400 HIV+ persons with heavy alcohol consumption (by NIAAA definition) who smoke, which aims to compare effects of varenicline, cytisine, and NRT on reduction of: 1) alcohol use and craving, 2) smoking, and 3) inflammation and risk for CHD and mortality. Self-reported alcohol and smoking outcomes will be assessed at 1, 3 (primary), 6, and 12 months. Eligible participants will be randomly assigned into one of four study arms: 1) Varenicline + NRT placebo; 2) Varenicline placebo + NRT; 3) Cytisine + NRT placebo; 4) Cytisine placebo + NRT. All participants will receive evidence-based counseling for alcohol and tobacco use, 1 active medication, and 1 placebo. Participants in all study arms will receive brief evidence-based counseling for alcohol and tobacco use at baseline.

Throughout the course of the study, participants will be expected to come in for five in-person assessments and blood draw visits (baseline, 1-, 3-, 6-, and 12-months); additionally, participants will have two in-person check ins at weeks two and eight (2-months), and four check-ins via phone (weeks 1, 3, 6, 10).

2.2 Randomization

See section 3.2 of Study Protocol

2.3 Sample size

See section 2.8.A. of Study Protocol

2.4 framework

St PETER is a superiority trial that will test the following 3 comparisons:

Hypothesis 1- Varenicline will have greater effects than NRT for reducing alcohol consumption, smoking, inflammation, CHD, and mortality risk;

Hypothesis 2- Cytisine will have greater effects than NRT for these outcomes; and

Hypothesis 3- Varenicline will have greater effects than cytisine for these outcomes.

We will test each of these 3 pairwise comparisons and adjust for multiple testing within each outcome using the Hochberg sequential test procedure.

2.5 Statistical interim analyses and stopping guidance

No interim efficacy analyses are planned. An external Data and Safety Monitoring Board will monitor safety.

2.6 Timing of final analyses

We will analyze primary and secondary 3- and 6-month study outcomes once all 6-month outcome data have been collected and cleaned. The study will remain blinded to assessors, until all secondary/exploratory 12-month data have been collected.

2.7 Timing of outcome assessments

See section 3.5A of Study Protocol

The schedule of study procedures is provided in Section 3.5 of the Study Protocol. The expected visit dates and visit windows are defined in Section 3.5A of the Study Protocol. All follow-up visit dates are calculated based off the date of the participant's baseline visit.

Section 3: Statistical Principles

3.1 Confidence intervals and P values

Statistical tests will be 2-sided and will be performed using an overall 5% significance test. Within each outcome, we will adjust for the multiple comparisons due to the 3 pairwise comparisons using the Hochberg sequential test procedure. Confidence intervals will be reported for measures of effect.

3.2 Adherence and protocol deviations

Pills

Participants will be considered adherent to study medication if they self-report taking at least 80% of their assigned study medication on the VAS in the past week as follows:

Active cytisine: Reporting >80% adherence at 2 of 3 study visits prior to the 1 month visit

Active varenlicline: Reporting >80% adherence at 4 of 7 study visits prior to the 3-month visit

Spray

Weeks 1-4 of treatment (study assessments are 1 week, 2 weeks, and 3 weeks): Participants will be considered adherent if they report using at least 8 sprays per day at least 80% of the days in the last week (VAS) in at least 2 of 3 assessments.

Weeks 5-8 of treatment (study assessments at 1 month and 6 weeks): Participants are considered adherent if they report no urge to smoke or if they report using the spray at least 80% of times when having an urge to smoke in the last week (VAS) in at least 1 of 2 visits prior to the 2 month visit.

3.3 Analysis Populations

The intention-to-treat (ITT) population will include all randomized participants according to the study group assigned.

The per-protocol population will include all participants meeting the definition of adherence noted in section 3.2 above.

SECTION 4: TRIAL POPULATION

4.1 Screening Data

The following data will be provided for all screened participants: number of patients assessed for eligibility, number of participants enrolled, reasons for ineligibility and non-enrollment.

4.2 Eligibility

See section 2.6 and 2.7 of Study Protocol.

4.3 Recruitment

The CONSORT diagram will present data on number of participants screened, eligible, enrolled, randomized, assigned to each study arm, and completing follow up.

4.4 Withdrawal/follow up

Reasons for discontinued treatment will be presented in the CONSORT diagram/trial profile.

4.5 Baseline Participant Characteristics

Descriptive statistics will be calculated for the following variables at baseline overall and stratified by: randomized group: age, sex, heavy alcohol use (defined as ≥ 3 vs. < 3 heavy drinking days in the past week according to NIAAA risky drinking criteria, assessed via the first 7 days of the Timeline Follow Back)*, average daily cigarettes (≤ 1 vs. >1 pack per day, assessed via question 5 of the Tobacco Use section)*, current ART use [yes vs. no]*, percent heavy drinking days in the past month, education, marital status, employment, BMI, depressive symptoms, HCV status, CD4, HIV viral load, alcohol use disorder category, cigarettes smoked per day in the past week, recent quit attempts (smoking), pack years, readiness to quit drinking and smoking, current marijuana use, lifetime opioid use, and current opioid use. For continuous variables, the following will be provided: median, mean, standard deviation, 0th, 25th, 50th, 75th, and 100th percentiles. For categorical variables, frequencies and proportions will be provided. For each primary and secondary outcome, descriptives will also be reported stratified by arm and follow-up time, (note, no testing will be done for any of the above).

*stratification factor

SECTION 5: ANALYSIS

5.1 Outcome Definitions

**Primary (study)**	Count of heavy drinking days (HDD) in the past month measured at 3 months using self-reported past 30 day alcohol consumption obtained via the Timeline Follow Back (TLFB) method				
**Primary (smoking)**	Self-reported cigarettes per day in the past week measured at 3 months				
**Secondary**	Alcohol craving (Penn Alcohol Craving Score)				
**Secondary**	Count of HDD in the past month measured at 1 month				
**Secondary**	Count of HDD in the past month measured at 6 months				
**Secondary**	Count of HDD in the past month measured at 12 months				
**Secondary**	Biochemically				

	confirmed 7-day point prevalence abstinence from smoking at 3 months				
**Secondary**	Biochemically confirmed 7-day point prevalence abstinence from smoking at 6 months				
**Secondary**	Biochemically confirmed 7-day point prevalence abstinence from smoking at 12 months				
**Secondary (Aim 3)**	Biomarkers of inflammation (hsCRP and IL-6), Reynolds Risk Score and VACS Index Score at 3 months				
**Post hoc exploratory outcome**	Change in log PEth from baseline to 3 months				
**Post hoc exploratory outcome**	Alcohol abstinence				

See section 2.1 of Study Protocol.

5.2 Analysis Methods

The main analysis evaluating the impact of each intervention on the primary study outcome (i.e., the continuous variable % HDD at 3 months) will use multiple regression models. The models will include indicator variables to represent study arm. We will test 3 pairwise comparisons (i.e., varenicline + NRT placebo vs. NRT + varenicline placebo; cytisine + NRT placebo vs. NRT + cytisine placebo; varenicline vs. cytisine) and will adjust for the multiple comparisons using the Hochberg sequential test procedure. To improve efficiency, the regression analyses will control for stratification factors: alcohol consumption (≥ 3 vs. < 3 heavy drinking days in the past week according to NIAAA risky drinking criteria, assessed via the first 7 days of the Timeline Follow Back), average daily cigarettes (≤ 1 vs. >1 pack per day, assessed via question 5 of the Tobacco Use section), and current ART use [yes vs. no]. If the data are normally distributed, multiple linear regression models will be used. However, if the distribution of % HDD is skewed, transformations of the data will be performed (e.g., log transformation). If outcomes are transformed to count data (e.g., number of HDD, number of cigarettes) that are not approximately normally distributed, they will be analyzed using negative binomial (to account for overdispersion) regression models. Continuous outcomes (e.g., biomarkers of inflammation, Reynold's risk score, VACS Index, primary and secondary outcomes for Aim 3) will be analyzed using the same approach described above. Binary outcomes (e.g., smoking cessation, secondary outcome Aim 2) will be analyzed using

logistic regression models. Models will control for stratification factors as described above for % HDD. A secondary analysis will be conducted using a per protocol approach that includes only those participants who were adherent to their assigned intervention (i.e., taking study medications or placebos 80% of the time).

5.3 Missing Data

Participants who meet eligibility criteria and agree to participate will be compared with participants who were determined to be eligible but declined enrollment on data captured during eligibility assessment. The two independent samples t-test and Fisher's exact test will be used to test for statistically significant differences between participants who enroll and those eligible who do not, and to test for significant differences between participants lost to follow-up and those who complete it. Missing data patterns will be evaluated including the frequency and percentage of participants missing for each variable and the distribution of the number of variables missing for participants. In addition, data collected to the point of loss to follow-up will be compared to the data of those who complete the study to examine missing data mechanisms. In situations where missing data occurs, we will document the reasons for the missing data whenever possible. The proposed study has accounted for a 20% random non-informative loss to follow-up and will still have sufficient power with this potential loss in size. While data may not be missing completely at random, it may be reasonable to assume that data are missing at random.

5.4 Additional Analyses

Intervention effects over time

The main analyses will focus on % HDD at 3months, our primary outcome. Primary analyses of smoking and inflammation are also assessed at 3 months. However, given the repeated measures of our alcohol and smoking variables (i.e., 1, 3, 6, and 12 months), additional analyses using longitudinal regression models will be used to incorporate repeated measures for each outcome (alcohol and smoking) in the same model and will test for possible intervention by time interactions (e.g., does the effect of varenicline or cytisine change over time). For continuous outcomes (e.g., % HDD), we will use generalized linear mixed effects models that include subject-specific random intercepts and slopes to account for the correlation due to having repeated observations from each subject. For dichotomous outcomes, we will use generalized estimating equations (GEE), with an independence working correlation matrix, a logit link; standard errors will be based on the empirical-sandwich estimator. Secondary analyses will also be performed using mixed effects logistic regression models for correlated binary outcomes. The GEE approach compares population averages over time and the mixed effects approach models subject-specific effects; the latter is computationally more intensive and requires more modeling assumptions but may be more powerful if the assumptions are correct.

Effect Modification

Because heavy alcohol use, depressive symptoms and opioid use are common in Russia ARCH we will perform additional analyses to explore whether baseline alcohol consumption (≥15 HDD in past month), depressive symptoms (measured by CES-D score ≥16) or opioid use (self-reported history of opioid use in Russia ARCH assessment) are potential effect modifiers of the interventions. We will fit separate models that include 2-way interactions between randomization group and each potential effect

modifier. If an interaction is significant, subsequent stratified analyses will be conducted to evaluate the effect of varenicline/cytisine by alcohol, depressive symptoms, or opioid group. Exploratory analyses will also examine intervention effects by sex to describe and estimate effects within each subgroup.

Mediation

We will explore potential mechanisms through which the interventions are mediated. For example, the interventions may lead to a reduction in alcohol use, which in turn leads to reduced inflammation, CHD risk, and mortality risk. To assess potential mechanisms, we will use the approach described by Baron and Kenny. To assess whether alcohol use (% HDD) is in the causal pathway between the intervention and biomarkers of inflammation (hsCRP and IL-6), we will evaluate whether the following conditions hold: a) alcohol use is related to inflammation; b) the intervention is related to alcohol use; c) the intervention is related to biomarkers of inflammation; and d) The effect of the interventions on inflammation reduces appreciably when alcohol use is added to the model. We will conduct confirmatory analyses using the counterfactual framework, an approach that allows potential interactions between the interventions and mediators and derives direct and indirect effects with different types of outcomes (e.g., dichotomous). In addition to alcohol, we will also assess whether smoking is in the causal pathway between the interventions and markers of inflammation, CHD risk, and mortality risk.

We will also look at alcohol cessation using a combined variable of self-report and PEth. Participants will be considered abstinent if PEth<8 ng/mL and they report no alcohol use in the past 30 days.

For participants who self-report quitting smoking and using cannabis at the 3-month study visit and failed their CO test (>10 ppm), we will use urine cotinine < 50 mg to determine smoking cessation.

5.5 Harms

The number of participants experiencing each AE/SAE will be presented for each treatment arm and categorized by severity and organ system. No formal statistical testing will be undertaken.

Causes of death will be presented by study arm and time to death will be analyzed using the log-rank test. Cox proportional hazards models adjusted for stratification factors will be used to estimate hazard ratios and 95% confidence intervals.

5.6 Statistical Software

Data will be analyzed with SAS version 9.4 (SAS Institute, Inc., Cary, NC).

Studying Partial agonists for Ethanol and Tobacco Elimination in Russians with HIV (St PETER HIV)

NIAAA Award Number: U01AA020780

Clinicaltrials.gov Registration: NCT02797587

Protocol Version: January 6, 2021

Enrollment Dates: July 19, 2017- December 25, 2019

TABLE OF CONTENTS

1. INTRODUCTION #5

1.1 SUMMARY…… #6

1.2 SIGNIFICANCE…… #6

2. OVERVIEW OF STUDY DESIGN #6

2.1 STUDY AIMS…… #6

2.2 STUDY HYPOTHESES…… #6

2.3 STUDY OUTCOMES…… #6

2.4 STUDY DESIGN…… #7

2.5 STUDY SITE…… #8

2.6 INCLUSION CRITERIA…… #8

2.7 EXCLUSION CRITERIA AT STUDY ENTRY…… #9

2.8 RECRUITMENT GOALS…… #10

2.8.A. SAMPLE SIZE CALCULATION AND POWER…… #10

3. INTERVENTION #11

3.1 INTERVENTION OVERVIEW…… #11

3.2 RANDOMIZATION…… #12

3.3 INTERVENTION…… #12

3.3.A. VARENICLINE + NRT PLACEBO…… #13

3.3.B. VARENICLINE PLACEBO + NRT…… #14

3.3.C. CYTISINE + NRT PLACEBO…… #16

3.3.D. CYTISINE PLACEBO + NRT…… #17

3.3.E. BEHAVIORAL COUNSELING…… #19

3.4 MEDICATION CONSIDERATIONS…… #21

3.4.A. SYMPTOM MONITORING…… #21

3.4.B. ADHERENCE…… #21

3.4.C. MEDICATION DISBURSEMENT…… #22

3.4.D. LOST OR STOLEN STUDY MEDICATION…… #23

3.4.E. DISCONTINUATION OF STUDY MEDICATION …… #23

3.5 SCHEDULE OF DATA COLLECTION…… **#24**

3.5.A. VISIT WINDOWS…… #25

3.6 DATA SOURCES…… **#26**

3.6.A QUESTIONNAIRES…… #27

3.6.B. BLOOD …… #27

3.6.C. URINE…… #30

3.6.D. CARBON MONOXIDE…… #31

3.6.E. BAC…… #31

3.6. F. DRIED BLOOD SPOTS…… #31

4. OBSERVATIONAL COHORT **#31**

5. STUDY PROCEDURES **#31**

5.1 RECRUITMENT…… **#31**

5.2 SCREENING…… **#32**

5.3 INFORMED CONSENT…… **#32**

5.3.A. OBSERVATIONAL COHORT…… #33

5.4 VISIT FLOW…… **#33**

5.5 QUALITY ASSURANCE…… **#34**

5.6 COMPENSATION…… **#35**

5.7 RETENTION…… **#35**

6. ASSESSMENTS **#33**

6.1 BASELINE ASSESSMENT…… **#37**

6.1.A. OBSERVATIONAL COHORT BASELINE ASSESSMENT…… #38

6.2 FOLLOW UP ASSESSMENTS…… **#38**

6.2.A. MEDICATION VISITS ASSESSMENTS…… #39

6.2.B. OBSERVATIONAL COHORT ASSESSMENTS……………………………………….….……......#39

7. PARTICIPANT SAFETY #39

7.1 SPECIFICATION OF SAFETY PARAMETERS…………………………………………………**#39**

7.2 THE METHODS AND TIME FOR ASSESSING, RECORDING, AND ANALYZING SAFETY PARAMETERS…………………………………………………......................................**#40**

7.3 THE METHODS AND TIME FOR ASSESSING, RECORDING, AND ANALYZING SAFETY PARAMETERS AND INTERCURRENT ILLNESSES…………………………………………….#**43**

7.3.A. OTHER EVENTS………………………………………………………….……….........**#44**

7.3.B. PARTICIPANT ADVICE…………………………..…………………….……….........**#45**

7.3.C. ADVERSE EVENT REPORTING……………………….………...**#45**

7.3.D. SAE REPORTING……………………….………...**#46**

7.4 THE TYPE AND DURATION OF THE FOLLOW-UP OF SUBJECTS AFTER ADVERSE EVENTS…………………………………………………………………………………..**#48**

7.5 UNBLINDING PROTOCOL…………………………………………………………...…**#48**

7.6 DATA SAFETY AND MONITORING BOARD……………………………………….……**#49**

8. DATA MANAGEMENT #50

8.1 DATA COLLECTION………………………………………………………………….........**#50**

8.2 QUALITY CONTROL PROCESS.. **#50**

8.3 DATA SECURITY AND CONFIDENTIALITY……………………………………………......**#51**

8.4 WEB SYSTEMS………………………………………………………………………......**#51**

9. STATISTICAL ANALYSIS #52

9.1 PRIMARY ANALYSES……………………………………………………………….……......**#53**

9.2 ADDITIONAL EXPLORATORY ANALYSES….…………………………………….…......**#53**

10. STAFF TRAINING #54

1. INTRODUCTION

1.1 SUMMARY

HIV infected (HIV+) heavy drinking smokers are at high risk for coronary heart disease (CHD) and death.[1] The mechanisms driving increased CHD risk in HIV+ people are unclear, but are linked to inflammation.[2,3,4,5] HIV, heavy drinking, and smoking are all pro-inflammatory.[6,7] HIV viral suppression with antiretroviral therapy does not eliminate the elevated CHD risk nor the increased inflammation (i.e., pre-HIV infection levels are not restored).[1] Interventions that reduce alcohol use, smoking, or both in HIV+ people could lower inflammation, CHD and death risk.[8,9] Varenicline and cytisine are proven therapies for smoking cessation.[10,11-13] When compared to placebo, varenicline has higher cessation rates than cytisine.[11,14] Human trials suggest varenicline also has efficacy for reducing alcohol consumption and craving in heavy drinking smokers.[15-20,20] In murine models, cytisine reduces alcohol consumption.[21-23] The comparative efficacy of varenicline and cytisine to reduce alcohol consumption and by extension, inflammation, CHD, and mortality risk, in humans has not been tested, nor has their comparative effectiveness been tested for smoking. Neither drug has been tested for smoking cessation against nicotine replacement therapy (NRT) in HIV+ heavy drinking smokers.

Three compelling reasons to test varenicline and cytisine in HIV+ heavy drinking smokers are: 1) both show promise in HIV-uninfected people; 2) the morbidity caused by heavy drinking and smoking in HIV+ persons is significant; and 3) treating heavy drinking and smoking with one medication represents a significant advance in reducing polypharmacy and improving patient care. Thus, we propose a 4-arm placebo-controlled randomized controlled trial (RCT) among 400 HIV+ heavy drinking smokers. The trial arms are: varenicline + NRT placebo, cytisine + NRT placebo, NRT + varenicline placebo, and NRT + cytisine placebo. All participants will receive counseling (alcohol & tobacco) and medications (placebo & active). There are 3 pairwise comparisons of interest: i) varenicline+ NRT placebo vs. NRT + varenicline placebo; ii) cytisine+ NRT placebo vs. NRT + cytisine placebo; iii) varenicline vs. cytisine. The 4-arm design allows blinding of varenicline and cytisine without placing undue burden on study participants to take more than 1 placebo, as a 3-arm design would have required. Our specific aims will compare effects of varenicline, cytisine, and NRT at 3 months on past month % heavy drinking days (% HDD) and alcohol craving, cigarettes per day and smoking abstinence (verified by carbon monoxide), inflammation (hsCRP, IL-6), CHD (Reynolds risk score), and mortality (VACS index) risk. We hypothesize that (1) Varenicline has greater efficacy than NRT for reducing heavy drinking, smoking, inflammation, CHD and mortality risk; (2) Cytisine has greater efficacy than NRT; and (3) Varenicline has greater efficacy than cytisine for these outcomes. We will conduct an RCT, Studying Partial agonists for Ethanol and Tobacco Elimination in Russians with HIV (St PETER HIV), in a country with an HIV epidemic and high per-capita alcohol consumption and smoking. We will recruit from our ongoing Russia ARCH cohort in St. Petersburg (part of our NIAAA-funded HIV/AIDS Alcohol Consortium – URBAN ARCH). If our hypotheses are correct, St PETER HIV could make nicotinic partial-agonists standard care for HIV+ heavy drinking smokers, and lead to reduced inflammation, CHD and mortality[24] risk through this "one drug, two diseases" approach. This trial addresses the paucity of RCT data to guide treatment of these CHD risk factors in HIV+ people.

1.2 SIGNIFICANCE

The Russia ARCH Cohort and the St PETER HIV study will draw from an established cohort of HIV-infected smokers and heavy drinkers to compare the effects of two partial nicotinic receptors, varenicline and cytisine, on alcohol consumption, alcohol craving, smoking, inflammation, CHD risk, and mortality risk. St PETER HIV further addresses the paucity of randomized controlled trial data to guide treatment of heavy alcohol consumption and smoking in HIV-infected people.

2. OVERVIEW OF STUDY DESIGN

2.1 STUDY AIMS

Our Specific Aims will compare effects of varenicline, cytisine, and NRT on 3 major conditions responsible for serious morbidity and mortality among HIV+ people:

Aim 1: % Heavy drinking days in past month (self-report, primary outcome) and alcohol craving (self-report);

Aim 2: Cigarettes per day (past week, self-report); 7-day point prevalence abstinence (biochemically verified);

Aim 3: Inflammation (hsCRP, IL-6), CHD risk (Reynolds risk score), and mortality risk (VACS index).

2.2 STUDY HYPOTHESES

We hypothesize that:

1. Varenicline will have greater effects than NRT for reducing alcohol consumption, smoking, inflammation, CHD, and mortality risk;
2. Cytisine will have greater effects than NRT for these outcomes; and
3. Varenicline will have greater effects than cytisine for these outcomes.

2.3 STUDY OUTCOMES

Aim 1. The primary outcome for Aim 1, % HDD in the past month measured at 3 months, will be assessed using self-reported past 30-day alcohol consumption obtained via the Timeline Followback (TLFB) method.[25] TLFB was adapted for the Russian setting by using local metrics recording the volume (in liters) and type of spirit consumed. Calculations convert reported data into standard drinks. We will use

the Penn Alcohol Craving Score [PACS] to measure Alcohol Craving (secondary outcome, Aim[26] 1). Additional secondary outcomes include % HDD in the past month measured at 1, 6, and 12 months.

Aim 2. The primary outcome for Aim 2, self-reported cigarettes per day in the past week measured at 3 months was selected rather than cessation because prior intervention trials involving varenicline and participants who are heavy drinkers, smokers and/or who also have depressive symptoms, and/or use opioids reported significant findings more often with reductions in smoking, rather than cessation from smoking. Current cigarettes per day will be measured using the TLFB method which has been successfully employed for smoking studies and allows conversion to cigarettes per week. Because cessation is very important, we will use carbon monoxide (CO) validated smoking cessation as our secondary outcome for Aim 2. Cessation is defined as self-reported 7-day point prevalence abstinence and a CO threshold of <10 ppm, measured in end-expired air (based on guidelines for biochemical validation);[27] a recent, stricter definition of abstinence (CO <4-5 ppm) will also be assessed.[28] These variables will be captured at 1, 6, and 12 months as secondary outcomes.

Aim 3. To measure the primary outcome for Aim 3, biomarker of inflammation levels at 3 months, we selected hsCRP because these biomarkers are significantly associated with heavy alcohol consumption, smoking, incident CHD, and mortality in HIV+ people. Reynolds score and VACS index at 3 months (secondary outcomes, Aim 3) were selected because we do not have enough participants to power our intervention to CHD and mortality events, as is the case for most alcohol and tobacco trials. The Reynolds risk score assesses CHD risk, has been validated in large cohorts of men and women, and is routinely used in clinical practice.[1] The VACS index is a validated surrogate measure of mortality in HIV+ people.[29,30]

2.4 STUDY DESIGN

St PETER is a Randomized Controlled Trial (RCT) among 400 HIV+ persons with heavy alcohol consumption (by NIAAA definition) who smoke, which aims to compare effects of varenicline, cytisine, and NRT on reduction of: 1) alcohol use and craving, 2) smoking, and 3) inflammation and risk for CHD and mortality. Self-reported alcohol and smoking outcomes will be assessed at 1, 3 (primary), 6 and 12 months. Eligible participants will be randomly assigned into one of four study arms: 1) Varenicline + NRT placebo; 2) Varenicline placebo + NRT; 3) Cytisine + NRT placebo; 4) Cytisine placebo + NRT. All participants will receive evidence-based counseling for alcohol and tobacco use, 1 active medication, and 1 placebo.

Figure 1 illustrates the design of this 4-arm parallel group RCT with 100 participants per arm. The 4-arm design allows blinding of varenicline and cytisine without placing undue burden on study participants to take more than 1 placebo, as a 3-arm design would have required. This is especially important given the different well-established dosing regimens of varenicline (3 months) and cytisine (multi-daily dosing for 25 days); NRT will be administered for 8 weeks. Participants in all study arms will receive brief evidence-based counseling for alcohol and tobacco use at baseline.

Figure 1: Study Design

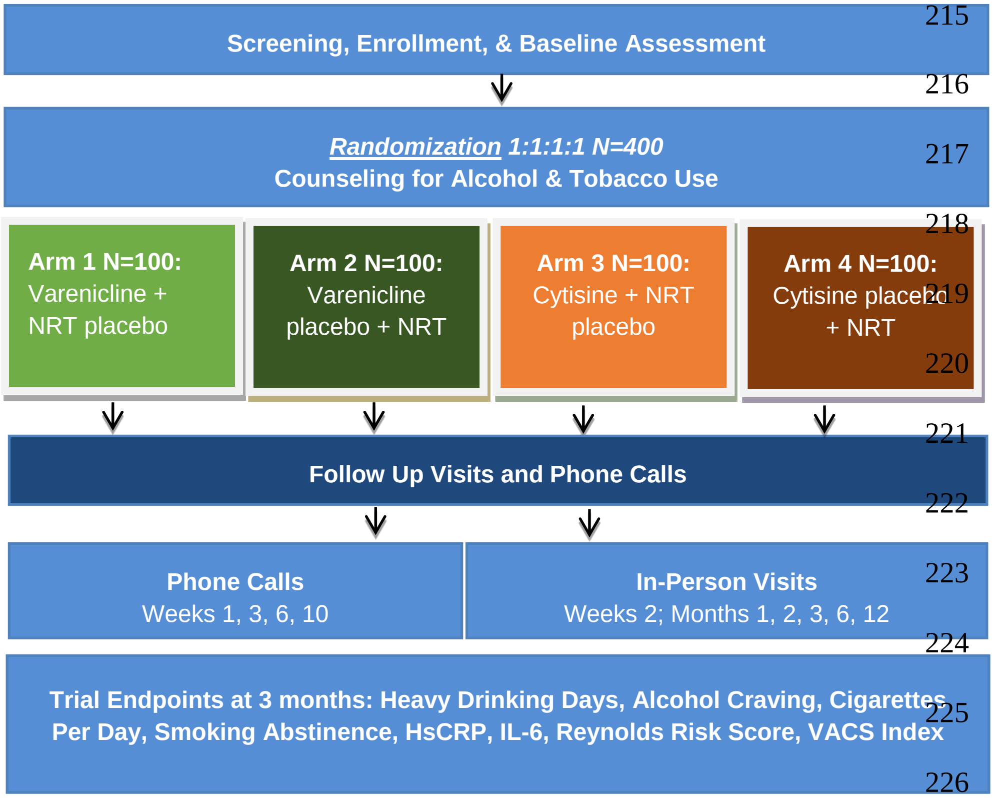

Throughout the course of the study, participants will be expected to come in for five in-person assessments and blood draw visits (baseline, 1-, 3-, 6-, and 12-months); additionally, participants will have two in-person check ins at weeks two and eight (2-months), and four check-ins via phone (weeks 1, 3, 6, 10). During these calls, participants will be monitored for adverse events.

2.5 STUDY SITE

Recruitment, enrollment, and all study visits will take place at the Laboratory of Clinical Pharmacology of Addictions at the First St. Petersburg Pavlov State Medical University (PSMU) in St. Petersburg, Russia. PSMU is the major educational, scientific, and clinical medical institution for northwestern Russia. Blood specimens will be processed and analyzed at ImmunoBioService (IBS) under the direction of Dr. Sergei Selkov.

2.6 INCLUSION CRITERIA

To be eligible to participate in the trial, participants will need to meet the following inclusion criteria:

1. 18-70 years old

2. HIV-infected
3. ≥5 heavy drinking days [i.e., NIAAA at-risk drinking levels] in the past 30 days
4. Smoking an average of at least 5 cigarettes per day
5. Provision of contact information for 2 contacts to assist with follow-up
6. Stable address within 100 kilometers of St. Petersburg
7. Possession of a telephone (home or cell)
8. Interest in cutting down alcohol or tobacco
9. Able and willing to comply with all study protocols and procedures

2.7 EXCLUSION CRITERIA AT STUDY ENTRY

1. Not fluent in Russian
2. Cognitive impairment resulting in inability to provide informed consent based on research assessor (RA) assessment
3. Pregnancy, planning to become pregnant in next 3 months, or breast feeding
4. Unstable psychiatric illness (i.e. ,answered yes to any of the following: past three month active hallucinations; mental health symptoms prompting a visit to the ED or hospital; mental health medication changes due to worsening symptoms; presence of suicidal ideations)
5. History of seizures
6. Acute coronary syndrome within 1 month of enrollment
7. Taking smoking cessation medications in the past 30 days
8. History of pheochromocytoma
9. History of Buerger's disease
10. Systolic blood pressure greater than 180 mm Hg or diastolic blood pressure greater than 105 mm Hg.
11. Currently taking anti-tuberculosis medications
12. Currently taking Galantamine or Physostigmine
13. Breath alcohol content (BAC) level of 0.10% or higher
14. Known allergy to varenicline (Chantix) or cytisine (Tabex)

2.8 RECRUITMENT GOALS

We aim to randomize 400 participants into the trial and a total of up to 500 may be enrolled, but not randomized due to not meeting study entry criteria. All existing Russia ARCH participants will be screened for St PETER HIV. Recruitment beyond PSMU will occur at local NGOs and Russia ARCH network hospitals, including the major clinical HIV hospitals (Botkin Infectious Disease Hospital, Leningrad Regional AIDS Center, St. Petersburg City AIDS Center) and addiction hospitals (Leningrad Regional Center for Addictions, St. Petersburg City Addiction Hospital) and through snowball recruitment; recruitment will occur over 30 months (~13 participants/month)

2.8.A. SAMPLE SIZE CALCULATION AND POWER

We provide power estimates for the % HDD (primary study outcome, Aim 1) and cigarettes smoked in the past week (primary outcome, Aim 2) and Inflammation and VACS index (primary and secondary outcomes, Aim 3).

Aim 1: primary study outcome, percent heavy drinking days in the past 30 days (% HDD): To define the limits of the study, we present power calculations to assess the differences we will be able to detect with reasonably high power for the primary study outcome: % HDD. It is expected that 400 participants will be enrolled into the study and we expect to have ~320 participants (80 participants in each of the 4 randomized groups) completing the 3-month follow-up, conservatively assuming 20% loss to follow-up. The following calculations assume 2-sided tests, with an overall significance level of 0.05. For the purposes of power calculations, we consider a simple, conservative setting based on a Bonferroni adjustment for multiple comparisons, although in our analyses we will use the Hochberg sequential correction method, an approach that will result in higher power than the Bonferroni method. To maintain an overall type I error rate of 5%, we assume each of the 3 pairwise comparisons will be conducted at an alpha level of 0.0167 for the following power calculations. Based on the Russia ARCH study participants who had ≥5 HDD and were regular smokers, the mean number of HDD in the past month was 14.7 and the standard deviation of % HDD was 27.2%. We expect the standard deviation will be similar in the proposed study. Given this assumption, the minimum detectable difference in % HDD at 3 months that the study can detect with 80% power is 14.1 for any of the 3 comparisons of interest (i.e., varenicline vs. cytisine; varenicline vs. NRT; cytisine vs. NRT), (e.g., 50% vs. 64.1% for varenicline vs. cytisine, respectively) using a 2-sided t-test. The study, therefore, has sufficient power to test for clinically important differences between any of the comparisons.

Aim 2: primary outcome, average number cigarettes per day in the past week: Based on the study by Litten et al.,[31] we assume the standard deviation for the average number of cigarettes smoked per day in the past week will be 7.6.[31] Given this and assuming a conservative 20% loss to follow-up at 3 months,

the minimum detectable difference in number of cigarettes smoked per day in the past week at 3 months that the study can detect with 80% power is 4.0 for any of the 3 comparisons of interest (e.g., 7 vs. 11 for varenicline vs. cytisine, respectively) using a 2-sided t-test.

Aim 3: primary outcome, biomarkers of inflammation: Based on ZINC HIV, the standard deviation of hsCRP at baseline was 3.6, with a mean of 5.9. We assume the standard deviation will be similar for the proposed study. Assuming a conservative 20% loss to follow-up at 3 months, the minimum detectable difference in hsCRP at 3 months that the study can detect with 80% power is 1.9 for any of the 3 comparisons of interest (e.g. 4.0 vs. 5.9 for varenicline vs. cytisine, respectively) using a 2-sided t-test.

Aim 3: secondary outcome VACS Index: Based on ZINC HIV, the standard deviation (SD) of the VACS Index at follow-up among those in the placebo group was 18.7. We assume the SD will be similar for the proposed study. Assuming a conservative 20% loss to follow-up at 3 months, the minimum detectable difference in the VACS Index at 3 months that the study can detect with 80% power is 9.7 for any of the 3 comparisons of interest (e.g. 30.0 vs. 39.7 for varenicline vs. cytisine, respectively) using a 2-sided t-test. **Interpretation:** A VACS score of 41 translates to a 5-year mortality rate of 19.6% whereas a VACS score of 34 translates into a 5-year risk of 14.4%; thus, a ~10 point difference corresponds to a ~25% mortality relative risk reduction in 5 years.

3. INTERVENTION

3.1 INTERVENTION OVERVIEW

The study will randomize 400 HIV+ persons with heavy alcohol consumption (by NIAAA definition of at risk drinking) who smoke. All existing Russia ARCH participants will be offered to be screened for St PETER HIV. Recruitment beyond First St. Petersburg Pavlov State Medical University (PSMU) will occur at Russia ARCH network hospitals including the major clinical HIV and addiction hospitals, local NGOs and through snowball recruitment. Some participants may be screened over the phone, but all interested potential study participants will ultimately be invited for an in-person screening of eligibility at PSMU. After eligible participants are consented and enrolled, the RA will complete screening for eligibility by conducting a rapid HIV test for non-ARCH participants, measuring participant blood pressure, breath alcohol level, and testing all women for pregnancy (urine). Once eligibility is confirmed, the RA will conduct the baseline interview and phlebotomy. Eligible participants will then be randomly assigned into one of four study arms: 1) Varenicline + NRT placebo; 2) Varenicline placebo + NRT; 3) Cytisine + NRT placebo; 4) Cytisine placebo + NRT. All participants will receive evidence-based counseling for alcohol and tobacco use, 1 active medication, and 1 placebo. Study medication will be provided by trained physicians, who will instruct participants in proper medication administration and adherence.

Study participants, investigators, staff, and physicians administering the medications will be unaware of specific group assignment (i.e., varenicline, cytisine, or NRT). The randomization, stratification, and assignment of participants to the 4 intervention groups will be guided and monitored by the URBAN ARCH Biostatistics and Data Management (BDM) Core. Although theoretically possible, it would not be practical or feasible to blind participants to whether they are taking varenicline or cytisine because the

regimens for these drugs are very different. The comparison of varenicline vs. NRT and cytisine vs. NRT will be double-blinded. Participants assigned to either partial agonist arm (varenicline or cytisine) will be blinded to whether the study medication is real or a placebo.

Throughout the course of the study, participants will be expected to come in for five in-person assessments and blood draw visits (baseline, 1-, 3-, 6-, and 12-months), additionally participants will have two in-person check ins at weeks two and 8 (2 months) and four check-ins via phone (weeks 1, 3, 6, 10). During these calls, participants will be monitored for adverse events.

3.2 RANDOMIZATION

Random assignment of participants into the 4 intervention groups will be conducted and monitored by the URBAN ARCH Biostatistics and Data Management (BDM) Core. The software package Statistical Analysis System (SAS) will be used to generate randomization lists to assign participants as they are enrolled into the trial. Participants will be randomized to groups in a 1:1:1:1 ratio using balanced blocks stratified by 3 factors: alcohol consumption (≥ 3 vs. < 3 heavy drinking days in the past week according to NIAAA risky drinking criteria, assessed via the first 7 days of the Timeline Follow Back), average daily cigarettes (≤1 vs. >1 pack per day, assessed via question 5 of the Tobacco Use section), and current antiretroviral therapy (ART) use [yes vs. no] to ensure balance across arms. Prior to initiation of recruitment processes, the study pharmacist will receive a list of medication box IDs and group assignment from the BDM core and will deliver a supply of packaged boxes of study medication to the study team.

Following completion of the baseline assessment, the RA will be directed to the electronic randomization screen in REDCap, which, once submitted, automatically assigns the participant to a randomization group. The RA then retrieves the box of study medication from the identified group, labels the box with the participant study ID, and enters the box number into REDCap, thus linking the two numbers.

3.3 INTERVENTION

Eligible participants will then be randomly assigned into one of four study arms: 1) Varenicline + NRT placebo; 2) Varenicline placebo + NRT; 3) Cytisine + NRT placebo; 4) Cytisine placebo + NRT. All participants will receive evidence-based counseling for alcohol and tobacco use at baseline. Study medication will be provided by trained physicians, who will instruct participants in proper medication administration and adherence.

Study medications will be obtained directly from the drug manufacturer - and delivered to the study pharmacist at PSMU. Dosing will follow standard recommendations. Placebo medications will not contain active ingredients. The placebo and active study medications will be indistinguishable by appearance and taste.

- Cytisine will be purchased from Katren in St. Petersburg, which serves as the distributor of medications to hospitals and pharmacies. Cytisine will be delivered to Bios Pharmaceuticals to be encapsulated with riboflavin, the biologic adherence measure. The procedure will be the same for the cytisine placebo, which will be composed of lactose and also contain riboflavin. Medication will be packaged in identical snap-top bottles, with each bottle containing 34 capsules.
- Varenicline will be purchased from Katren in St. Petersburg, which serves as the distributor of medications to hospitals and pharmacies. Varenicline will be delivered to Bios Pharmaceuticals to be encapsulated with riboflavin, the biologic adherence measure. The procedure will be the same for the varenicline placebo, which will be composed of lactose and will also contain riboflavin. Medication will be packaged in identical snap-top bottles, with each bottle containing either 11, 14, 28, or 56 capsules. Bottles with 11 capsules will contain pills of 0.5mg to be taken during week 1. These bottles will have a different color cap to distinguish them from bottles containing 14, 28 or 56 capsules. Bottles of 14, 28, and 56 capsules will contain pills of 1mg to be taken for the duration of treatment.
- NRT mouth spray will be purchased from Katren in St. Petersburg, which serves as the distributor of medications to hospitals and pharmacies. NRT mouth spray will be delivered to Vertex Pharmaceuticals for repackaging. An identical placebo will be manufactured by Vertex. Placebo solution contains propylene glycol, anhydrous ethanol, trometamol, glycerol, sodium hydrogen carbonate, levomenthol, mint flavour, cooling, flavour, sucralose, acesulfame potassium, hydrochloric acid and purified water. The taste and the smell of placebo will be identical to NRT mouth spray. NRT and placebo mouth spray consists of a small bottle (30 ml) of solution held in a dispenser with a mechanical spray pump. Each dispenser contains approximately 300 sprays.

Medication bottles will also be labeled. The medication labels will contain the following information:

FOR CLINICAL TRIAL USE ONLY

DRUG NAME (Nicotine or Placebo spray)

(Varenicline or Placebo capsules)

(Cytisine or Placebo capsules)

STORE 15-25˚C

USE BY dd/mm/yyyy

KEEP OUT OF REACH OF CHILDREN

The Food and Drug Administration (FDA) has indicated that an Investigational New Drug (IND) is not needed because the study is being conducted outside the United States.

3.3.A VARENICLINE + NRT PLACEBO

Participants randomized to this group will receive active varenicline for 12 weeks and NRT placebo mouth spray for 8 weeks. Medication dosing will follow recommended standards.

Varenicline (Chantix): Participants will be asked to take the study medication at the same time they take their non-study medication. If the patient is not on any non-study medication, they will be asked to simply take the study medication at the same time each day. Varenicline should be taken with a full glass of water. Participants will set a date to stop smoking one week from the baseline visit, and Varenicline dosing will start on the day of the baseline visit. Active varenicline will follow recommended dosing: we will begin at .5 mg for men and women (one pill on days 1-3 and two pills per day [morning and evening] on days 4-7) with a target dose of 1 mg twice daily during weeks 2-12.

Participants will be informed that when they try to quit smoking, with or without varenicline, they may have symptoms that may be due to nicotine withdrawal, including: urge to smoke, depressed mood, trouble sleeping, irritability, frustration, anger, feeling anxious, difficulty concentrating, restlessness, decreased heart rate, increased appetite, weight gain. Some people have even experienced suicidal thoughts when trying to quit smoking without medication. Sometimes quitting smoking can lead to worsening of mental health problems that are already present, such as depression.

Some people have had serious side effects while taking varenicline to help them quit smoking, including: new or worse mental health problems, such as changes in behavior or thinking, aggression, hostility, agitation, depressed mood, or suicidal thoughts or actions. Some people had these symptoms when they began taking varenicline, and others developed them after several weeks of treatment, or after stopping varenicline. These symptoms happened more often in people who had a history of mental health problems before taking varenicline, than in people without a history of mental health problems. In many people, these symptoms went away after stopping varenicline, but in some people symptoms continued after stopping varenicline. Participants will be instructed to use caution when driving or operating machinery until they know how varenicline affects them. Varenicline may make participants feel sleepy, dizzy, or have trouble concentrating, making it hard to drive or perform other activities safely. Participants will be instructed to decrease the amount of alcoholic beverages that they drink during treatment with varenicline until they know if varenicline affects their ability to tolerate alcohol. Some people have experienced the following when drinking alcohol during treatment with varenicline: increased drunkenness (intoxication), unusual or sometimes aggressive behavior, no memory of things that have happened. However, varenicline has been studied in people who drink, and may actually help reduce alcohol intake.

Serious side effects of varenicline may include:

Seizures - some people have had seizures during treatment with varenicline. In most cases, the seizures have happened during the first month of treatment with varenicline.

New or worse heart or blood vessel (cardiovascular) problems, mostly in people, who already have cardiovascular problems.

Sleepwalking can happen with varenicline, and can sometimes lead to behavior that is harmful to participants or other people, or to property.

Allergic reactions can happen with varenicline. Some of these allergic reactions can be life-threatening.

Serious skin reactions, including rash, swelling, redness, and peeling of the skin. Some of these skin reactions can become life-threatening.

In order to help prevent these adverse effects, participants will be instructed not to take more medication than what is provided by the study. Study clinicians will also be monitoring for signs of varenicline overdose. These adverse effects are not expected to be likely.

If baseline creatinine levels are indicative of reduced kidney function (< 30 cc/min), study investigators will reduce the dose of varenicline by 50%, which is the action recommended on the medication packaging and also reflects good clinical practice. Typically this dose reduction results in a total of 1.0 mg/day for those with seriously reduced renal function who are not on dialysis, and a total of 0.5 mg/day for those who are on dialysis.

NRT mouth spray placebo: Placebo NRT mouth spray will be identical in appearance and sensation to the active spray. Participants will be instructed to use enough placebo spray to control cravings. They will be asked to use one spray first when they would normally smoke a cigarette or have cravings to smoke. If cravings do not disappear within a few minutes, they will be asked to use a second spray. If 2 sprays are required to control cravings, future doses may be delivered as 2 consecutive sprays. Most smokers will require 1 - 2 sprays every 30 minutes to 1 hour. Participants will be instructed not to use more than 2 sprays per dose or 4 sprays every hour and will be asked not to use more than 64 sprays per day – this is equivalent to 4 sprays per hour for 16 hours. Study team will recommend that participants use at least 8 sprays per day (every 2 hours while awake) as a minimum. After 4 weeks, participants will be instructed to use the spray as much as they need within the recommended dosing. Participants may vary in how quickly they taper down, but will be instructed to keep using the spray everyday as much as is required to avoid smoking. Participants will be instructed to use the spray for 8 weeks.

All participants will receive a demonstration on proper use of the mouth spray. Participants will be asked to take care not to spray their eyes while administering the spray. To use, participants will be instructed to point the spray nozzle toward their open mouth and hold it as close to their mouth as possible. Participants will press the top of the dispenser to release one spray into their mouth and will be instructed not to inhale while spraying to avoid getting spray down their throat and asked not to swallow for a few seconds after spraying. Participants will be asked not to eat or drink when administering the mouth spray.

Participants may experience unwanted effects because by stopping smoking they have reduced the amount of nicotine they are taking. These effects include: irritability or aggression; impatient or frustrated; feeling low; anxiety; restlessness; poor concentration; increased appetite or weight gain; urges to smoke (craving); night time awakening or sleep disturbance; lowering of heart rate; constipation; bleeding gums; dizziness or light-headedness; sore throat, stuffy or runny nose; mouth ulcers, cough and/or symptoms of a common cold.

3.3.B. VARENICLINE PLACEBO + NRT

Participants randomized to this group will receive a varenicline placebo for 12 weeks and an active NRT mouth spray for 8 weeks. Medication dosing will follow recommended standards.

Varenicline placebo: As with active varenicline, placebo varenicline administration will follow recommended dosing: we will begin at one pill on days 1-3 and two pills per day starting on day 4. Participants will be asked to take their assigned pills at the same time they take their non-study medication. If the patient is not on any non-study medication, they will be asked to simply take the study medication at the same time each day. Participants will be instructed to take their pills with a full glass of water. Participants will set a date to stop smoking one week from the baseline visit, and dosing of the assigned intervention will start on the day of the baseline visit.

If baseline creatinine levels are indicative of reduced kidney function (< 30 cc/min), study investigators will reduce the dose of varenicline placebo, which is the action recommended on the medication packaging and also reflects good clinical practice. Typically this dose reduction results in a total of 1.0 mg/day for those with seriously reduced renal function who are not on dialysis, and a total of 0.5 mg/day for those who are on dialysis.

NRT mouth spray: The NRT mouth spray will contain 1 mg of nicotine per spray. Participants will be instructed to use enough nicotine spray to control cravings. They will be asked to use one spray first when they would normally smoke a cigarette or have cravings to smoke. If cravings do not disappear within a few minutes, they will be asked to use a second spray. If 2 sprays are required to control cravings, future doses may be delivered as 2 consecutive sprays. Most smokers will require 1 - 2 sprays every 30 minutes to 1 hour. Participants will be instructed not to use more than 2 sprays per dose or 4 sprays every hour and will be asked not to use more than 64 sprays per day – this is equivalent to 4 sprays per hour for 16 hours. Study team will recommend that participants use at least 8 sprays per day (every 2 hours while awake) as a minimum. After 4 weeks, participants will be instructed to use the spray as much as they need within the recommended dosing. Participants may vary in how quickly they taper down, but will be instructed to keep using the spray everyday as much as is required to avoid smoking. Participants will be instructed to use the spray for 8 weeks.

All participants will receive a demonstration on proper use of the mouth spray. Participants will be asked to take care not to spray their eyes while administering the spray. To use, participants will be instructed to point the spray nozzle toward their open mouth and hold it as close to their mouth as possible. Participants will press the top of the dispenser to release one spray into their mouth and will be instructed not to inhale while spraying to avoid getting spray down their throat and asked not to swallow for a few seconds after spraying. Participants will be asked not to eat or drink when administering the mouth spray.

Possible Untoward Effects, Their Symptoms & Treatment: Like all medicines, NRT mouth spray can have side-effects. As many of the effects are due to nicotine, they can also occur when nicotine is obtained by smoking.

Participants may experience unwanted effects because by stopping smoking they have reduced the amount of nicotine they are taking. Participants may also experience these effects if they underuse the spray before they are ready to reduce their nicotine intake. These effects include: irritability or

aggression; impatient or frustrated; feeling low; anxiety; restlessness; poor concentration; increased appetite or weight gain; urges to smoke (craving); night time awakening or sleep disturbance; lowering of heart rate; constipation; bleeding gums; dizziness or light-headedness; sore throat, stuffy or runny nose; mouth ulcers, cough and/or symptoms of a common cold.

Participants will be asked to stop using the spray and seek medical attention if they notice any of the following allergic reactions: swelling of the mouth, lips, throat and tongue, itching of the skin, swelling of the skin, ulceration and inflammation of the lining of the mouth.

Very common side-effects (may affect more than 1 in 10 people): hiccups (these are particularly common); throat irritation; headache; feeling sick (nausea); cough

Common side-effects (may affect up to 1 in 10 people): allergic reactions (hypersensitivity); burning sensation in the mouth; dizziness; taste disturbance or loss of taste; tingling or numbness of the hands and feet; toothache; stomach pain or discomfort; excessive gas or wind; vomiting; dry mouth; indigestion; diarrhea; tiredness (fatigue); sore and inflamed mouth; increased salivation

Uncommon side-effects (may affect up to 1 in 100 people): abnormal dreams; palpitations; fast heart rate/beat; sudden reddening of the face and/ or neck; high blood pressure; sudden constriction of the small airways of the lung that can cause wheezing and shortness of breath; shortness of breath; loss or damage to voice; throat tightness; burping (belching); swollen red sore tongue; mouth ulcers or blisters; numbness or tingling of the mouth; excessive sweating; itching; rash; hives (urticaria); unusual weakness; chest discomfort and pain; jaw muscle ache; general feeling of discomfort or being unwell or out of sorts (malaise); dry skin; muscle and bone pain; mouth and throat pain; sneezing; runny nose; blocked nose; inflammation of the gums.

Rare side-effects (may affect up to 1 in 1,000 people): difficulty in swallowing; decreased feeling of sensitivity especially in the mouth; feeling or wanting to be sick (vomit).

Other side-effects can include: abnormal beating of the heart; serious allergic reactions which cause difficulty in breathing or dizziness; swelling of the face or throat; blurred vison; watery eyes; dry throat; lip pain; stomach discomfort; redness of the skin; muscle tightness.

Clinicians will also be monitoring for signs of nicotine overdose. These adverse effects are not expected to be likely, as symptoms of nicotine overdose are extremely rare when using mouth spray as directed.

3.3.C. CYTISINE + NRT PLACEBO

Participants randomized to this group will receive active cytisine for 25 days and an NRT placebo mouth spray for 8 weeks. Medication dosing will follow recommended standards.

Cytisine (Tabex): Cytisine will be administered perorally according to the following schedule:

First 3 days: 1 tablet (1.5 mg) 6 times daily (every 2 hours, up to six tablets per day) with a parallel reduction of the number of cigarettes smoked.

4th to 12th day: 1 tablet every 2 1/2 hours (5 tablets daily)

13th to 16th day: 1 tablet every 3 hours (4 tablets daily)

17th to 20th day: 1 tablet every 5 hours (3 tablets daily)

21st to 25th day: 1 to 2 tablets daily

The following adverse effects are rather often observed at the beginning of cytisine treatment: changes in both taste and appetite, dryness in the mouth, headache, irritability, nausea, constipation, tachycardia, light elevation of the arterial pressure. The majority of the adverse effects can abate in the course of the treatment. In order to help prevent these adverse effects, participants will be instructed not to take more medication than what is provided by the study. Study clinicians will also be monitoring for signs of cytisine overdose. These adverse effects are not expected to be likely.

NRT mouth spray placebo: Placebo NRT mouth spray will be identical in appearance and sensation to the active spray. Participants will be instructed to use enough placebo spray to control cravings. They will be asked to use one spray first when they would normally smoke a cigarette or have cravings to smoke. If cravings do not disappear within a few minutes, they will be asked to use a second spray. If 2 sprays are required to control cravings, future doses may be delivered as 2 consecutive sprays. Most smokers will require 1 - 2 sprays every 30 minutes to 1 hour. Participants will be instructed not to use more than 2 sprays per dose or 4 sprays every hour and will be asked not to use more than 64 sprays per day – this is equivalent to 4 sprays per hour for 16 hours. Study team will recommend that participants use at least 8 sprays per day (every 2 hours while awake) as a minimum. After 4 weeks, participants will be instructed to use the spray as much as they need within the recommended dosing. Participants may vary in how quickly they taper down, but will be instructed to keep using the spray everyday as much as is required to avoid smoking. Participants will be instructed to use the spray for 8 weeks.

All participants will receive a demonstration on proper use of the mouth spray. Participants will be asked to take care not to spray their eyes while administering the spray. To use, participants will be instructed to point the spray nozzle toward their open mouth and hold it as close to their mouth as possible. Participants will press the top of the dispenser to release one spray into their mouth and will be instructed not to inhale while spraying to avoid getting spray down their throat and asked not to swallow for a few seconds after spraying. Participants will be asked not to eat or drink when administering the mouth spray.

Participants may experience unwanted effects because by stopping smoking they have reduced the amount of nicotine they are taking. These effects include: irritability or aggression; impatient or frustrated; feeling low; anxiety; restlessness; poor concentration; increased appetite or weight gain; urges to smoke (craving); night time awakening or sleep disturbance; lowering of heart rate; constipation; bleeding gums; dizziness or light-headedness; sore throat, stuffy or runny nose; mouth ulcers, cough and/or symptoms of a common cold.

3.3.D. CYTISINE PLACEBO + NRT

Participants randomized to this group will receive cytisine placebo for 25 days and an active NRT mouth spray for 8 weeks. Medication dosing will follow recommended standards.

Cytisine (Tabex) placebo: As with the active medication, Cytisine placebo will be administered perorally according to the following schedule:

First 3 days: 1 tablet 6 times daily (every 2 hours, up to six tablets per day) with a parallel reduction of the number of cigarettes smoked.

4th to 12th day: 1 tablet every 2 1/2 hours (5 tablets daily)

13th to 16th day: 1 tablet every 3 hours (4 tablets daily)

17th to 20th day: 1 tablet every 5 hours (3 tablets daily)

21st to 25th day: 1 to 2 tablets daily

NRT mouth spray: The NRT mouth spray will contain 1 mg of nicotine per spray. Participants will be instructed to use enough nicotine spray to control cravings. They will be asked to use one spray first when they would normally smoke a cigarette or have cravings to smoke. If cravings do not disappear within a few minutes, they will be asked to use a second spray. If 2 sprays are required to control cravings, future doses may be delivered as 2 consecutive sprays. Most smokers will require 1 - 2 sprays every 30 minutes to 1 hour. Participants will be instructed not to use more than 2 sprays per dose or 4 sprays every hour and will be asked not to use more than 64 sprays per day – this is equivalent to 4 sprays per hour for 16 hours. Study team will recommend that participants use at least 8 sprays per day (every 2 hours while awake) as a minimum. After 4 weeks, participants will be instructed to use the spray as much as they need within the recommended dosing. Participants may vary in how quickly they taper down, but will be instructed to keep using the spray everyday as much as is required to avoid smoking. Participants will be instructed to use the spray for 8 weeks.

All participants will receive a demonstration on proper use of the mouth spray. Participants will be asked to take care not to spray their eyes while administering the spray. To use, participants will be instructed to point the spray nozzle toward their open mouth and hold it as close to their mouth as possible. Participants will press the top of the dispenser to release one spray into their mouth and will be instructed not to inhale while spraying to avoid getting spray down their throat and asked not to swallow for a few seconds after spraying. Participants will be asked not to eat or drink when administering the mouth spray.

Possible Untoward Effects, Their Symptoms & Treatment: Like all medicines, NRT mouth spray can have side-effects. As many of the effects are due to nicotine, they can also occur when nicotine is obtained by smoking.

Participants may experience unwanted effects because by stopping smoking they have reduced the amount of nicotine they are taking. Participants may also experience these effects if they underuse the spray before they are ready to reduce their nicotine intake. These effects include: irritability or aggression; impatient or frustrated; feeling low; anxiety; restlessness; poor concentration; increased appetite or weight gain; urges to smoke (craving); night time awakening or sleep disturbance; lowering of heart rate; constipation; bleeding gums; dizziness or light-headedness; sore throat, stuffy or runny nose; mouth ulcers, cough and/or symptoms of a common cold.

Participants will be asked to stop using the spray and seek medical attention if they notice any of the following allergic reactions: swelling of the mouth, lips, throat and tongue, itching of the skin, swelling of the skin, ulceration and inflammation of the lining of the mouth.

Very common side-effects (may affect more than 1 in 10 people): hiccups (these are particularly common); throat irritation; headache; feeling sick (nausea); cough.

Common side-effects (may affect up to 1 in 10 people): allergic reactions (hypersensitivity); burning sensation in the mouth; dizziness; taste disturbance or loss of taste; tingling or numbness of the hands and feet; toothache; stomach pain or discomfort; excessive gas or wind; vomiting; dry mouth; indigestion; diarrhea; tiredness (fatigue); sore and inflamed mouth; increased salivation.

Uncommon side-effects (may affect up to 1 in 100 people): abnormal dreams; palpitations; fast heart rate/beat; sudden reddening of the face and/ or neck; high blood pressure; sudden constriction of the small airways of the lung that can cause wheezing and shortness of breath; shortness of breath; loss or damage to voice; throat tightness; burping (belching); swollen red sore tongue; mouth ulcers or blisters; numbness or tingling of the mouth; excessive sweating; itching; rash; hives (urticaria); unusual weakness; chest discomfort and pain; jaw muscle ache; general feeling of discomfort or being unwell or out of sorts (malaise); dry skin; muscle and bone pain; mouth and throat pain; sneezing; runny nose; blocked nose; inflammation of the gums.

Rare side-effects (may affect up to 1 in 1,000 people): difficulty in swallowing; decreased feeling of sensitivity especially in the mouth; feeling or wanting to be sick (vomit).

Other side-effects can include: abnormal beating of the heart; serious allergic reactions which cause difficulty in breathing or dizziness; swelling of the face or throat; blurred vison; watery eyes; dry throat; lip pain; stomach discomfort; redness of the skin; muscle tightness.

Clinicians will also be monitoring for signs of nicotine overdose. These adverse effects are not expected to be likely, as symptoms of nicotine overdose are extremely rare when using mouth spray as directed.

3.3.E. BEHAVIORAL COUNSELING

Participants in all study arms will receive brief (5 minute) evidence-based counseling for alcohol and tobacco use at baseline according to the following established guidelines:

Alcohol: National Institute on Alcohol Abuse and Alcoholism. A Pocket Guide for Alcohol Screening and Brief Intervention: Updated 2005 Edition. Rockville, MD: National Institutes of Health; 2007.[32]

Tobacco: Agency for Healthcare Research and Quality. Five major steps to intervention (the "5 A's"). Available from: http://www.ahrq.gov/professionals/clinicians-providers/guidelines-recommendations/tobacco/5steps.html.[33]

Alcohol Counseling:

The counseling approach for the trial will be adapted from the NIAAA Clinician's Guide as follows:

NIAAA Clinician's Guide	St. PETER
Step 1: Ask about alcohol use	Alcohol use assessed as part of the research interview; all have risky drinking at enrollment.
Step 2: Assess for alcohol use disorders (AUDs)	Performed during the research interview.
Step 3: Advise and assist (brief intervention) – described below	
Step 4: At follow-up: Continue support<br>Document alcohol use and review goals<br>Reinforce and support continued	N/A – counseling performed at baseline only

During the baseline assessment, RAs, who are all trained Russian addiction psychiatrists, will determine whether the participant meets DSM-5 criteria for an AUD and whether the AUD is mild, moderate, or severe. The RA will then tailor the brief alcohol counseling based on whether the participant has only at-risk drinking (all participants at enrollment) or an AUD.

- For participants who have only **at-risk drinking**, but who do not meet criteria for an alcohol use disorder, the RA will state his or her conclusion and recommendation about participant's alcohol use clearly and relate to the patient's concerns and medical findings, if present:
    - "You're drinking more than is medically safe."
    - "I strongly recommend that you cut down (or quit) and I'm willing to help."
    - The RA will gauge participant's readiness to change drinking habits by asking, "Are you willing to consider making changes in your drinking?" and responding accordingly.

No	Yes
Don't be discouraged—ambivalence is common. Your advice has likely prompted a change in your patient's thinking, a positive change in itself. With continued reinforcement, your patient may decide to take action. For now,<br>• **Restate your concern** about his or her health.<br>• **Encourage reflection** by asking patients to weigh what they like about drinking versus their reasons for cutting down. What are the major barriers to change?<br>• **Address barriers to change**	**Help set a goal** to cut down to within maximum limits or abstain for a time.<br>**Agree on a plan,** including<br>• what specific steps the patient will take (e.g., not go to a bar after work, measure all drinks at home, alternate alcoholic and nonalcoholic beverages).<br>• how drinking will be tracked (diary, kitchen calendar).<br>• how the patient will manage high-risk situations.<br>• who might be willing to help, such as significant others or nondrinking friends.<br>**Provide educational materials.**

- For participants who meet criteria for an **AUD**, the RA will state his or her conclusion and recommendation clearly and relate to the patient's concerns and medical findings if present.
  - "I believe that you have an alcohol use disorder. I strongly recommend that you quit drinking and I'm willing to help."
- The RA will negotiate a drinking goal (abstaining is the safest course for most patients with an alcohol use disorder); and consider recommending a mutual help group.

Tobacco Counseling:

Tobacco counseling is based off the Agency for Healthcare Research and Quality "Five major steps to intervention (the "5 A's"). The 5 A's are ask, advise, assess, assist, and arrange. Similar to the alcohol use counseling, these steps will be covered in the baseline assessment. Everyone is asked about tobacco use (step 1) and as all study participants will smoke at least an average of 5 cigarettes/day, all participants will be advised to quit tobacco (step 2). This advice should be clear, strong, and personalized.

Following advising participants to quit tobacco use, research assessors will assist participants in determining a quit plan. Strategies for implementing a quit plan include, setting a Target Quit Date (TQD). It is important to tell family, friends, and coworkers about quitting. It is also important to request understanding and support from them, to anticipate challenges to the upcoming quit attempt, and to remove tobacco products from the environment. Participants will be expected to set the TQD 1 week from the baseline assessment and should plan for abrupt smoking cessation on their target quit date. Study medication administration will begin on the day of their baseline visit (7 days before their quit date).

If the participant is at first unwilling to make a quit attempt, advise the participant that he/she agreed to follow study procedures and protocols earlier, and all participants in the study have agreed to reduce their alcohol and/or tobacco and accept study medications. Encourage all participants by reminding them that even taking small steps by using medications to eliminate tobacco use is a step in the right direction. These small steps can take the form of a "practice" quit attempt, which is identical to the TQD noted above, but which evidence shows may be interpreted by smokers as less formidable if it is considered "practice."[34] Evidence also shows that abrupt quitting, rather than gradual reduction, results in higher quit rates.[35]

Participants will be advised to let their primary care physician know that they are making a quit attempt.

Participants will be reminded that all medications in the study reduce withdrawal symptoms.

Participants will be provided with written material on the benefit of reducing/quitting both alcohol and tobacco.

Participants will be provided with the following recommendations:

1. On enrollment day, reduce cigarettes per day by half. For example, if someone is smoking 20 cpd, they should reduce to 10 cpd on the day they start medication.

2. Over the remainder of the first week, reduce at participants' own pace to zero by Target Quit Day (TQD--1 week after enrollment). For example, reduce from 10 cpd to zero over days 2-7.

3.4 MEDICATION CONSIDERATIONS

If reductions in medication are required, the following approach will be taken for each study drug:

For participants randomized to Varenicline/Varenicline placebo, reduce to 1 pill per day (instead of 1 pill twice daily).

For participants randomized to Cytisine/Cytisine placebo, reduce the dose by ½.

For mouth spray, reduce use to a level with which participants are comfortable and a dose that they can tolerate.

3.4.A SYMPTOM MONITORING

The study staff (research clinicians [addiction physicians with extensive experience performing pharmacotherapy trials]) will provide participants with the correct medication packages and advice on how to take the pills and utilize the mouth spray. The staff will be trained to assess for adverse medication effects and will follow established protocols for identifying and monitoring any ongoing adverse events, including referral to treatment as appropriate. Study participants will be actively monitored for adverse events, particularly those related to varenicline in the most recent FDA's Drug Safety Announcement. Symptoms will be assessed weekly for the first month (and monitored more frequently, if necessary) and biweekly thereafter by trained clinical staff, while the participants are administered study medications. Medication side effects will be monitored through a medication side effect checklist adapted from Lerman et al.'s (2015) "Use of the nicotine metabolite ratio as a genetically informed biomarker of response to nicotine patch or varenicline for smoking cessation: a randomised, double-blind placebo-controlled trial" study published in *The Lancet Respiratory Medicine.*[36] It should be noted that this symptom checklist reflects a highly conservative approach, since many of the symptoms on the checklist were found to be *unrelated* to varenicline or nicotine replacement in a recent 8,000 person trial, EAGLES, that was mandated by the FDA (*Lancet*, Anthenelli et al, 2015).[37]

3.4.B. ADHERENCE

Medication adherence will be assessed at each study visit using the direct (Riboflavin) and indirect (pill counts and self-report) measures.

Direct Adherence Measures

Riboflavin (50 mg), a vitamin yielding a change in urine color, will be added to both active and placebo capsules. Participants will be informed that the color change is harmless. At this dose, Riboflavin is

expected to remain in the system at detectable levels for up to 24 hours. At each study visit post-baseline (while taking study medication), participants will be asked to provide a urine sample which will be visually inspected for the presence or absence of Riboflavin in a room with low ambient light, using ultraviolet (UV) light at the long wave setting (33 mm).

Indirect Adherence Measures

Pill Counts

Participants will be instructed to bring any unused medication to each study visit post-baseline. The RA will count and record the number of remaining pills. The data management team will extrapolate the amount of medication taken and determine the measure of adherence.

Self-Report

Medication adherence will also be measured through self-report using the modified Adult AIDS Clinical Trial Group (AACTG) ART adherence questions.

Adherence Aids

During each study visit medication instructions will be reviewed and strategies for adherence discussed with each participant. Adherence plans will be individually tailored to each participant, depending on his or her reason for non-adherence. To further increase medication adherence, an automated text message will be sent daily (on week days), with the option for participants to reduce text messages to twice per week (Tuesday and Friday), reminding participants to take their study medication.

The message will read: Пожалуйста, принимайте препарат исследования регулярно
Translation: Please take study medication regularly

Participants will be able to reduce the frequency to 2 days per week or opt out of text message reminders entirely at any time throughout the study.

Participants will also be encouraged to set a reminder in their phone to take the study medication each day.

3.4.C. MEDICATION DISBURSEMENT

Medication inserts will be provided to participants at baseline.

Medication distribution will be as follows:

Varenicline and Varenicline Placebo:

The following medication bottles will be prepared for study participants by the study pharmacist:

1. 1 bottle of 11 capsules that are 0.5mg to be taken during week 1. This bottle will have a cap of a different color to distinguish it from the rest of the bottles.

2. 1 bottle of 14 capsules that are 1 mg
3. 1 bottle of 28 capsules that are 1mg
4. 1 bottle of 56 capsules that are 1mg
5. 1 bottle of 56 capsules that are 1mg

   (bottles 4 and 5 are the same)

At baseline, participants would get bottles 1, 2, and 3. This would be a one-month supply of medication. At 2-week visit, participants would get one bottle of medication, bottle 4 (one-month supply). At 1-month visit, participants would get the remaining bottle 5 (one-month supply).

Cytisine and Cytisine Placebo

- 34 capsules/bottle
- Participants will receive the entire supply of Cytisine medication at the baseline study visit (3 bottles).

NRT and NRT Placebo

Each bottle of NRT mouth spray (active and placebo) will contain 300 doses of NRT. We estimate that most participants will require 5 bottles of spray for the duration of the study to be distributed according to the following schedule:

At baseline, participants will receive 3 bottles of study spray and at 2-weeks, they will receive the remaining 2 bottles of the study spray.

Dosing will depend on participant cravings, thus some participants may require additional study spray bottles throughout the study. For participants that require additional spray bottles, boxes with active spray and placebo will be provided to the study team. The boxes will be labeled with a code known only to the pharmacist. In the event that additional bottles of spray are needed, the research assessor will contact the pharmacist and provide her with the participant’s ID, based on the participant’s study ID, the pharmacist will be able to indicate which labeled box to use to dispense the extra spray. The same approach will be taken should participants require extra study capsules.

3.4.D. LOST OR STOLEN STUDY MEDICATION

If participants report lost or stolen medication, they will be provided with extra study medication. If participants report losing medication more than once, the study team will be alerted and the case discussed to determine a plan of action.

3.4.E. DISCONTINUATION OF STUDY MEDICATION

Despite known side effects of partial agonists (nausea being the most common), most people taking these medications do not discontinue them due to side effects. Before medication is discontinued it is important to encourage participants to take medication with a full glass of water and food. This usually reduces medication side effects. Those who discontinue medication will be followed and analyzed by intention to treat.

Participants found to be pregnant during the study will have their study medication discontinued, but will still be followed-up for the duration of the study. Participants who report pregnancy outside of study visits will be instructed to immediately discontinue their study medication and requested to come in for a confirmatory urine pregnancy test.

3.5 Schedule of Data Collection

		Pre-screen	Screener and Baseline Visit		1 week call	2 week visit	3 wk call	1 mo visit	Calls wks 6 and 10	2 mo visit	3 mo visit	6 mo visit	12 mo visit
			Screener	Baseline									
Screening	Screening Questions	X	X										
	Verification of HIV and non-pregnancy, BAC and BP measures		X										
Enrollment	Sign Informed Consent		X										
	Complete contact information/verify numbers			X									
	Randomization			X									
Laboratory	Clinical Values			X				X			X	X	X
	BAC and CO Monitoring			X				X			X	X	X
	Pregnancy Test		X			X		X		X	X		
	Urine Nicotine metabolite testing			X							X		
	Purple1: Hemoglobin			X							X		
	Purple1: Platelets			X							X		
	Purple1: HIV RNA			X							X		
	Purple1: DBS			X				X			X		
	Purple1:Plasma			X				X			X	X	X
	Green: CD4			X							X		
	Red1: HS CRP			X							X		
	Red1: Cholesterol			X							X		
	Red1: eGFR			X							X		
	Red1: AST/ALT			X							X		
	Red1: HCV Ab			X							If neg		
	Red1: Serum			X				X			X	X	X
	Purple 2: Whole blood			X									
	CPT: Heparin plasma and PBMC			X									
	Purple 3: Plasma			X				X			X	X	X
Asses	Full Study			X				X			X	X	X

sment	Assessment												
	Adherence only				X	X	X		X	X			
Intervention	Symptom Management/Adverse Events			X	X	X	X	X	X	X	X		
	Provide Medication Instructions			X		X		X		X			
	Give Study Medication			X		X		X					
	Observe Ingestion			X									
	Discuss Adherence			X	X	X	X	X	X	X			
	Assess Adherence				X	X	X	X	X	X	X		
	Provide Counseling			X									
Other	Provide Resource Card			X									
	Compensate for Participation			X	X	X	X	X	X	X	X	X	X
	Report Adverse Events			X	X	X	X	X	X	X	X	X	X
	Complete Tracking Forms			X	X	X	X	X	X	X	X	X	X

3.5.A. VISIT WINDOWS

1 Week Medication Call

- Window open: 5 days post baseline
- Target date: 7 days post baseline
- Window close: 10 days post baseline
- Window length: 5 days

2 Week Visit

- Window open: 11 days post baseline
- Target date: 14 days post baseline
- Window close: 20 days post baseline
- Window length: 9 days

3 Week Medication Call

- Window open: 18 days post baseline
- Target date: 21 days post baseline
- Window close: 25 days post baseline
- Window length: 7 days

1 Month Visit

- Window open: 25 days post baseline
- Target date: 28 days post baseline
- Window close: 41 days post baseline
- Window length: 16 days

6 Week Medication Call
- Window open: 38 days post baseline
- Target date: 42 days post baseline
- Window close: 50 days post baseline
- Window length: 12 days

2 Month Visit
- Window open: 49 days post baseline
- Target date: 56 days post baseline
- Window close: 68 days post baseline
- Window length: 19 days

10 Week Medication Call
- Window open: 65 days post baseline
- Target date: 70 days post baseline
- Window close: 80 days post baseline
- Window length: 15 days post

3 Month Visit
- Window open: 78 days post baseline
- Target date: 84 days post baseline
- Window close: 140 days post baseline
- Length: 62 days

6 Month Visit
- Window open: 141 days post baseline
- Target date: 180 days post baseline
- Window close: 252 days post baseline
- Length: 111 days

12 Month visit
- Window open: 253 days post baseline
- Target date: 360 days post baseline
- Window close: 450 days post baseline
- Window length: 197 days

*Phone and visit windows may overlap, but RAs will always prioritize visits over phone calls.

3.6 DATA SOURCES

3.6.A QUESTIONNAIRES

Questionnaires will be administered at baseline, 1-, 3-, 6-, and 12-month study visits to collect information about participant demographics (e.g., age, gender), general and mental health and health-related behaviors such as substance use.

3.6.B. BLOOD

Blood will be collected at baseline, 1-, 3-, 6-, and 12-month study visits to assess the following:

Test	Timepoint	Fresh/Frozen
Red Tube (9 mL at baseline and 3 months; 6 mL at 1,6, and 12 months)		Testing on fresh samples; serum stored for future use, including TMAO testing, in 0.5mL aliquots.
HS CRP	Baseline, 3-months	
Cholesterol	Baseline, 3-months	
HCV Ab	Baseline. 3-months if negative at baseline	
eGFR (creatinine)	Baseline, 3-months	
AST/ALT	Baseline, 3 months	
Aliquoted Serum	Baseline, 1, 3, 6, 12-months	
Purple EDTA (6 mL)		Testing on fresh samples; plasma stored for future IL-6, oxidative stress, and BNP testing (0 and 3-mo) in 0.5mL aliquots. Samples may also be used for future testing not listed above to study altered coagulation, microbial translocation, hepatic, and cardiometabolic diseases.<br>.
Hemoglobin	Baseline, 3-months	
Platelets	Baseline, 3-months	
HIV RNA	Baseline, 3-months	
DBS	Baseline, 1-, and 3-months	
Aliquoted plasma	Baseline, 1, 3, 6, 12-months	
Green Heparin (2 mL)		Testing on fresh samples
CD4	Baseline, 3-months	
Purple EDTA (5 mL)		Whole blood stored for future testing
Stored as whole blood in a 5mL polypropylene centrifuge tube	Baseline	
Cell Prep Tube (8 mL)		Stored for future testing as 2 0.5mL aliquots of PBMCs and 1 mL aliquots of plasma
Aliquoted heparin plasma and PBMCs	Baseline	
Purple EDTA (9 mL)		

Aliquoted plasma	Baseline, 1, 3, 6, 12-months	Stored for future testing in 0.5 mL aliquots.

1. Cholesterol
    a. Measured at baseline and 3-month study visits as part of the Reynold Risk Score, which assesses CHD risk and has been validated in large cohorts of men and women, and is routinely used in clinical practice.
2. hsCRP, IL-6
    a. hsCRP and IL-6 are biomarkers of inflammation significantly associated with heavy alcohol consumption, smoking, incident CHD, and mortality in HIV+ people. These measures will be collected at baseline and 3-month study visits.
    b. hsCRP is also used to calculate the Reynolds Risk Score
3. Rapid HIV Ab
    a. Will be measured at baseline for screening purposes, to confirm participants HIV status.
4. CD4, HIV-1 RNA
    a. Measured at baseline and 3-month study visits as part of the VACS Index, a validated surrogate measure of mortality in HIV+ people.
5. Hemoglobin and platelets
    a. Measured at baseline and 3-month study visits as part of the VACS Index.
6. Creatinine
    a. Measured at baseline and 3-month study visits as part of the VACS Index.
7. HCV Ab, AST, ALT
    a. HCV Ab will be measured at baseline, and if negative at baseline, again at 3 months, as part of the VACS Index. AST, ALT will be measured at baseline and 3-month study visits as part of the VACS Index.
8. NMR and tobacco-genetics
    a. Nicotine Metabolite Ratio (NMR) will be collected at baseline and 3-months and used to interpret study results. NMR is a genetically-informed biomarker of nicotine metabolism, knowing metabolizer type (fast/normal vs. slow) will inform the results comparing partial agonists to NRT for tobacco. This blood test will be run in Rachel Tyndale's lab in Toronto.
    b. Tobacco-genetic variants (variations in several genes that are known to predict nicotine metabolism, difficulty quitting, and performance with smoking-cessation medication) will be collected at baseline and used to interpret study results. These blood tests will be conducted in Dr. Tyndale's lab in Toronto.
9. Oxidative stress
    a. Oxidative stress will be measured at baseline and 3-months on stored plasma. High levels of oxidative stress can lead to cell and tissue damage through the increase of oxygen reactive species. Oxidative stress is associated with diabetes, atherosclerosis, high blood pressure, heart disease, and other inflammatory conditions.
10. TMAO
    a. Trimethylamine N-oxide (TMAO) will be measured at baseline and 3-months on stored serum. Increased TMAO is associated with cardiovascular disease events.
11. BNP

a. B-type natriuretic peptide (BNP) will be measured at baseline and 3-months on stored plasma. BNP is a biomarker of ventricular stretching.

Unsuccessful Draw Protocol

At baseline and 3-month visits, a blood draw will be considered successful if the 6mL purple EDTA tube and 9mL red tube are full. If a blood draw is unsuccessful at baseline (i.e., the EDTA and red tube are not completely full), the participant will not continue with St PETER baseline procedures (assessment, randomization, intervention) and will receive 1/3 compensation. Blood collected during an unsuccessful blood draw will be processed and stored. Participants will have 30 days upon completion of the screener to complete the baseline blood draw and baseline visit and receive full compensation. Participants with unsuccessful baseline blood draws will receive 1/3 ($11) compensation at the initial attempt, 1/3 ($11) at their second attempt, and the final 1/3 ($11) at their third attempt. If the second attempt is successful, they will receive the remaining 2/3 ($22) of the compensation at that time.

		St PETER

Blood collected at a re-draw requires the collection of both EDTA (6mL) and red (9 mL) tubes, regardless of if either tube was collected at any previous draw attempt. For participants with an incomplete (but successful) blood draw at baseline, tubes 4 (if the participant still reports smoking), 5, and 6 will be collected at the 2-week visit. If a participant fails to complete the baseline blood draw, he/she will be disenrolled after 30 days or 3 unsuccessful blood draw attempts.

If a blood draw is unsuccessful at the 1-, 6-, or 12-month study visit, it will be considered missing data and no re-draw attempts will be made.

If the blood draw is unsuccessful at the 3-month study visit, the participant will be given partial (1/2) compensation for completion of the assessment. The participant has 30 days to complete the blood draw attempt. Blood draw attempts will occur weekly (up to 4 times). The remaining ½ ($16) of the compensation will be divided between the 4 weekly attempts ($4 for each attempt). If the second of third attempts are successful, participants will receive the remaining 2/4 or 3/4 ($8-$12) at that time.

Baseline	Timing of blood draw	Blood draw to take place prior to assessment; If blood draw is not successful, no assessment is conducted.
	Compensation	If blood draw is not successful, participant receives partial (1/3) compensation.
	Window	30 days after completion of screener
	Final attempt	Disenroll after 3 unsuccessful attempts or 30 days; provide full compensation at third/final attempt
Follow up* (3-month visit only)	Timing of blood draw	If blood draw is not successful, proceed to assessment
	Compensation	Given partial (1/2) compensation for completion of assessment
	Window	30 days after completion of assessment
	Final attempt	4 weekly attempts; at final attempt provide full compensation

Participants in the continued observational cohort will not have minimum draw requirements.

3.6.C. URINE

A pregnancy test will be administered by trained clinical research staff at screening to determine eligibility and at each study visit. Pregnant women will be excluded from the study due to some reports suggesting possible adverse events with study medications. Participants found to be pregnant will discontinue their study medication but will still be followed-up for the duration of the study.

Urine will also be used to conduct nicotine metabolite testing on all participants at baseline and 3-month study visits to validate participant smoking self-report. The nicotine metabolite testing panel will include nicotine, cotinine, 3-hydroxy cotinine, nornicotine, and anabasine. This testing will take place at the Helix lab in St. Petersburg.

Urine will be used to measure adherence to the study medications, as described in section 3.4b Adherence.

3.6.D. CARBON MONOXIDE

Carbon monoxide (CO) in the lungs will be measured at all in-person study visits, as part of the secondary outcome of CO-validated smoking cessation. The PICO+Smokerlyzer model will be used to measure the amount of CO (in parts per minute) in participant’s breath and is a way to biochemically establish

smoking status. Furthermore, CO monitoring can act as a motivational visual aid to encourage participants towards smoking cessation and to measure their progress while quitting.

3.6.E. BREATH ALCOHOL CONTENT

Breath alcohol content will be tested at all in-person study visits to encourage truth telling. Breath alcohol testing only reflects recent alcohol use. Breathalyzer used for the study is the Dingo E-010 manufactured by Sentech Korea Corp. and obtained in Russia.

3.6.F. DRIED BLOOD SPOTS

Dried blood spots (DBS) will be collected at baseline, 1-, and 3-month study visits for future phosphatidylethanol (PEth) testing. PEth will be tested at the United States Drug Testing Laboratories, Inc. (USDTL).

4. OBSERVATIONAL COHORT

All current Russia ARCH participants who do not initially meet eligibility criteria for St PETER HIV will be invited to continue their participation in the ongoing Russia ARCH cohort. The cohort experience will entail an assessment and blood draw at baseline and 12 months. At the 12-month visit, those Russia ARCH participants who initially did not meet enrollment criteria for St PETER HIV will be re-evaluated, and if eligible, will be offered enrollment into St PETER HIV, if enrollment is still ongoing. By continuing to follow existing Russia ARCH participants who did not enroll in St PETER HIV, this observational cohort can be used to improve our understanding of the natural history for smoking and heavy drinking among HIV+ people who either did not enroll or who did not meet St PETER HIV entry criteria.

5. STUDY PROCEDURES

5.1 RECRUITMENT

All existing Russia ARCH participants, who have agreed to be contacted for future studies, will be screened for St PETER HIV. Recruitment beyond Russia ARCH cohort will occur at non-governmental organizations (NGOs) and Russia ARCH network hospitals including the major clinical HIV hospitals (Botkin Infectious Disease Hospital, Leningrad Regional AIDS Center, St. Petersburg City AIDS Center) and addiction hospitals (Leningrad Regional Center for Addictions, St. Petersburg City Addiction Hospital) through notification of patients and providers at these sites and through word-of-mouth (i.e. snowball recruitment). At each of the recruitment sites flyers with information about the trial will be distributed to clinicians and peers working with HIV-infected patients. Study flyers will also be placed in registration areas. Flyers will provide a phone number (at FSPSMU) for interested individuals to call to

learn more about the study and undergo initial screening over the phone. On occasion, Pavlov research assessors may travel to screen participants at the affiliated clinical locations. Enrolled participants will be given flyers to distribute to potential participants who might be interested in the trial. If potential participants are interested, they will be responsible for directly contacting study staff listed on the flyer to be screened for eligibility. Referred participants who enroll in the study will provide the researcher with the name of the study participant who referred them. The referring study participant will be compensated 300 rubles for a successful referral at their next visit to Pavlov.

5.2 SCREENING

Screening may take place over the phone or in-person. Verbal consent for screening will be obtained from all potential participants. Potential participants will be asked by a research assessor, either on the phone or in-person, their age, HIV infection status, current alcohol consumption and smoking status, interest in cutting down or quitting smoking or drinking, confirm residence within 100km of St. Petersburg, and possession of a phone, and two contacts to assist with follow-up, and ability and willingness to comply with all study protocols and procedures. If the participant is not fluent in Russian, has a cognitive impairment resulting in inability to provide informed consent, reports breastfeeding or being pregnant or planning to get pregnant, has a serious psychiatric illness, history of pheochromocytoma or seizures, has taken smoking cessation medication in the past 30 days, had acute coronary syndrome in the past month, has a history of Buerger's disease, is currently taking anti-tuberculosis medication, is currently taking Galantamine or Physostigmine, or has a known allergy to cytisine or varenicline , then s/he will also be deemed ineligible. After informed consent is signed, participant blood pressure and breath alcohol content will be measured, a confirmatory pregnancy test will be done for all female participants and rapid HIV testing will be conducted for all non-Russia ARCH participants. Data collected on participants who screen out will be kept in order to have an accurate record of the rate of enrollment among those screened for participation and to be able to identify reasons why potential participants are ineligible. The data will not have identifying information.

If a participant is screened over the phone, participant will be re-screened during his/her scheduled baseline visit.

5.3 INFORMED CONSENT

Research assessors will conduct the consent process as well as obtain written consent. After eligibility and interest in enrollment is determined, an RA will administer and document the informed consent of the participant in a private location. The study will be explained to eligible participants who will be offered participation in the study. Research assessors will answer any questions the participants may have including risks, benefits and alternatives (including non-participation) to participation, and will provide written materials describing the study. If participants are unsure whether they would like to participate, they will be allowed any amount of time they need to consider participation in the study. If

the participant is not able to make a decision on the day of the initial visit, they will be invited to contact the study team once they have made their decision, at which point they will be re-screened, if more than 3 days have passed since their initial screening. The written informed consent (in Russian), including the risks, benefits and alternatives, will be signed by the participant and the research assessor. As part of the informed consent process we will make it explicit to the participants that their involvement in the study does not constitute medical treatment and that they will not receive any HIV medical care as part of the study. We will provide a handout with information on addiction and HIV treatment services to participants at the baseline visit. A copy of the informed consent will be provided to the participant and a copy will be maintained by the research team. Potential participants will be informed that refusal to participate will not affect their medical care at FSPSMU in any way and they will be informed of their right to drop out of the study at any time.

5.3.A. OBSERVATIONAL COHORT

If a participant is ineligible for the trial and was previously enrolled in the Russia ARCH cohort, the RA will offer participation in the observational cohort. The RA will explain the difference between the trial and cohort (there will only be a baseline and 12-month in person assessment for Russia ARCH cohort participants). If the participant is interested, the RA will continue with the informed consent process (a separate Informed Consent Form from the trial will be provided).

5.4 VISIT FLOW

After eligible participants are consented and enrolled, the RA will follow these steps at the **Baseline visit**:

- Conduct HIV, pregnancy testing and measure blood pressure and breath alcohol content to verify eligibility
- Collect locator/contact information and check contact phone numbers
- Send participant for phlebotomy, collect clinical data (height, weight, blood pressure and urine sample [to send for nicotine metabolite testing], and obtain carbon monoxide reading)
- Administer assessment questionnaire
- Randomize participant
- Assess baseline symptoms
- Introduce study medication and instructions and develop an adherence plan
- Conduct behavioral counseling
- Compensate participant, provide list of resources, appointment reminder card, participation card, daily alcohol and cigarette use calendar, and schedule next visit

At the **2-week and 2-month medication visit,** the RA will perform the following:

- Review and update locator/contact information, verifying new numbers, as necessary
- Collect a urine sample to check for pregnancy and adherence

- Perform symptom monitoring
- Assess medication adherence
- Review medication instructions and adherence plan
- Compensate participant and schedule next visit

During the **1-, 3-, 6-, and 12-month assessments,** the RA will:

- Review and update locator/contact information, verifying new numbers, as necessary
- Collect a urine sample to check for pregnancy, adherence, and perform nicotine metabolite testing (pregnancy and adherence will be checked at 1- and 3-month visits only, and nicotine metabolite testing will be done at 3-months only)
- Send participant for phlebotomy, collect clinical data (height, weight, blood pressure) and obtain carbon monoxide and Breathalyzer readings
- Perform symptom monitoring (at 1- and 3-month visits only)
- Assess medication adherence (at 1- and 3-month visits only)
- Administer assessment questionnaire
- Review medication instructions and adherence plan (at 1-month visit only)
- Compensate participant, provide daily cigarette and alcohol use calendar (not at 12 months), and schedule next visit

5.5 QUALITY ASSURANCE

Informed consent quality assurance

The RA will review Informed Consent Forms (ICFs) for completeness with the participant present. Items to check will include, but are not limited to: responses/initials collected for all questions, correct version of ICF used, signed and dated by both subject and RA. Both the RA and project manager will review ICFs weekly for completeness.

Assessment quality assurance

During the assessment, if the participant provides conflicting answers or answers that did not make logical sense (either within the same section or between sections), the RA will gently try to help the participant arrive at more logical answers. However, the RA will not force the participant to change his or her answers. Certain quality assurance checks are built into the assessment. The system will flag any inappropriate responses and prevent the RA from continuing until the issue is resolved. The RA will review the self-administered section with the participant present. If many “refused” options are selected, the RA will offer the participant the opportunity to complete those sections (the RA will accept the participant’s refusal if he or she does not wish to complete the section). The RA will never guess to correct a mistake. The only instance when a change can be made to the completed assessment is in the event that the RA is 100% certain that an error was made in data entry.

5.6 COMPENSATION

The 400 participants in the trial will receive the equivalent of US $33 in goods or cash for their participation at baseline 1-, 3-, and 6-month study visits. Participants will receive the equivalent of US $40 for the final 12-month study visit. All visits will involve the collection of blood for laboratory testing. Participants will also receive in total the equivalent of US $14 for short medication check phone calls (4 phone calls, $3.50/call) and the equivalent of US $15 for short in-person medication visits at 2 weeks and 2-months post-baseline. Participants will have the option to delay phone call compensation, which they would receive in phone minutes, until the next in-person study visit. Similar compensation has been used in a previous collaborative Russian-Boston research study and was deemed by the FSPSMU Institutional Review Board (IRB) to be an appropriate, non-coercive, amount of funds for involvement in a clinical research project.

Participants of the extended Russia ARCH cohort who are not eligible for the trial will receive the equivalent of US $25 for participation in the baseline and 12-month assessment visit.

Participants who provide updated contact information to research assessors in between research visits (i.e. not during the research assessment) will receive 200 rubles (approximately $3) in goods or currency. In order to celebrate participant engagement and improve retention, the study team will provide an equivalent of 400 rubles (approximately $7) in the form of a supermarket gift card for enrollment milestones at the 3-month and 12-month study visit.

Phone interviews will be offered to participants who are not able to make it to Pavlov for an in-person visit (partial [1/2] compensation will be provided for completion of the visit over the phone). Participants with unsuccessful baseline blood draws will receive 1/3 ($11) compensation at the initial attempt, 1/3 ($11) at their second attempt, and the final 1/3 ($11) at their third attempt. If the second attempt is successful, they will receive the remaining 2/3 ($22) of the compensation at that time. For an unsuccessful draw at 3 months, the participant will be given partial (1/2; $16) compensation for completion of the assessment. The remaining ½ ($16) will be divided between the 4 weekly attempts ($4 for each attempt). If the second of third attempts are successful, participants will receive the remaining 2/4 or 3/4 ($8-$12) at that time.

Phone assessments will be completed for participants who are unable to come in to Pavlov due to COVID-19-related restrictions. Participants will receive full compensation for these phone assessments. Upon the restrictions being lifted, participants will be invited back to Pavlov to complete 6 and 12-month study visit procedures. At this time the assessment will also be re-administered if more than 30 days have passed since the participant’s phone assessment. Participants will receive full compensation for both phone and in-person visits.

5.7 RETENTION

Baseline visit: Retention begins at baseline by ensuring that the participant enjoys the experience of participating in the study, by explaining the informed consent and what would happen in the study, and by collecting excellent contact information, including both the address where the participant is registered and the address where the participant is currently staying. Participants will be asked to provide contact information for 4-5 alternative contacts who may know their whereabouts. Alternative contacts can include friends, family members, and social workers. Participants will be asked if any of their friends are participating in the study and to include them as alternative contacts, if possible. Contact numbers must be verified by calling the numbers with the participant present, using the following script:

> I am calling from Pavlov University. Your friend/relative [NAME] is here with me and just enrolled in a study. He/she has listed you as an alternative contact. We will only call you if we are having trouble reaching [NAME] to see if you can help us connect with them. Today I am just calling to confirm that this number is active.

If the alternative contact cannot be reached at the baseline visit, the RA will try to reach the contact again at the next in-person study visit. If the RA is unable to reach the contact at the following study visit, the participant will be asked to provide a different alternative contact. Participants will also be asked for their email address and membership to any social networking platforms.

All visits: Participants will be offered tea, coffee, water, and snacks at each study visit to make their experience in the research study more enjoyable.

RA will offer to help participants add the next scheduled study visit to the calendar in their phone and set a reminder in their phone.

Follow up visits: Contact information for participant and alternatives will be reviewed and updated at every visit.

Other strategies: Participants will be contacted by telephone with appointment reminders and email if one is provided. The study team will also utilize social networking to connect with participants. If participants are unable to be reached via phone, in addition to attempting to reach them via text messaging and email, participants will be sent private messages on Vkontakte (Russian social network) utilizing an existing standard script to remind them of their upcoming study visit. No sensitive information will be revealed or ascertained using this method. Study participants will be asked to contact the study team if their phone number changes between study visits; participants will be compensated 200 rubles in goods or currency for this information. All no-shows will be followed up to reschedule appointments.

Participants who are continually unable to be reached will be sent a letter asking them to contact the study team. In the letter, compensation will be offered to participants ($5) for calling the study team and updating their contact information. The letter will explicitly state that additional compensation would be received when the participant comes in for his/her scheduled study visit.

In order to celebrate continued participant engagement, we will provide an equivalent of 400 rubles (approximately $6) in the form of a supermarket gift card for enrollment milestones at the 3-month and 12-month study visit in addition to the pre-specified visit compensation.

Participants will be sent a happy birthday text message and receive sweets when they come in to complete a study visit near the time of major Russian holidays (i.e. New Year, Christmas).

The team will collaborate with affiliated hospitals by contacting staff to find out if any hard-to-reach participants are hospitalized at that location. If so, RAs can contact participants directly by phone to schedule an appointment post hospitalization. Furthermore, we will attach notes to the medical charts of hard-to-reach participants at local HIV centers for physicians to give to their patients when they are seen for medical visits. The note will provide the study team phone number and ask participants to call the study team to schedule their next study visit.

Phone interviews will be offered to participants who were not able to make it to FSPSMU for an in-person visit.

Transportation will be arranged (i.e., a social taxi or Uber) for participants who are unable to come to First St. Petersburg Pavlov State Medical University due to a lack of available transportation.

6. ASSESSMENTS

6.1 BASELINE ASSESSMENT

The baseline assessment will be conducted immediately following the screening, informed consent, and blood draw. Assessment will be interviewer-administered with the exception of sections deemed to ask sensitive questions, which will be self-administered by the participant.

Participants will be assessed as part of this study using validated interview instruments covering the following topics:

- Demographics (marital status, education, employment, individual Income/security, date of birth, spouse HIV status, living situation, military service, arrest history, incarceration history)
- Alcohol use by the Timeline Followback (TLFB) calendar method with a 30-day assessment
  - Participants will also be provided with a calendar to record daily alcohol and cigarette use. They will be asked to complete this calendar at home and bring it back to all subsequent study visits. The calendar will be used as an aid by the RA when completing the TLFB with the participant.
- Alcohol use disorders using the Alcohol Use Disorders Identification Test (AUDIT)
- Alcohol dependent using DSM-5 criteria
- Alcohol consequences by the Short Inventory of Problems (SIP) for alcohol use
- Alcohol craving by the Penn Alcohol Craving Scale (PACS)
- Readiness to quit alcohol using Labrie et al.'s readiness to change ruler
- Cigarette use by the Timeline Followback (TLFB) calendar method with a 30-day assessment

- Nicotine dependence by the Fagerstrom Test
- Other tobacco use by adapted Population Assessment of Tobacco Health (PATH Study) questions
- Tobacco craving by the Questionnaire on Smoking Urges (QSU-Brief)
- Readiness to quit tobacco using the Connect to Quit readiness to quit ladder
- Smoking quit attempts
- HIV risk categories, using adapted questions from the American Red Cross
- Depressive symptoms through the Center for Epidemiologic Studies Depression Scale (CES-D)
- Anxiety by the Generalized Anxiety Disorder 7-item Scale (GAD-7)
- Trauma adapted from the revised Conflict Tactics Scales (CTS2) and World Health Organization. Composite International Diagnostic Interview (CIDI): Version 2.1
- HIV/HCV testing and treatment
- ART use and adherence, using modified Adult AIDS Clinical Trial Group (AACTG) ART adherence questions
- Opportunistic infections, using questions adapted from the HIV Cost and Services Utilization Study (HCSUS)
- Co-morbidities by an adapted Veterans Aging Cohort Study patient questionnaire
- Brief chronic pain via the Brief Chronic Pain Questionnaire
- HIV symptoms through a validated HIV Symptom Index from the NIAID Adult AIDS Clinical Trials Group
- Smoking-Related Symptoms via the 7-item Respiratory Index assessing Smoking-Related Respiratory Symptom Severity
- Reproductive health
- Falls using the ACTG 5322 Fall History Questionnaire
- TB testing and treatment
- Healthcare utilization using items from the Form 90 Alcohol Intake Revised/Economic Development (AIR/ED) used in COMBINE
- Prescription and non-prescription medication use
- Brief Pain Inventory using a modified Short Form of the Russian Brief Pain Inventory
- Drug use by an adapted version of the Risk Behavior Survey
- 24 Hour Activities through 6 questions developed by Matthew Freiberg (PI)
- Social support using a modified version of the Duke University-University of North Carolina Functional Support Questionnaire
- General health, quality of life, and cognitive function by the Veterans RAND 12-Item Health Survey (VR-12) and the Medical Outcomes Study HIV Health Survey (MOS-HIV)

6.1.A. OBSERVATIONAL COHORT BASELINE ASSESSMENT

The observational cohort baseline assessment will be the same as the St PETER trial baseline assessment.

6.2 FOLLOW UP ASSESSMENTS

Content of assessments administered at the 1-, 3-, 6-, and 12- month visits will be subsets of the baseline assessment. In addition, questions about smartphone usage will be asked only at the 12-month assessment. Questions about COVID-19 will only be asked at one time point, either the 6 or 12-month assessment. Please see table of study questionnaires at the end of this section.

6.2.A. MEDICATION VISITS ASSESSMENTS

Medication adherence and symptoms will be assessed during the six medication check-ins (in person at weeks 2 and 8, and via phone at weeks 1, 3, 6, and 10).

6.2.B. OBSERVATIONAL COHORT ASSESSMENTS

The observational cohort 12-month assessment will include all the same sections as the St PETER 12-month assessment. Russia ARCH cohort participants will have the opportunity to be re-screened for the St PETER trial at the time of their 12-month assessment.

	Study Time Point					
Administered Assessment	Baseline	Medication Check-Ins	1-Month	3-Month	6-Month	12-Month
Demographics	X					X
HIV/HCV Testing and Treatment	X					X
ART Use and Adherence	X				X	X
Opportunistic Infections (ever)	X					X
Healthcare Utilization (3mo timeframe)	X				X	X
Co-Morbidities (ever)	X					X
Smoking-related symptoms	X		X	X	X	X
Brief Pain Inventory	X				X	X
Brief Chronic Pain Questionnaire	X				X	X
Medications	X		X	X	X	X
HIV Symptom Index (1 mo timeframe)	X					X
Falls	X					X
TB testing and treatment	X					X
HIV Risk Categories	X					
Depressive Symptoms (CES-D) (past week)	X		X	X	X	X

Anxiety (GAD-7)	X		X	X	X	X
DSM-5 Alcohol Use Disorder	X					X
Alcohol Use Disorders Identification Test (AUDIT) (need to adjust timeframe)	X				X	X
SIP	X		X	X	X	X
30-day Alcohol Timeline Followback (TLFB)	X		X	X	X	X
Penn Alcohol Craving Scale	X		X	X	X	X
Readiness to Quit Alcohol	X		X	X	X	X
Tobacco Use (Fagerström)	X		X	X	X	X
30-Day Cigarette Use Timeline Followback (TLFB)	X		X	X	X	X
Other tobacco use	X		X	X	X	X
Tobacco Craving (QSU)	X		X	X	X	X
Past smoking quit attempts	X				X	X
Readiness to Quit Tobacco	X		X	X	X	X
Drug Use (modified RBS) & Overdose & Opioid Craving VAS	X		X	X	X	X
24 Hour Activities	X		X	X	X	X
Social Support Scale (4 weeks)	X					X
Reproductive Health	X					X
VR-12 Health Survey & MOS HIV	X					X
Trauma	X					X
Baseline Symptom Monitoring	X					
Medication Adherence		X	X	X		
Medication Side Effect Checklist		X	X	X		
Medication satisfaction			X	X		
Blinding questions			X	X		
Smartphone questions						X
COVID-19 questions					X	X

7. PARTICIPANT SAFETY

Participant safety will be monitored in person in weeks 2, 4, 8, and 12 and over the phone in weeks 1, 3, 6, and 10 and more frequently, if necessary.

7.1. SPECIFICATION OF SAFETY PARAMETERS

An **Adverse Event (AE)** is defined as any untoward or unfavorable medical occurrence in a human subject, including any abnormal sign (for example, abnormal physical exam or laboratory finding), symptom, or disease, temporally associated with the subject's participation in the research, whether or not considered related to the subject's participation in the research.

An AE can therefore be any new sign, reaction, symptom, event, disease or a worsening in frequency or severity of a preexisting condition that occurs during the course of the study.

Stable chronic conditions that were present prior to study entry and do not worsen are not considered AEs.

SERIOUS Adverse Event (SAE) – for an event to be defined as serious, it will be Grade 1-6 below. Grade 0 would be "not serious".

Grade (1) results in death;

Grade (2) is life-threatening (places the subject at immediate risk of death from the event as it occurred);

Grade (3) results in inpatient hospitalization or prolongation of existing hospitalization;

Grade (4) results in a persistent or significant disability/incapacity;

Grade (5) results in a congenital anomaly/birth defect; or

Grade (6) based upon appropriate medical judgment, may jeopardize the subject's health and may require medical or surgical intervention to prevent one of the other outcomes listed in this definition (examples of such events include allergic bronchospasm requiring intensive treatment in the emergency room or at home, blood dyscrasias or convulsions that do not result in inpatient hospitalization, or the development of drug dependency or drug abuse).

Unanticipated Problem (UP) – for an event to be an Unanticipated Problem it must

- be unexpected AND
- be related or possibly related to participation in the research AND
- suggest that the research places subjects or others at a greater risk of harm (including physical, psychological, economic, or social harm) than was previously known or recognized. OR meet the definition of SERIOUS

Suspected Adverse Drug Reaction – Any adverse event for which there is a reasonable possibility that the drug caused the adverse event. Reasonable possibility means there is evidence to suspect a causal relationship. It is considered unexpected if it is not consistent with the risk information described in the general investigational plan. A suspected adverse drug reaction will be defined as a recorded adverse event that is unexpected and deemed to be possibly, probably, or definitely related to the study drug.

7.2 THE METHODS AND TIME FOR ASSESSING, RECORDING, AND ANALYZING SAFETY PARAMETERS

- Participant symptoms will be assessed at baseline to document any chronic conditions or symptoms that existed prior to introduction of study medication. These will be documented on the Baseline AE log. This list will be reviewed and compared to reported events throughout the study. *If the participant reports the same ongoing symptom (same severity) during subsequent visits, the symptom should not be recorded as an Adverse Event (AE). If the event is new (not previously reported) or worsened, as determined by RA, then the AE should be reported.*

- During each scheduled visit, the RA will ask the participant how he or she feels and review the list of symptoms of concern (starting with the symptoms recorded at the previous visit). Any event that meets the above criteria for an AE/SAE/UP must be recorded. In the case of unresolved AEs, clinical staff will update the AE log with any follow-up information that is gathered during their investigation.

- The site will receive the results of all blood work that is performed on study participants from the designated lab. If the lab results meet the criteria described in the protocol as an AE and are considered clinically significant by the site clinician then an AE will be recorded. **Please see Box 1.**

    - Participants will be alerted of abnormal lab results and will receive a recommendation to see their local provider. All abnormal lab results obtained at the baseline visit will be listed on the Baseline AE log. During follow-up visits, abnormal lab results will be listed as an AE *only if* the abnormal lab results:
        - Develop at follow-up (i.e., were not previously recorded at baseline).
        - Worsen in severity compared to what was previously recorded at baseline.
        - Or are considered clinically significant.
        - If baseline creatinine levels are indicative of reduced kidney function (< 30 cc/min), study investigators will reduce the dose of varenicline by 50%.
    - All AEs will be assessed to determine if they meet criteria for a Serious Adverse Event (SAE). If the AE is serious, then the SAE form must be completed and appropriate reporting measures followed (see below). Investigators are encouraged to consult with the US team, if they are uncertain how to classify an event.
    - The list of subject's current medications will be reviewed and updated at every study visit, starting at baseline.
    - If an event is discovered outside of the scheduled study visits, it must still be recorded accordingly.
    - Action Taken will be determined by the RAs for all AEs that are Mild and Moderate (unless specified below) and by the Site PI for SAEs and AEs that are severe, life-threatening or fatal.

Box 1. Abnormal Lab Results

	A. Normal Lab Values
**Cholesterol (total)**	to 5.2 mmol/L
**HDL**	M: >1.45 mmol/l; F: >1.68 mmol/l
**HS CRP**	to 5 mg/l
**CD4**	28.2 – 62.8 %
**Hemoglobin**	M: 130-160 g/l;<br>F: 120-140 g/l
**Platelet**	M: 180-320 x 10*9 /l;<br>F: 180-320 x 10*9 /l
**eGFR (Crea)**	M: to 120 mcmol/l<br>F: to 110 mcmol/l
**HCV Ab**	negative
**AST**	M: to 38 units/L F: to 32 units/L
**ALT**	M: to 41 units/L F: to 31 units/L

7.3. PROCEDURES FOR ELICITING REPORTS OF AND FOR RECORDING AND REPORTING ADVERSE EVENT AND INTERCURRENT ILLNESSES

For any reported side effect: While with the participant, study personnel will listen, identify, and document the symptoms. The following symptoms will be assessed at baseline and during medication check-ins and study visits while participant is taking the study medication. All events will be documented on the Symptom Checklist and on AE forms.

Symptom
Agitation and/or Irritability
Anger
Depressed mood
Anxiety (includes nervousness and panic attacks)
Restlessness
Insomnia and/or other sleep problems
Abnormal dreams and/or nightmares
Headaches

Dizziness	
Nausea and/or vomiting	
Fatigue	
Hiccups	
Cough	
Throat irritation	

Symptoms at the severity level listed below must be reported to the Site PI immediately during study visit or within 24 hours to assess the need for study medication dosing changes.

**Side Effect Checklist**	
Agitation and/or Irritability	Severe
Anger	Severe
Depressed mood	Moderate<br>Severe
Anxiety (includes nervousness and panic attacks)	Moderate<br>Severe
Headache	Severe
Dizziness	Severe
Nausea and/or vomiting	Severe
**Open Ended Responses** *(this is not an exhaustive list)*	
Allergic reaction	Severe
Asthma	Severe
Blood pressure increase	Severe
Cancer	Any reporting
Trouble concentrating	Severe
Confusion	Severe
Death	Any reporting
Emergency Room Visit	Any reporting
Fainting	Mild<br>Moderate<br>Severe
Heart Attack/MI	Any reporting
Hospitalization	Any reporting
Neurological event<br>Stroke/TIA, etc	Mild<br>Moderate<br>Severe
Psychiatric event<br>(not related to suicide)	Moderate<br>Severe
Racing thoughts	Severe
Seizure	Mild<br>Moderate<br>Severe

Shortness of Breath	Moderate<br>Severe

7.3.A. Other Events

- Other events may or may not be associated with study drug use, but will be recorded, AE form completed and the Site PI will be notified immediately to address the report.
- The Site PI will evaluate the reported symptoms using clinical judgment to determine if they are related to the study and if study medication should be adjusted or ceased.
- The Site PI determine that study medications should be ceased, study personnel will attempt to contact the participant as soon as possible.
- If applicable, staff can advise participants to contact their physician immediately or call emergency services.
- Participants will receive a card to provide to medical staff in the case of a hospitalization or emergency stating that they are involved in a research study and are randomized to one of three active study medications.

7.3. B. Participant Advice

- For certain, relatively common side effects, such as mild-moderate gastrointestinal (GI) problems and moderate sleep problems RA will provide the following advice:
  - For moderate GI problems, RA will remind the participant that the side effect typically gets better within 2-3 weeks following initiation of medication and that they should take the medication after eating.
  - For moderate sleep problems, RA will remind the participant that the side effect typically gets better within 2-3 weeks following initiation of medication and that they should be sure to take their first dose in the early AM so that they can take the second dose in the afternoon.
- For participants who normally drink caffeine or take caffeine pills, make them aware that chemicals (polyaromatic hydrocardbons) in cigarette smoke actually metabolize caffeine, so generally smokers “need” more caffeine when they are smoking at their usual rate. However, when they cut down on smoking or quit altogether, but keep taking in the same amount of caffeine, they may feel jittery, irritable, nauseated, etc, due to higher-than-usual blood levels of caffeine. At the first visit, please suggest that participants reduce caffeine intake by half as they are cutting down or quitting smoking; then they can adjust caffeine up or down from there as tolerated.

7.3. C. Adverse Event Reporting

The following information should be present to complete AE and SAE forms during the initial report (on the day of finding out about the event):

- Description of the event
- Date of onset and resolution (if known)
- Severity – based on established criteria: https://evs.nci.nih.gov/ftp1/CTCAE/CTCAE_4.03_2010-06-14_QuickReference_5x7.pdf * See Box 2

Box 2. Guidelines for Severity Grades

*Research assessor will refer to the guide for unique clinical descriptions of severity for each AE, which will follow the general guideline below:

- Grade 1 Mild; asymptomatic or mild symptoms; clinical or diagnostic observations only intervention not indicated.
- Grade 2 Moderate; minimal, local or noninvasive intervention indicated; limiting age-appropriate instrumental activities ADL*.
- Grade 3 Severe or medically significant but not immediately life-threatening; hospitalization or prolongation of hospitalization indicated; disabling; limiting self care ADL**.
- Grade 4 Life-threatening consequences; urgent intervention indicated.
- Grade 5 Death related to AE.

Not all Grades are appropriate for all AEs. Therefore, some AEs are listed with fewer than five options for Grade selection. Grade 5 (Death) is not appropriate for some AEs and therefore is not an option.

Activities of Daily Living (ADL)

*Instrumental ADL refer to preparing meals, shopping for groceries or clothes, using the telephone, managing money, etc.
**Self care ADL refer to bathing, dressing and undressing, feeding self, using the toilet, taking medications, and not bedridden.

- Assessment of expectedness (is the event anticipated in terms of nature, severity, or frequency) given (a) the research procedures that are described in the IRB protocol and informed consent document; and (b) the characteristics of the subject population being studied
- Assessment of relatedness to study drug
- Any actions taken

  Following the initial report, additional information may need to be gathered to complete the AE and SAE forms and to evaluate the event for relatedness. This process may include obtaining hospital discharge reports, physician records, autopsy records or any other type of records or information necessary to provide a complete and clear picture of the SAE and events preceding and following the event.

7.3.D. SAE REPORTING

If the SAE is not resolved or stabilized at this time or new information becomes available after the SAE form is completed, the SAE form should be updated as soon as possible. Any changes or updates to the SAE form will need to be re-reviewed and re-authorized by the study clinician.

In some cases, the study clinician may be unsure upon first learning of an SAE whether it is study related and/or expected, because study staff are awaiting more complete medical records. In such cases, the study clinician should make his/her best estimate of relatedness and expectedness, understanding that

these determinations can be updated later. When updating determinations at a later date, the rationale for the change should be included in the SAE narrative.

The site must actively seek information about the SAE until the SAE is resolved, stabilized or until the participant is lost to follow-up and terminated from the study.

<u>To summarize</u>: upon determining an Adverse Event is Serious, the following procedures should be followed:

- The study staff, while meeting/talking with the participant or person providing details on the event, will gather as much information about the event from the participant as possible and complete the appropriate forms.

- The completed AE and SAE forms will be reviewed by key personnel on the Pavlov team (e.g. Site PI). Any relevant clinical documents (labs, physician notes) available at that time will be provided to key personnel on the Pavlov team (e.g. Site PI) within 24 hours of finding out about the event.

- After initial notification, the SAE must be updated with any additional information.

All unanticipated problems must be reported to the US team immediately. The US team will report all UPs to the BUMC IRB and NIAAA within 48 hours of discovering their occurrence.

SAEs and unanticipated events which are considered “at least possibly related” during the treatment and follow-up phases will be reported to NIAAA within 48 hours of knowledge of the SAE.

AEs and SAEs will be reported to the URBAN ARCH Data Safety Monitoring Board every six months and as needed.

<u>BUMC Reporting Guidelines:</u>

The Principal Investigator at BMC/BU Medical Campus will report Unanticipated Problems and Adverse Events to the BMC/BU Medical Center IRB in accordance with IRB policies:

- Unanticipated Problems involving a fatal or life-threatening event will be reported to the IRB within 2 days of the investigator learning of the event.
- Unanticipated Problems not involving a fatal or life-threatening event will be reported to the IRB within 7 days of the investigator learning of the event.
- Adverse Events (including Serious Adverse Events) will be reported in summary at the time of continuing review, along with a statement that the pattern of adverse events, in total, does not suggest that the research places subjects or others at a greater risk of harm than was previously known.

<u>Pavlov Reporting Guidelines:</u>

What Event is Reported	When is Event Reported

Fatal or life-threatening unexpected, suspected serious adverse reactions	Within 7 calendar days of initial receipt of information
Non-fatal, non-life-threatening unexpected, suspected serious adverse reactions	Within 15 calendar days of initial receipt of information
AEs and UPs	On a quarterly basis

7.4. THE TYPE AND DURATION OF THE FOLLOW-UP OF SUBJECTS AFTER ADVERSE EVENTS

All adverse events (including serious adverse events) will be followed until the event is resolved, stabilized, or until the end of individual's participation in the study.

The Site PI will determine a follow up plan on a case-by-case basis based on their clinical judgment. After the 3-month visit, ongoing adverse events will be followed monthly until the event is resolved or until the end of individual's participation in the study.

All baseline events (symptoms/conditions that existed prior to participant's enrollment in the study) will be followed at each study visit until the event is resolved or until the end of individual's participation in the study.

7.5. UNBLINDING PROTOCOL

Participants may be unblinded if there is an urgent medical need, as determined by the clinician evaluating the participant. If a participant is unblinded, study medication may be discontinued.

The following are examples of events that may result in emergency unblinding:

-An SAE occurs that is thought to be most likely or definitely related to the study drug.

-An AE or SAE occurs and the clinician treating the patient concludes that knowledge of the treatment arm is necessary to determine the therapy provided to the patient.

-The study drug is accidentally ingested by a child.

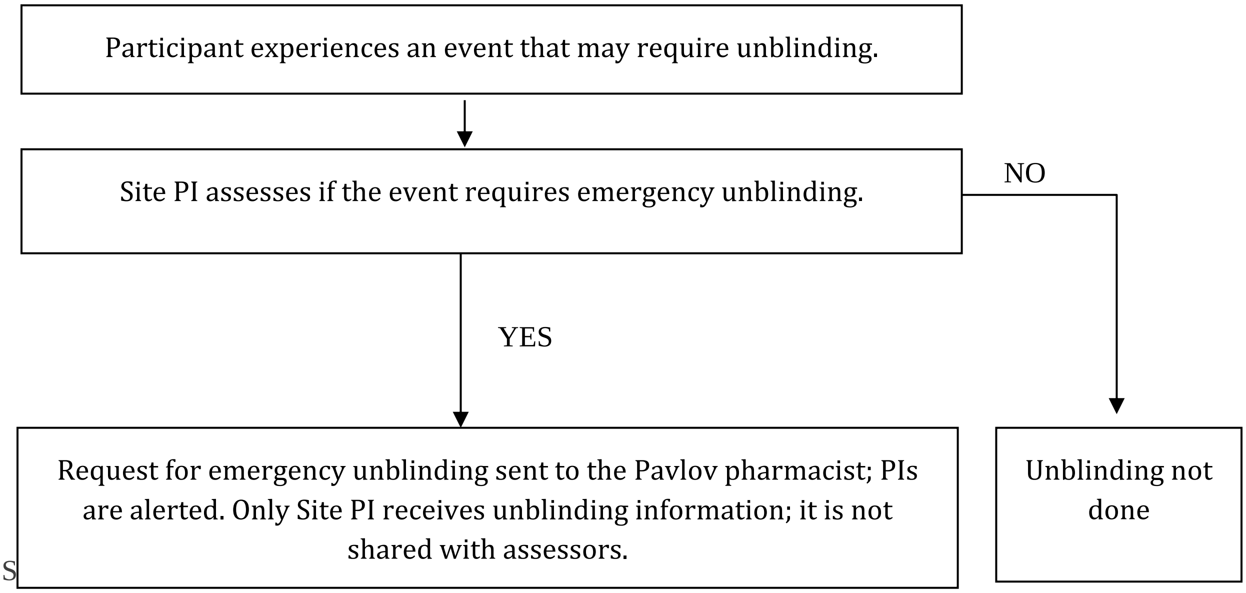

7.6. DATA SAFETY AND MONITORING BOARD

The Data and Safety Monitoring Board (DSMB) will monitor the URBAN ARCH Cohort studies (i.e., Uganda, Russia, Boston) and Intervention trials (i.e., ZINC, ST PETER, ADEPT-TB). The DSMB will act in an advisory capacity to the study PIs and NIAAA to monitor participant safety, data quality and evaluate the progress of the studies being conducted under the URBAN ARCH consortium funded by the National Institute on Alcohol Abuse and Alcoholism.

The DSMB is responsible for ensuring subject safety (by reviewing blinded and unblinded safety data on a regular basis and assessing the safety of study procedures) and for monitoring the overall conduct of the studies. The focus will be on the clinical drug trials as that is where the greatest potential risk lies; however, the DSMB will still monitor enrollment, follow up and adverse events of the cohort studies. No interim efficacy analyses will be conducted for any of the studies.

The DSMB is an independent group advisory to the PIs and the NIAAA, and is required to provide recommendations about starting, continuing, temporarily suspending the trial until certain conditions are met, and stopping the studies. In addition, the DSMB is asked to make recommendations, as appropriate, about:

- Benefit/risk ratio of procedures and participant burden
- Selection, recruitment, and retention of participants
- Protocol violations and adherence to protocol requirements
- Completeness, quality, and analysis of measurements
- Amendments to the study protocol and consent forms
- Participant safety
- Notification of and referral for abnormal findings

This DSMB will meet every six months. In cases where Institutional Review Boards or the NIH require more frequent monitoring, procedures will be put into place to conform to those requirements. An agenda will be provided detailing the studies to be discussed. It is estimated that the meeting will be scheduled for 1.5 hours. Each protocol and data review meeting will consist of two sessions: Open Session and Closed Session. Communication in the interim will be as needed. Unscheduled meetings can be requested by any party with the responsibility of overseeing the study. Requests can be made to the DSMB Chair, PIs, or NIAAA officials. The Chair, in collaboration with the Admin Core or NIAAA, will schedule any unplanned meetings.

8. DATA MANAGEMENT

8.1 DATA COLLECTION

All study data will be captured electronically on netbooks via a secure, web-based data capture system with the exception of: TLFB data, which will be collected on paper calendars.

8.2 QUALITY CONTROL PROCESS

Quality control measures will include: detailed and unambiguous specifications for completion of data forms, including rules for coding skipped questions and missing data, training of study staff responsible for data collection and built-in validation rules, error checks, question skips for electronic data capture, and computer algorithms to check for out-of-range codes and internal inconsistencies. All data, regardless of capture method, will be converted to SAS datasets and reviewed for logic, skip patterns, response ranges, out-of-range codes, and internal inconsistencies. The RAs will be queried monthly regarding any noted inconsistencies.

8.3 DATA SECURITY AND CONFIDENTIALITY

Screening forms and most other research paperwork will not include the participant's name; instead, a unique ID will be assigned to each person screened, and another number assigned to those who enrolled. Any documents with identifiable participant data will only be accessible to the Russian Co-Investigators, the project manager, and the RAs who recruit and follow participants.

Tracking information will be kept similarly. Computer data will be password protected, and accessible only to research associates needing the information for follow-up purposes.

The BDM Core of the ARCH Consortium will design, develop and maintain the electronic data collection forms, participant and data tracking, and underlying SQL database systems, and implement procedures for data quality control, including multiple checks for entered data. Electronic data collection forms will be designed to read easily, have clear instructions, preprogrammed skip patterns, real-time range checks and internal logic to minimize missing data and result in "cleaner" data at capture. The website and accompanying database will be located on secure, password-protected servers, behind the BU firewalls. The BDM Core has access to two Unix servers, including a Linux Beowulf cluster currently configured with 118 CPUs, as well as an SMP Linux server with 4 x Six-Core AMD Opteron processors (a total of 24 cores x 2.4 GHz each), 64 GB of RAM, and 6 TB (4TB usable) storage capacity. Additionally, the Data Coordinating Center (DCC) has three dedicated servers, all of which are dual processors with 150 gigabytes for data storage: an SQL database server; a server used for Web site development and management, running Internet Information Server for web page hosting; and a server used for web development pre-production testing environment. The web and database servers will use Secure Socket Layering (SSL) to ensure data security and confidentiality. Two fax servers, an additional server, and a flatbed scanner comprise the Teleform® system. Servers incorporate RAID hard drives for data redundancy. A separate web server dedicated for Cold Fusion applications is also available.

FORM	STUDY VISIT												
	Phone Screen	Screen & Baseline Visit		1 week call	2 week visit	3 week call	1 mo visit	Calls in weeks 6 & 10	2 mo visit	3 mo visit	6 mo visit	12 mo visit	As Nee ded
		Screen	Base										

8.4 WEB SYSTEMS

The study will use two web systems: a computerized tracking system and REDCap. The computerized tracking system will contain all participant tracking details. This system will be web-based, allowing multiple users to access the system. REDCap is a secure web application for building and managing online surveys and databases and will be used for screening and assessments purposes. Study forms will be completed according to the schedule below.

Based on information entered into web-based tracking system by assessors, the DCC will run reports to generate information on scheduled visits and reminder calls. These reports will be updated twice daily at 7:00am EDT (14:00 MSK) and 7:00pm EDT (2:00 MSK).

			line										
Pre-Screener	X												
Screener		X											
Consent and enrollment form		X											
Contact info			X	X	X	X	X	X	X	X	X	X	X
Phlebotomy form			X				X			X	X	X	
Lab Processing form			X				X			X	X	X	
Full assessment			X				X			X	X	X	
Contact log													X
Baseline Event Form			X										X
Symptom Monitoring Form				X	X	X	X	X	X	X			
Medication visit checklist			X		X		X		X				
Medication collection (Paper)			X	X	X	X	X	X	X	X			X
Baseline tracking form			X										
Short assessment (adherence)				X	X	X		X	X				
Follow-up tracking form			X	X	X	X	X	X	X	X	X	X	
Participant tracking overview													X
Study conclusion form													X
AE/SAE form (paper and Web)													X
Incarceration form													X
All forms are electronic, unless indicated otherwise													

9. STATISTICAL ANALYSIS

This study will use an intent-to-treat analysis that includes all participants according to their randomized assignment. Descriptive statistics will be calculated for variables at baseline and each follow-up time to assess whether there appear to be any differences across treatment arms.

Our Specific Aims are to compare the effects of varenicline, cytisine, and NRT among HIV+ heavy drinkers and current daily smokers for the following outcomes:
1. % Heavy drinking days in past month (self-report, primary outcome) and alcohol craving (self-report);
2. Cigarettes per day (past week, self-report); 7-day point prevalence abstinence (biochemically verified);
3. Inflammation (hsCRP, IL-6), CHD risk (Reynolds risk score), and mortality risk (VACS index).

Self-reported alcohol and tobacco outcomes (Aims 1-2) will be collected at 1, 3, 6, 12 months. Aim 3 outcomes will be collected at 3 months.

The 3 pairwise comparisons of interest are:

1. varenicline vs. NRT (Arms 1 vs 2)
2. cytisine vs. NRT (Arms 3 vs. 4)
3. varenicline vs. cytisine (Arms 1 vs. 3)

9.1 PRIMARY ANALYSES

The main analysis evaluating the impact of each intervention on the primary study outcome (i.e., the continuous variable % HDD at 3 months) will use multiple regression models. The models will include indicator variables to represent study arm. We will test 3 pairwise comparisons (i.e., varenicline + NRT placebo vs. NRT + varenicline placebo; cytisine + NRT placebo vs. NRT + cytisine placebo; varenicline vs. cytisine) and will adjust for the multiple comparisons using the Hochberg sequential test procedure. To improve efficiency, the regression analyses will control for stratification factors: alcohol consumption (≥ 3 vs. < 3 heavy drinking days in the past week according to NIAAA risky drinking criteria, assessed via the first 7 days of the Timeline Follow Back), average daily cigarettes (≤ 1 vs. >1 pack per day, assessed via question 5 of the Tobacco Use section), and current ART use [yes vs. no]. If the data are normally distributed, multiple linear regression models will be used. However, if the distribution of % HDD is skewed, transformations of the data will be performed (e.g., log transformation). If an appropriate transformation is not identified, a median regression model will be used. Continuous outcomes (e.g., cigarettes per day in the past week, primary outcome Aim 2; biomarkers of inflammation, Reynold's risk score, VACS Index, primary and secondary outcomes for Aim 3) will be analyzed using the same approach described above. If count data (e.g., number of cigarettes) are not approximately normally distributed, they will be analyzed using Poisson or negative binomial (to account for overdispersion) regression models. Binary outcomes (e.g., smoking cessation, secondary outcome Aim 2) will be analyzed using logistic regression models. Models will control for stratification factors as described above for % HDD. A secondary analysis will be conducted using a per protocol approach that includes only those participants who were adherent to their assigned intervention (i.e., taking study medications or placebos 80% of the time), as determined by pill count.

9.2 ADDITIONAL EXPLORATORY ANALYSES

Intervention effects over time

The main analyses will focus on % HDD at 3months, our primary outcome. Primary analyses of smoking and inflammation are also assessed at 3 months. However, given the repeated measures of our alcohol and smoking variables (i.e., 1, 3, 6, and 12 months), additional analyses using longitudinal regression models will be used to incorporate repeated measures for each outcome (alcohol and smoking) in the same model and will test for possible intervention by time interactions (e.g., does the effect of varenicline or cytisine change over time). For continuous outcomes (e.g., % HDD), we will use generalized linear mixed effects models that include subject-specific random intercepts and slopes to account for the correlation due to having repeated observations from each subject. For dichotomous outcomes, we will

use generalized estimating equations (GEE), with an independence working correlation matrix, a logit link; standard errors will be based on the empirical-sandwich estimator. Secondary analyses will also be performed using mixed effects logistic regression models for correlated binary outcomes. The GEE approach compares population averages over time and the mixed effects approach models subject-specific effects; the latter is computationally more intensive and requires more modeling assumptions but may be more powerful if the assumptions are correct.

Effect Modification

Because heavy alcohol use, depressive symptoms and opioid use are common in Russia ARCH we will perform additional analyses to explore whether baseline alcohol consumption (≥15 HDD in past month), depressive symptoms (measured by CES-D score ≥16) or opioid use (self-reported history of opioid use in Russia ARCH assessment) are potential effect modifiers of the interventions. We will fit separate models that include 2-way interactions between randomization group and each potential effect modifier. If an interaction is significant, subsequent stratified analyses will be conducted to evaluate the effect of varenicline/cytisine by alcohol, depressive symptoms, or opioid group. Exploratory analyses will also examine intervention effects by sex to describe and estimate effects within each subgroup.

Mediation

We will explore potential mechanisms through which the interventions are mediated. For example, the interventions may lead to a reduction in alcohol use, which in turn leads to reduced inflammation, CHD risk, and mortality risk. To assess potential mechanisms, we will use the approach described by Baron and Kenny. To assess whether alcohol use (% HDD) is in the causal pathway between the intervention and biomarkers of inflammation (hsCRP and IL-6), we will evaluate whether the following conditions hold: a) alcohol use is related to inflammation; b) the intervention is related to alcohol use; c) the intervention is related to biomarkers of inflammation; and d) The effect of the interventions on inflammation reduces appreciably when alcohol use is added to the model. We will conduct confirmatory analyses using the counterfactual framework, an approach that allows potential interactions between the interventions and mediators and derives direct and indirect effects with different types of outcomes (e.g., dichotomous). In addition to alcohol, we will also assess whether smoking is in the causal pathway between the interventions and markers of inflammation, CHD risk, and mortality risk.

10. STAFF TRAINING

All study staff will be trained on the study protocol, including administration of study medication, behavioral counseling, symptom monitoring, and participant assessment prior to initiation of recruitment and enrollment. Training will take place in-person in St. Petersburg and via webinars.

REFERENCES

1. Freiberg MS, Chang CC, Kuller LH, Skanderson M, Lowy E, Kraemer KL, Butt AA, Bidwell Goetz M, Leaf D, Oursler KA, Rimland D, Rodriguez Barradas M, Brown S, Gibert C, McGinnis K, Crothers K, Sico J, Crane H, Warner A, Gottlieb S, Gottdiener J, Tracy RP, Budoff M, Watson C, Armah KA, Doebler D, Bryant K, Justice AC. HIV infection and the risk of acute myocardial infarction. *JAMA Intern Med*. 2013;173(8):614-622.

2. Kuller L,Group SS. Elevated levels of interleukin-6 and d-dimer are associated with an increased risk of death in patients with HIV. *Conference on Retroviruses and Opportunistic Infections.* 2008; 15th Conference on Retroviruses and Opportunistic Infections; Boston, MA.

3. Kuller LH, Tracy R, Belloso W, De Wit S, Drummond F, Lane HC, Ledergerber B, Lundgren J, Neuhaus J, Nixon D, Paton NI, Neaton JD, INSIGHT SMART Study Group. Inflammatory and coagulation biomarkers and mortality in patients with HIV infection. *PLoS Med*. 2008;5(10):e203. PMCID: PMC2570418.

4. Baker JV, Neuhaus J, Duprez D, Kuller LH, Tracy R, Belloso WH, De Wit S, Drummond F, Lane HC, Ledergerber B, Lundgren J, Nixon DE, Paton NI, Neaton JD, for the INSIGHT SMART Study Group. Changes in inflammatory and

coagulation biomarkers: A randomized comparison of immediate versus deferred antiretroviral therapy in patients With HIV infection. *J Acquir Immune Defic Syndr*. 2011;56(1):36-43.

5. Triant VA, Meigs JB, Grinspoon SK. Association of C-reactive protein and HIV infection with acute myocardial infarction. *J Acquir Immune Defic Syndr*. 2009;51(3):268-273. PMCID: PMC2763381.

6. Niaura R, Shadel WG, Morrow K, Tashima K, Flanigan T, Abrams DB. Human immunodeficiency virus infection, AIDS, and smoking cessation: the time is now. *Clin Infect Dis*. 2000;31(3):808-812.

7. Armah KA, McGinnis K, Baker J, Gibert C, Butt AA, Bryant KJ, Goetz M, Tracy R, Oursler KK, Rimland D, Crothers K, Rodriguez-Barradas M, Crystal S, Gordon A, Kraemer K, Brown S, Gerschenson M, Leaf DA, Deeks SG, Rinaldo C, Kuller LH, Justice A, Freiberg M. HIV status, burden of comorbid disease, and biomarkers of inflammation, altered coagulation, and monocyte activation. *Clin Infect Dis*. 2012;55(1):126-136.

8. Lifson AR, Neuhaus J, Arribas JR, van den Berg-Wolf M, Labriola AM, Read TR, INSIGHT SMART Study Group. Smoking-related health risks among persons with HIV in the Strategies for Management of Antiretroviral Therapy clinical trial. *Am J Public Health*. 2010;100(10):1896-1903. PMCID: PMC2936972.

9. Helleberg M, Afzal S, Kronborg G, Larsen CS, Pedersen G, Pedersen C, Gerstoft J, Nordestgaard BG, Obel N. Mortality attributable to smoking among HIV-1-infected individuals: a nationwide, population-based cohort study. *Clin Infect Dis*. 2013;56(5):727-734.

10. Cahill K, Stead LF, Lancaster T. Nicotine receptor partial agonists for smoking cessation. *Cochrane Database Syst Rev*. 2012;4:CD006103.

11. West R, Zatonski W, Cedzynska M, Lewandowska D, Pazik J, Aveyard P, Stapleton J. Placebo-controlled trial of cytisine for smoking cessation. *N Engl J Med*. 2011;365(13):1193-1200.

12. Walker N, Howe C, Glover M, McRobbie H, Barnes J, Nosa V, Parag V, Bassett B, Bullen C. Cytisine versus nicotine for smoking cessation. *N Engl J Med*. 2014;371(25):2353-2362.

13. Hajek P, McRobbie H, Myers K. Efficacy of cytisine in helping smokers quit: systematic review and meta-analysis. *Thorax*. 2013;68(11):1037-1042.

14. Jorenby DE, Hays JT, Rigotti NA, Azoulay S, Watsky EJ, Williams KE, Billing CB, Gong J, Reeves KR, Varenicline Phase 3 Study Group. Efficacy of varenicline, an alpha4beta2 nicotinic acetylcholine receptor partial agonist, vs placebo or sustained-release bupropion for smoking cessation: a randomized controlled trial. *JAMA*. 2006;296(1):56-63.

15. Mitchell JM, Teague CH, Kayser AS, Bartlett SE, Fields HL. Varenicline decreases alcohol consumption in heavy-drinking smokers. *Psychopharmacology (Berl)*. 2012;223(3):299-306. PMCID: PMC3438402.

16. Fucito LM, Toll BA, Wu R, Romano DM, Tek E, O'Malley SS. A preliminary investigation of varenicline for heavy drinking smokers. *Psychopharmacology (Berl)*. 2011;215(4):655-663. PMCID: PMC3645986.

17. McKee SA, Harrison EL, O'Malley SS, Krishnan-Sarin S, Shi J, Tetrault JM, Picciotto MR, Petrakis IL, Estevez N, Balchunas E. Varenicline reduces alcohol self-administration in heavy-drinking smokers. *Biol Psychiatry*. 2009;66(2):185-190. PMCID: PMC2863311.

18. Plebani JG, Lynch KG, Rennert L, Pettinati HM, O'Brien CP, Kampman KM. Results from a pilot clinical trial of varenicline for the treatment of alcohol dependence. *Drug Alcohol Depend*. 2013;133(2):754-758. PMCID: PMC3866905.

19. Childs E, Roche DJ, King AC, de Wit H. Varenicline potentiates alcohol-induced negative subjective responses and offsets impaired eye movements. *Alcohol Clin Exp Res*. 2012;36(5):906-914. PMCID: PMC3342420.

20. Meszaros ZS, Abdul-Malak Y, Dimmock JA, Wang D, Ajagbe TO, Batki SL. Varenicline treatment of concurrent alcohol and nicotine dependence in schizophrenia: a randomized, placebo-controlled pilot trial. *J Clin Psychopharmacol*. 2013;33(2):243-247.

21. Sajja RK, Rahman S. Cytisine modulates chronic voluntary ethanol consumption and ethanol-induced striatal up-regulation of DeltaFosB in mice. *Alcohol*. 2013;47(4):299-307.

22. Sajja RK, Rahman S. Neuronal nicotinic receptor ligands modulate chronic nicotine-induced ethanol consumption in C57BL/6J mice. *Pharmacol Biochem Behav*. 2012;102(1):36-43.

23. Bell RL, Eiler BJ,2nd, Cook JB, Rahman S. Nicotinic receptor ligands reduce ethanol intake by high alcohol-drinking HAD-2 rats. *Alcohol*. 2009;43(8):581-592. PMCID: PMC2813038.

24. Petoumenos K, Worm S, Reiss P, de Wit S, d'Arminio Monforte A, Sabin C, Friis-Moller N, Weber R, Mercie P, Pradier C, El-Sadr W, Kirk O, Lundgren J, Law M, D:A:D Study Group. Rates of cardiovascular disease following smoking cessation in patients with HIV infection: results from the D:A:D study(*). *HIV Med*. 2011;12(7):412-421. PMCID: PMC3070963.

25. Sobell LC, Sobell MB. *Alcohol Timeline Followback (TLFB) Users' Manual.* Toronto, Canada: Addiction Research Foundation; 1995.

26. Flannery BA, Volpicelli JR, Pettinati HM. Psychometric properties of the Penn Alcohol Craving Scale. *Alcohol Clin Exp Res*. 1999;23(8):1289-1295.

27. SRNT Subcommittee on Biochemical Verification. Biochemical verification of tobacco use and cessation. *Nicotine Tob Res*. 2002;4(2):149-159.

28. Perkins KA, Karelitz JL, Jao NC. Optimal carbon monoxide criteria to confirm 24-hr smoking abstinence. *Nicotine Tob Res*. 2013;15(5):978-982. PMCID: PMC3733388.

29. Justice AC, Freiberg MS, Tracy R, Kuller L, Tate JP, Goetz MB, Fiellin DA, Vanasse GJ, Butt AA, Rodriguez-Barradas MC, Gibert C, Oursler KA, Deeks SG, Bryant K, the VACS Project Team. Does an Index Composed of Clinical Data Reflect Effects of Inflammation, Coagulation, and Monocyte Activation on Mortality Among Those Aging With HIV?. *Clin Infect Dis*. 2012.

30. Justice AC, Modur SP, Tate JP, Althoff KN, Jacobson LP, Gebo KA, Kitahata MM, Horberg MA, Brooks JT, Buchacz K, Rourke SB, Rachlis A, Napravnik S, Eron J, Willig JH, Moore R, Kirk GD, Bosch R, Rodriguez B, Hogg RS, Thorne J, Goedert JJ, Klein M, Gill J, Deeks S, Sterling TR, Anastos K, Gange SJ, NA-ACCORD and VACS Project Teams. Predictive accuracy of the Veterans Aging Cohort Study index for mortality with HIV infection: a North American cross cohort analysis. *J Acquir Immune Defic Syndr*. 2013;62(2):149-163. PMCID: PMC3619393.

31. Litten RZ, Ryan ML, Fertig JB, Falk DE, Johnson B, Dunn KE, Green AI, Pettinati HM, Ciraulo DA, Sarid-Segal O, Kampman K, Brunette MF, Strain EC, Tiouririne NA, Ransom J, Scott C, Stout R, NCIG (National Institute on Alcohol Abuse and Alcoholism Clinical Investigations Group) Study Group. A double-blind, placebo-controlled trial assessing the efficacy of varenicline tartrate for alcohol dependence. *J Addict Med*. 2013;7(4):277-286. PMCID: PMC3914416.

32. National Institute on Alcohol Abuse and Alcoholism. *A Pocket Guide for Alcohol Screening and Brief Intervention: Updated 2005 Edition.* Rockville, MD: National Institutes of Health; 2007.

33. Agency for Healthcare Research and Quality. Five major steps to intervention (the "5 A's"). http://www.ahrq.gov/professionals/clinicians-providers/guidelines-recommendations/tobacco/5steps.html. Accessed December 8, 2015.

34. Carpenter MJ, Hughes JR, Gray KM, Wahlquist AE, Saladin ME, Alberg AJ. Nicotine therapy sampling to induce quit attempts among smokers unmotivated to quit: a randomized clinical trial. *Arch Intern Med*. 2011;171(21):1901-1907.

35. Lindson-Hawley N, Banting M, West R, Michie S, Shinkins B, Aveyard P. Gradual Versus Abrupt Smoking CessationA Randomized, Controlled Noninferiority TrialGradual Versus Abrupt Smoking Cessation. *Ann Intern Med*. 2016;164(9):585-592.

36. Lerman C, Schnoll RA, Hawk LW,Jr, Cinciripini P, George TP, Wileyto EP, Swan GE, Benowitz NL, Heitjan DF, Tyndale RF, PGRN-PNAT Research Group. Use of the nicotine metabolite ratio as a genetically informed biomarker of response to nicotine patch or varenicline for smoking cessation: a randomised, double-blind placebo-controlled trial. *Lancet Respir Med*. 2015;3(2):131-138. PMCID: PMC4480925.

37. Anthenelli RM, Benowitz NL, West R, St Aubin L, McRae T, Lawrence D, Ascher J, Russ C, Krishen A, Evins AE. Neuropsychiatric safety and efficacy of varenicline, bupropion, and nicotine patch in smokers with and without psychiatric disorders (EAGLES): a double-blind, randomised, placebo-controlled clinical trial. *Lancet*. 2016.
