# Supplementary material for: Effectiveness of Varenicline and Cytisine for Alcohol Use Reduction Among People With HIV and Substance Use: A Randomized Clinical Trial
Source: JAMA Netw Open. 2022 Aug 5;5(8):e2225129. doi: 10.1001/jamanetworkopen.2022.25129 (PMC9356316; doi:10.1001/jamanetworkopen.2022.25129)
Supplement: Supplement 2 — eFigure 1. Schedule for Study Medications eTable 1. Imputed Rates of Alcohol and Smoking Abstinence eTable 2. Self-Reported Adherence to Study Medications by Treatment Group and Medication Type eTable 3. Per-Protocol Analysis of Heavy Drinking Days and Smoking Outcomes Among Participants Who Were Adherent to Study Medication eTable 4. Adverse Events eTable 5. Post Hoc Analyses Examining No. of Heavy Drinking Days and Percent With Alcohol Abstinence by Smoking Status at 3, 6, and 12 mo [file jamanetwopen-e2225129-s002.pdf]

Supplemental Online Content

Tindle HA, Freiberg MS, Cheng DM, et al. Effectiveness of varenicline and cytisine for alcohol use reduction among people with HIV and substance use: a randomized clinical trial. *JAMA Netw Open*. 2022;5(8):e2225129. doi:10.1001/jamanetworkopen.2022.25129

eFigure 1. Schedule for Study Medications

eTable 1. Imputed Rates of Alcohol and Smoking Abstinence

eTable 2. Self-Reported Adherence to Study Medications by Treatment Group and Medication Type

eTable 3. Per-Protocol Analysis of Heavy Drinking Days and Smoking Outcomes Among Participants Who Were Adherent to Study Medication

eTable 4. Adverse Events

eTable 5. Post Hoc Analyses Examining No. of Heavy Drinking Days and Percent With Alcohol Abstinence by Smoking Status at 3, 6, and 12 mo

This supplemental material has been provided by the authors to give readers additional information about their work.

© 2022 Tindle HA et al. *JAMA Network Open.*

eFigure 1. Schedule for Study Medications

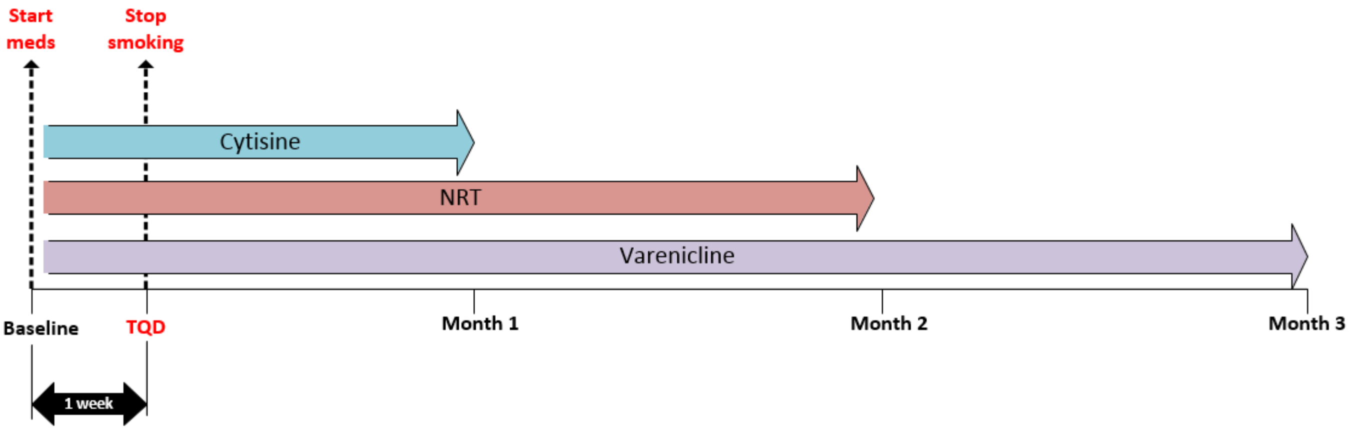

Cytisine followed a traditional 25-day downward titration schedule. Nicotine replacement spray was provided for 8 weeks, and varenicline for 12 weeks. As noted in Figure 1, all participants received one active medication and one placebo medication. At baseline, all participants received brief counseling for smoking and drinking alcohol, began study medication, and were advised to quit smoking on the target quit date (TQD) one week later.

© 2022 Tindle HA et al. *JAMA Network Open.*

eTable 1. Imputed Rates of Alcohol and Smoking Abstinence (N = 400)

Outcome	Varenicline Group[a]		Cytisine Group[a]	
	Group 1 (n=100)	Group 2 (n=99)	Group 3 (m=100)	Group 4 (n=101)
**3 Months**				
Abstinence				
Alcohol[b], No (%)	23 (23.0%)	19 (19.2%)	22 (22.0%)	20 (19.8%)
Smoking[c], No. (%)	24 (24.0%)	13 (13.1%)	26 (26.0%)	22 (21.8%)
**6 Months**				
Smoking abstinence, No. (%)	15 (15.0%)	17 (17.2%)	19 (19.0%)	19 (18.8%)
**12 Months**				
Smoking abstinence, No. (%)	17 (17.0%)	17 (17.2%)	24 (24.0%)	21 (20.8%)

[a] Group 1 received active varenicline and placebo nicotine replacement therapy; group 2 received placebo varenicline and active nicotine replacement therapy; group 3 received active cytisine and placebo nicotine replacement therapy; group 4 received placebo cytisine and active nicotine replacement therapy.
[b] Alcohol abstinence was defined as self-report of no alcohol use in the prior 30 days and biochemically validated with a phosphatidylethanol level of less than 8 ng/ml.
[c] Smoking abstinence was defined a self-reported 7-day point prevalent abstinence that was biochemically validated using a threshold of carbon monoxide level of less than 10 ppm.

© 2022 Tindle HA et al. *JAMA Network Open.*

eTable 2. Self-Reported Adherence to Study Medications by Treatment Group and Medication Type

Adherence	**Overall**[a]	**Arm 1 Active Varenicline + Placebo NRT**	**Arm 2 Placebo Varenicline + Active NRT**	**Arm 3 Active Cytisine + Placebo NRT**	**Arm 4 Placebo Cytisine + Active NRT**	**p-value**
	Participants, No. (%)	Participants, No. (%)	Participants, No. (%)	Participants, No. (%)	Participants, No. (%)	
**Adherence to pills (Yes** [b]**)**	348 (87)	91 (91)	84 (84.8)	86 (86)	87 (86.1)	0.58
**Adherence to spray (Yes** [b]**)**	238 (59.5)	64 (64)	62 (62.6)	57 (57)	55 (54.5)	0.46

[a] The proportion of participants adherent to pills was greater than the proportion of participants adherent to spray (p<0.001).

[b] Participants were considered adherent to study pills if they self-reported taking at least 80% of their assigned study medication. Given differing treatment regimens, the definition of adherence allowed for differences by medication type, i.e., taking at least 80% of assigned medication at 2 of 3 study visits prior to the 1-month visit for cytisine and at 4 of 7 study visits prior to the 3-month visit for varenicline. Participants were considered adherent to study mouth spray if: 1) during weeks 1-4 of treatment they self-reported using at least 8 sprays per day at least 80% of the days in the last week at 2 of 3 study visits; and 2) during weeks 5-8 of treatment if they used the spray at least 80% of times when having an urge to smoke at 1 of 2 study visits prior to the 2-month visit.

© 2022 Tindle HA et al. *JAMA Network Open.*

eTable 3. Per-Protocol Analysis of Heavy Drinking Days and Smoking Outcomes Among Participants Who Were Adherent to Study Medication

Outcome	**Arm 1 vs. Arm 2 Active Varenicline vs. Active NRT** (N=153)		**Arm 3 vs. Arm 4 Active Cytisine vs. Active NRT** (N=141)		**Arm 1 vs. Arm 3 Active Varenicline vs. Active Cytisine** (N=177)	
	**Incidence Rate Ratio (95% CI)**	**p-value**	**Incidence Rate Ratio (95% CI)**	**p-value**	**Incidence Rate Ratio (95% CI)**	**p-value**
Number of Heavy Drinking Days in the past 30 days at 3 months (Primary study outcome)[a]	0.92 (0.44-1.96)	0.83	0.47 (0.21-1.05)	0.20	1.24 (0.60-2.54)	0.83
Cigarettes smoked per day in the past 7 days at 3 months[b]	0.78 (0.44-1.37)	0.77	1.09 (0.60-1.97)	0.77	0.67 (0.40-1.12)	0.38
	**Odds Ratio (95% CI)**	**p-value**	**Odds Ratio (95% CI)**	**p-value**	**Odds Ratio (95% CI)**	**p-value**
Smoking abstinence at 3 months[c]	2.10 (0.85-5.22)	0.33	1.06 (0.45-2.47)	0.93	0.97 (0.47-2.01)	0.93
Smoking abstinence at 6 months	0.82 (0.32-2.08)	0.67	0.72 (0.28-1.81)	0.67	0.80 (0.33-1.90)	0.67

[a] At 3 months, mean (SD) HDD for Arms 1, 2, 3 and 4, respectively: 1.9 (3.6); 2.2 (4.6); 1.5 (3.3); 3.1 (6.3)

[b] At 3 months, mean (SD) cigarettes smoked per day for Arms 1, 2, 3 and 4, respectively: 4.3 (5.6); 5.5 (7.3); 6.3 (7.5); 6.3 (7.6).

[c] Smoking abstinence defined as self-reported 7-day point prevalence abstinence that is biochemically validated using a threshold of carbon monoxide (CO) < 10 ppm. At 3 months, smoking cessation for Arms 1, 2, 3 and 4, respectively: 24.2%; 14.5%; 25.6%; 21.8%. At 6 months, smoking cessation for Arms 1, 2, 3 and 4, respectively: 15.4%; 17.7%; 18.6%; 21.8%.

© 2022 Tindle HA et al. *JAMA Network Open.*

**eTable 4.** Adverse Events												
**Adverse Event**	**Active Varenicline + Placebo NRT**			**Placebo Varenicline + Active NRT**			**Active Cytisine + Placebo NRT**			**Placebo Cytisine + Active NRT**		
**Event Description**	**Mild**	**Moderate**	**Severe**	**Mild**	**Moderate**	**Severe**	**Mild**	**Moderate**	**Severe**	**Mild**	**Moderate**	**Severe**
ABNORMAL ECHO	0	1	0	0	0	0	0	0	0	0	0	0
ACUTE OTITIS	0	0	0	0	0	0	0	0	1	0	0	0
AGITATION AND/OR IRRITABILITY	2	0	0	0	0	0	0	1	0	1	0	0
ANEMIA	0	1	0	0	0	1	0	3	0	0	1	0
ARTERIAL HYPERTENSION	0	1	0	0	0	0	0	1	0	0	0	0
BLOATING	1	0	0	1	0	0	0	0	0	0	0	0
COLD	0	2	0	0	1	0	0	0	0	0	0	0
DEPRESSED MOOD	0	0	0	0	0	0	0	1	0	0	0	0
DIARRHEA	3	0	0	1	0	0	0	0	0	0	0	0
DIZZINESS	0	0	0	2	0	0	0	1	0	0	0	0
DRY MOUTH	1	0	0	0	0	0	0	0	0	0	0	0
DYSPNEA	1	0	0	0	0	0	0	0	0	0	0	0
ELEVATED ALT/AST	0	2	2	0	1	3	0	6	2	0	3	3

© 2022 Tindle HA et al. *JAMA Network Open.*

Adverse Event	Active Varenicline + Placebo NRT			Placebo Varenicline + Active NRT			Active Cytisine + Placebo NRT			Placebo Cytisine + Active NRT		
**Event Description**	**Mild**	**Moderate**	**Severe**	**Mild**	**Moderate**	**Severe**	**Mild**	**Moderate**	**Severe**	**Mild**	**Moderate**	**Severe**
FATIGUE	0	0	0	0	0	0	0	1	0	0	0	0
FEVER	0	1	0	0	0	0	0	0	0	0	1	0
FLATULENCE	1	0	0	0	0	0	0	0	0	0	0	0
FRACTURE OF ARM	0	0	0	0	0	0	0	1	0	0	0	0
GENERALIZED MUSCLE WEAKNESS	0	0	0	0	0	0	0	1	0	0	0	0
HEADACHES	1	0	0	1	0	0	0	2	0	0	0	0
HEPATITIS C	0	0	0	1	0	0	0	0	0	0	0	0
HICCUPS	0	0	0	1	1	0	1	0	0	4	0	0
INSOMNIA AND/OR OTHER SLEEP PROBLEMS	1	0	0	0	0	0	0	1	0	0	0	0
ITCH	1	1	0	0	0	0	0	0	0	0	0	0
METALLIC TASTE IN MOUTH	0	0	0	1	0	0	0	0	0	0	0	0
MYOCARDIAL INFARCTION	0	0	0	0	0	2	0	0	0	0	0	0
NAUSEA	0	0	0	5	2	0	1	1	0	3	1	0
PAIN IN BACK	0	0	0	0	0	0	0	0	0	0	1	0

© 2022 Tindle HA et al. *JAMA Network Open.*

Adverse Event	Active Varenicline + Placebo NRT			Placebo Varenicline + Active NRT			Active Cytisine + Placebo NRT			Placebo Cytisine + Active NRT		
**Event Description**	**Mild**	**Moderate**	**Severe**	**Mild**	**Moderate**	**Severe**	**Mild**	**Moderate**	**Severe**	**Mild**	**Moderate**	**Severe**
PANCREATITIS	0	0	1	0	0	0	0	0	0	0	0	0
PNEUMONIA	0	0	1	0	0	1	0	0	0	0	0	0
POST-INFARCTION DISORDERS	0	0	0	0	1	0	0	0	0	0	0	0
RESTLESSNESS	0	0	0	0	0	0	0	1	0	0	0	0
STRANGE DREAMS AND/OR NIGHTMARES	1	0	0	0	0	0	0	1	0	0	0	0
SWEATING	0	0	0	0	0	0	1	0	0	0	0	0
TACHYCARDIA	0	0	0	0	0	0	1	0	0	0	0	0
THROAT IRRITATION	1	0	0	1	2	0	0	0	0	2	0	0
THROAT PAIN	0	0	0	1	0	0	0	0	0	0	0	0
THROMBOCYTOPENIA	0	1	0	0	1	1	0	0	0	0	0	0
UTERINE BLEED	0	0	0	0	0	0	0	0	1	0	0	0
VOMITING	0	0	0	0	0	0	1	0	0	0	0	0

© 2022 Tindle HA et al. *JAMA Network Open.*

Serious Adverse Events								
	Active Varenicline + Placebo NRT		Placebo Varenicline + Active NRT		Active Cytisine + Placebo NRT		Placebo Cytisine + Active NRT	
**Event Description**	**Severe**	**Fatal**	**Severe**	**Fatal**	**Severe**	**Fatal**	**Severe**	**Fatal**
ACUTE ALCOHOL POISINING	0	0	0	2	0	1	0	0
ACUTE HEART FAILURE	0	1	0	1	0	0	0	0
ACUTE OTITIS, MASTIODITIS	0	0	0	0	1	0	0	0
HODGKIN'S DISEASE	0	1	0	0	0	0	0	0
MYOCARDIAL INFARCTION	0	0	2	0	0	0	0	0
OPIOID OVERDOSE	0	0	0	0	1	0	0	1
PANCREATITIS	1	0	0	0	0	0	0	0
PNEUMONIA	1	2	1	0	0	0	0	0
SEPSIS	0	0	0	0	0	1	0	0
TUBERCULOSIS	0	0	1	0	0	0	1	0
UTERINE BLEED	0	0	0	0	1	0	0	0

© 2022 Tindle HA et al. *JAMA Network Open.*

eTable 5. Post Hoc Analyses Examining No. of Heavy Drinking Days and Percent With Alcohol Abstinence by Smoking Status at 3, 6, and 12 mo

Trial Arm	**Smoking Abstinence[a] at 3, 6, 12 months**	
	**Yes**	**No**
**Heavy Drinking Days at 3 Months (Mean, SD)**		
Active Varenicline + Placebo NRT	N=24<br>0.9 (1.8)	N=76<br>2.4 (4.2)
Placebo Varenicline + Active NRT	N=13<br>0.7 (1.8)	N=86<br>2.3 (4.5)
Active Cytisine + Placebo NRT	N=26<br>0.6 (1.6)	N=74<br>1.8 (3.7)
Placebo Cytisine + Active NRT	N=22<br>0.8 (1.8)	N=79<br>2.9 (5.7)
Overall (all treatment arms)	N=85<br>0.7 (1.8)	N=315<br>2.3 (4.6)
**Alcohol Abstinence (PEth-verified) at 3 Months[b] n (%)**		
Active Varenicline + Placebo NRT	10 (41.7%)	13 (17.1%)
Placebo Varenicline + Active NRT	7 (53.8%)	12 (14.0%)
Active Cytisine + Placebo NRT	7 (26.9%)	15 (20.3%)
Placebo Cytisine + Active NRT	6 (27.3%)	14 (17.7%)
Overall (all treatment arms)	30 (35.3%)	54 (17.1%)
**Heavy Drinking Days at 6 Months (Mean, SD)**		
Active Varenicline + Placebo NRT	N=15<br>1.5 (3.9)	N=85<br>1.9 (3.9)
Placebo Varenicline + Active NRT	N=17<br>0.6 (1.5)	N=82<br>1.4 (2.8)
Active Cytisine + Placebo NRT	N=19<br>2.0 (5.4)	N=81<br>1.5 (3.1)
Placebo Cytisine + Active NRT	N=19<br>0.6 (1.6)	N=82<br>2.7 (5.5)
Overall (all treatment arms)	N=70<br>1.2 (3.7)	N=330<br>1.9 (4.0)
**Alcohol Abstinence (self-reported) at 6 Months n (%)**		
Active Varenicline + Placebo NRT	8 (53.3%)	20 (23.5%)
Placebo Varenicline + Active NRT	10 (58.8%)	25 (30.5%)
Active Cytisine + Placebo NRT	10 (52.6%)	28 (34.6%)
Placebo Cytisine + Active NRT	10 (52.6%)	24 (29.3%)
Overall (all treatment arms)	38 (54.3%)	97 (29.4%)
**Heavy Drinking Days at 12 Months (Mean, SD)**		
Active Varenicline + Placebo NRT	N=17<br>1.6 (4.8)	N=83<br>3.6 (6.8)
Placebo Varenicline + Active NRT	N=17<br>1.5 (3.9)	N=82<br>2.7 (5.6)
Active Cytisine + Placebo NRT	N=24<br>1.7 (4.7)	N=76<br>3.3 (6.7)
Placebo Cytisine + Active NRT	N=21<br>2.4 (4.6)	N=80<br>4.1 (7.8)
Overall (all treatment arms)	N=79<br>1.8 (4.7)	N=321<br>3.4 (6.8)

© 2022 Tindle HA et al. *JAMA Network Open.*

Trial Arm	Smoking Abstinence[a] at 3, 6, 12 months	
	**Yes**	**No**
**Alcohol Abstinence (self-reported) at 12 Months n (%)**		
Active Varenicline + Placebo NRT	6 (35.3%)	23 (27.7%)
Placebo Varenicline + Active NRT	9 (52.9%)	26 (31.7%)
Active Cytisine + Placebo NRT	13 (54.2%)	26 (34.2%)
Placebo Cytisine + Active NRT	9 (42.9%)	26 (32.5%)
Overall (all treatment arms)	37 (46.8%)	101 (31.5%)

[a] Smoking abstinence defined as self-reported 7-day point prevalence abstinence that is biochemically validated using a threshold of carbon monoxide (CO) < 10 ppm

[b] Alcohol abstinence defined as self-report of no alcohol use in past 30 days and PEth < 8 ng/mL

© 2022 Tindle HA et al. *JAMA Network Open.*
