# Supplementary material for: Effectiveness of Varenicline and Cytisine for Alcohol Use Reduction Among People With HIV and Substance Use: A Randomized Clinical Trial
Source: JAMA Netw Open. 2022 Aug 5;5(8):e2225129. doi: 10.1001/jamanetworkopen.2022.25129 (PMC9356316; doi:10.1001/jamanetworkopen.2022.25129)
Supplement: Supplement 3. — Data Sharing Statement [file jamanetwopen-e2225129-s003.pdf]

Data Sharing Statement

Tindle HA, Freiberg MS, Cheng DM, et al. Effectiveness of varenicline and cytisine for alcohol reduction among people with HIV and substance use. *JAMA Netw Open*. 2022;5(8):e2225129. doi:10.1001/jamanetworkopen.2022.25129

Data

Data available: Yes
Data types: Deidentified participant data
How to access data: Investigators may contact Sally Bendiks at Sally.Bendiks@BMC.org for more information on the process for submitting a proposal for approval to conduct secondary analyses for publication. As of 2-11-22 this process to date has involved the following steps: (1) the lead manuscript proposer works with the investigative team to develop a proposal and submits the finalized proposal for centralized analysis; (2) when results are available, the lead proposer writes the paper in conjunction with coinvestigators and submits for publication. For manuscripts proposed by investigators who were not part of the original study team, members of the original investigative team will participate in publications as co-authors.

Data collected for the study are available to interested investigators in the URBAN ARCH Repository: www.urbanarch.org

When available: With publication

Supporting Documents

Document types: None

Additional Information

Who can access the data: Researchers whose proposed use of the data has been approved
Types of analyses: Data
Mechanisms of data availability: Access to the data will be made with support of the investigative team. We will evaluate on a case-by-case basis whether a signed data access agreement is needed.
Any additional restrictions: n/a
